# Structure-Guided Design and Optimization of Covalent VHL-Targeted Sulfonyl Fluoride PROTACs

**DOI:** 10.1021/acs.jmedchem.3c02123

**Published:** 2024-03-13

**Authors:** Rishi R. Shah, Elena De Vita, Preethi S. Sathyamurthi, Daniel Conole, Xinyue Zhang, Elliot Fellows, Eleanor R. Dickinson, Carlos M. Fleites, Markus A. Queisser, John D. Harling, Edward W. Tate

**Affiliations:** † GSK, Medicines Research Centre, Stevenage, Hertfordshire SG1 2NY, U.K.; ‡ Department of Chemistry, Molecular Sciences Research Hub, Imperial College London, 82 Wood Lane, London W12 0BZ, U.K.; § Department of Biochemistry, School of Biological and Behavioural Sciences, Queen Mary University of London, 327 Mile End Road, London E1 4NS, U.K.; ∥ The Francis Crick Institute, 1 Midland Road, London NW1 1AT, U.K.

## Abstract

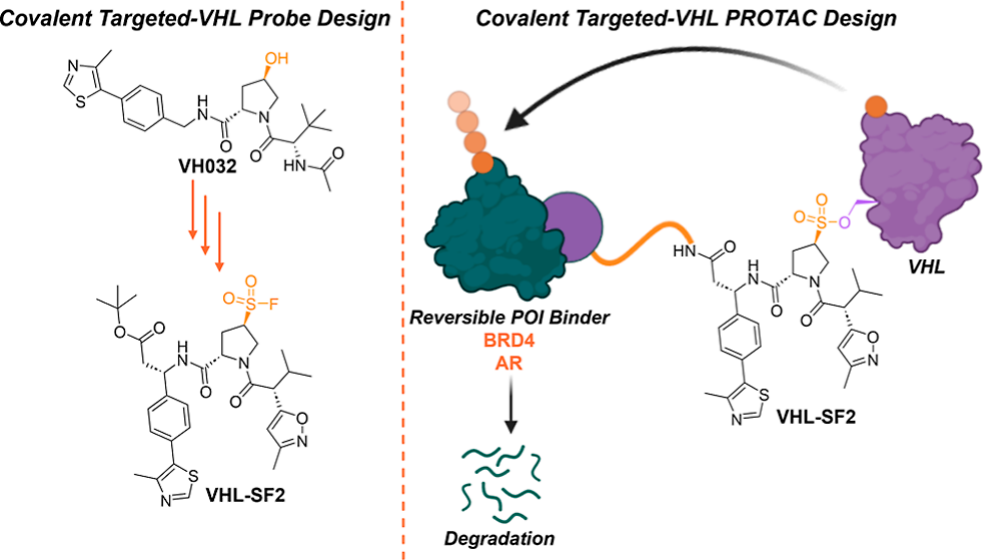

Proteolysis-targeting chimeras (PROTACs) are heterobifunctional molecules that have emerged as a therapeutic modality to induce targeted protein degradation (TPD) by harnessing cellular proteolytic degradation machinery. PROTACs which ligand the E3 ligase in a covalent manner have attracted intense interest; however, covalent PROTACs with a broad protein of interest (POI) scope have proven challenging to discover by design. Here, we report the structure-guided design and optimization of Von Hippel-Lindau (VHL) protein-targeted sulfonyl fluorides which covalently bind Ser110 in the HIF1α binding site. We demonstrate that their incorporation in bifunctional degraders induces targeted protein degradation of BRD4 or the androgen receptor without further linker optimization. Our study discloses the first covalent VHL ligands which can be implemented directly in bifunctional degrader design, expanding the substrate scope of covalent E3 ligase PROTACs.

## Introduction

The Von Hippel-Lindau (VHL) protein is among the most widely recruited E3 ligases in the PROTAC field. All potent small-molecule VHL binders reported to date feature a (*R*)-hydroxyproline motif,^1−4^ which forms an essential interaction with Ser110 in the HIF1α binding site of VHL but limits passive transport across the cell membrane.^5−8^ This motif is considered essential for VHL recognition and presents a challenge for the optimization of VHL PROTAC potency and cell uptake.

**Figure 1:**
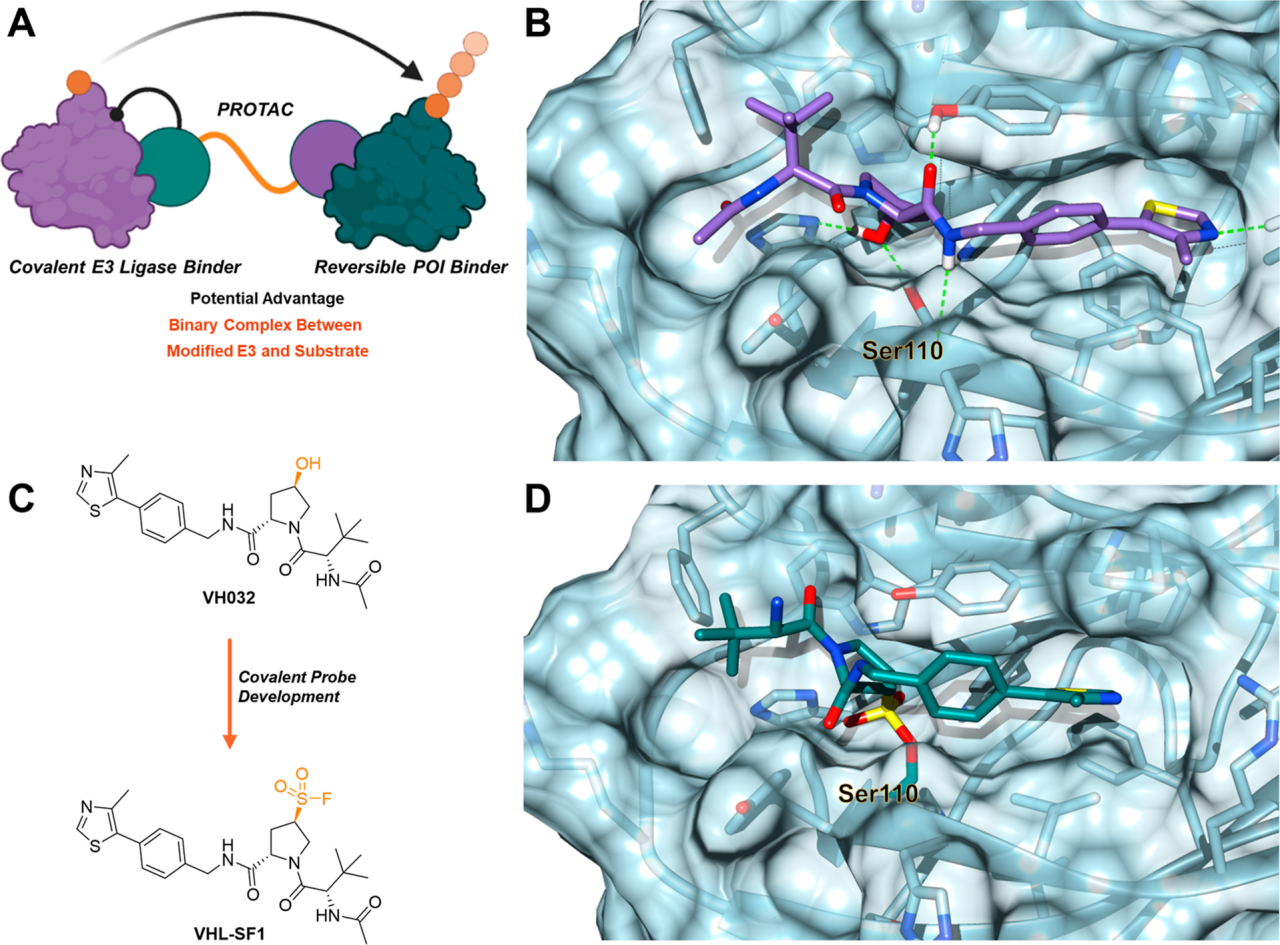
(A) Potential advantage of covalent PROTACs allowing transformation of the ternary complex into a simple binary interaction between modified E3 and substrate. (B) VH032/VHL cocrystal structure, illustrating the critical Ser110 interaction between **VH032** (purple) and VHL (PDB: 4W9H). (C) Structure of **VH032** and the replacement of the hydroxyproline moiety with a sulfonyl fluoride to generate **VHL-SF1**. (D) Docking of **VHL-SF1** (blue) within a VHL crystal structure (PDB: 4W9H).

PROTACs which ligand the E3 ligase in a covalent manner offer potential advantages over their reversible counterparts by transforming the ternary complex into a simple binary interaction between modified E3 and the substrate (Figure 1A).^9^ To date, covalent PROTACs have been reported for DCAF1, DCAF11, DCAF16, RNF4, RNF114, and FEM1B, each bearing a cysteine-targeting electrophilic warhead (e.g., chloroacetamide or α,β-unsaturated carbonyl) and have been discovered by screening rather than by design.^10−15^ Recently reported covalent CRBN E3 ligase binders bearing a sulfonyl fluoride show intriguing molecular glue activity, although they have yet to be incorporated in a covalent PROTAC.^16^

Herein, we document the design and optimization of the first rationally designed covalent VHL ligands and their incorporation in PROTACs for TPD applications. We demonstrate that the hydroxyproline motif of a known VHL binder can be replaced by a sulfonyl fluoride moiety, and through structure-guided optimization, we generated a ligand which covalently modifies Ser110 of VHL in the HIF1α binding site. We systematically assess VHL occupancy in recombinant proteins and live cells and the capacity of covalent PROTACs derived from this ligand to induce degradation of BRD4 and androgen receptor (AR). We suggest that this novel covalent VHL PROTAC paradigm will prove valuable for future studies of target engagement and optimization of the pharmacokinetic and pharmacodynamic properties of VHL-recruiting PROTACs.

## Results and Discussion

### First-Generation Sulfonyl Fluoride Covalent VHL Ligand

The binding mode of **VH032**, the most widely exploited VHL ligand in PROTACs to date, features a critical hydrogen bond to Ser110 through the (*R*)-hydroxyproline motif (Figure 1B).^8^ In our initial ligand designs, we sought to replace the hydroxy group with a sulfonyl fluoride, an electrophilic warhead featuring balanced reactivity, resistance to hydrolysis under physiological conditions, and the capacity to covalently modify proteins at varied nucleophilic residues beyond cysteine, including serine.^17,18^ We investigated how the hydroxyl replacement would perturb VHL binding by docking the prototype covalent ligand **VHL-SF1** (Figure 1C) in VHL derived from a VH032/VHL complex (PDB: 4W9H) (Figures 1D and S1).^2^ These models suggest that **VHL-SF1** has the potential to covalently bind Ser110, thereby maintaining some of the critical interactions observed in the hydroxyproline motif despite a degree of displacement of the remainder of the molecule (Figure S1), which may compromise the noncovalent interactions exhibited by **VH032**. We considered **VHL-SF1** a reasonable starting point to probe covalent VHL modification.

**Scheme 1:**
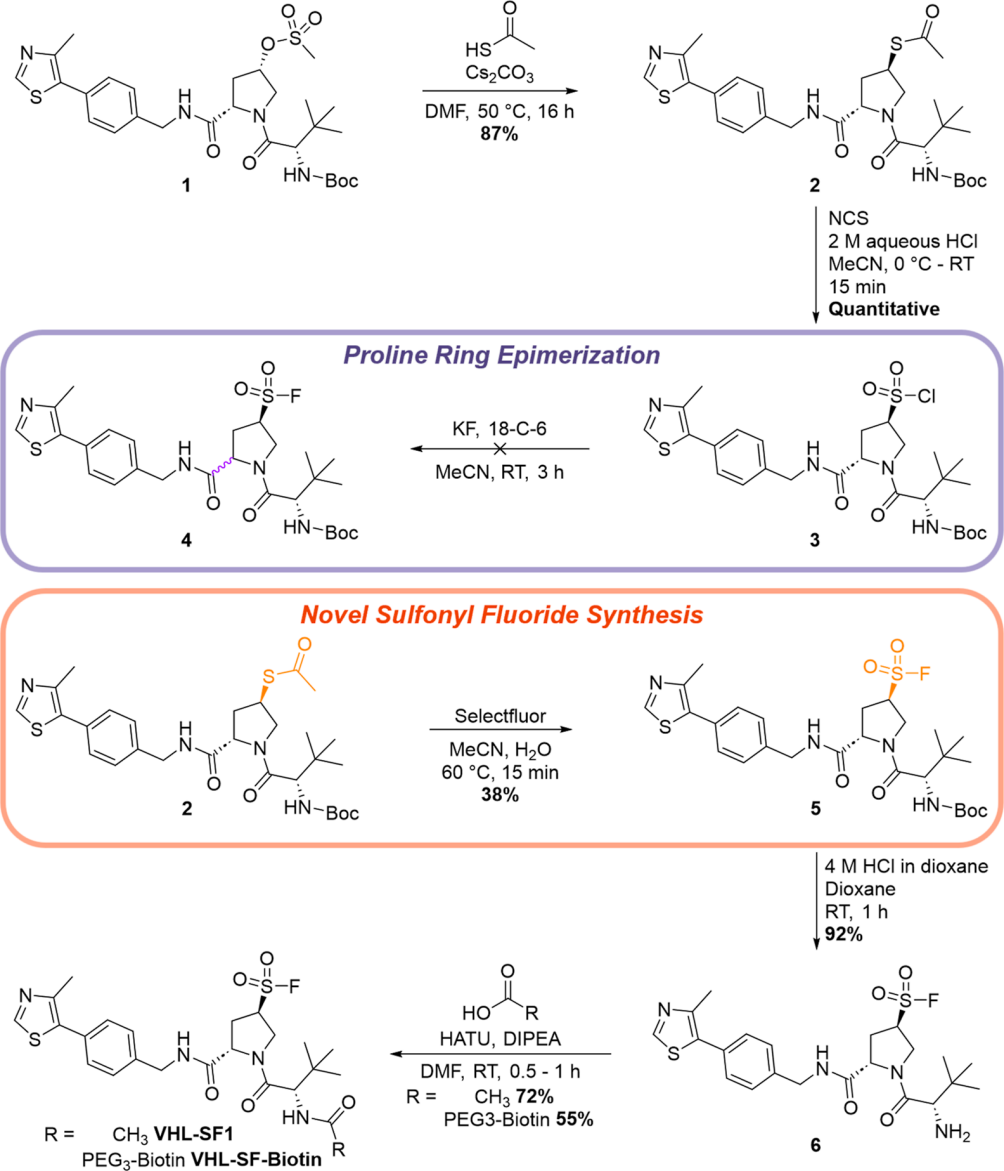
Development of a Novel Synthetic Route for the Introduction of the Sulfonyl Fluoride Moiety in **VHL-SF1** and **VHL-SF1-Biotin**

Synthesis of **VHL-SF1** commenced with the generation of (*S*)-hydroxyproline through a short sequence of reactions (Scheme S1). Displacement of mesylate **1** with thioacetate afforded compound **2**, which was converted to sulfonyl fluoride **4** via sulfonyl chloride **3** (Scheme 1). While this reaction generated the desired sulfonyl fluoride, epimerization of the proline ring occurred, resulting in a mixture of diastereomers detected by LC–MS analysis. To circumvent this, we developed novel reaction conditions to synthesize the desired sulfonyl fluoride in one step directly from thioacetate **2**, resulting exclusively in the desired stereoisomer **5**. A proposed mechanism for the novel sulfonyl fluoride transformation based on known analogous reactions is shown in Scheme S2.^19^ Following Boc-deprotection, the amine was acetylated to afford **VHL-SF1** or biotinylated to generate **VHL-SF1-Biotin**.

To assess the ability of **VHL-SF1** to covalently modify VHL, we initially developed a streptavidin shift assay in which we exposed recombinant human VCB, a stable complex of VHL protein with elongin C and elongin B, to **VHL-SF1-Biotin**. VHL biotinylation could then be directly quantified by the apparent shift in molecular weight observed when the sample was mixed with streptavidin and analyzed by an anti-VHL Western blot (Figure S2).^20^ Through this assay, we concluded that 10 μM **VHL-SF1-Biotin** modified 32% VHL following 2 h of incubation at room temperature, which was further confirmed to be concentration-dependent with respect to the probe (Figure S3).

Consistent with this modest reactivity, **VHL-SF1** at concentrations up to 100 μM was unable to displace a fluorophore-labeled HIF1α peptide, known to occupy both the **VH032** binding site and a second VHL domain, in a competitive fluorescence polarization (FP) assay (Figure S4). We next focused on developing a second generation covalent VHL binder with enhanced potency and occupancy.

### Second-Generation Sulfonyl Fluoride VHL Covalent Ligand

**Figure 2:**
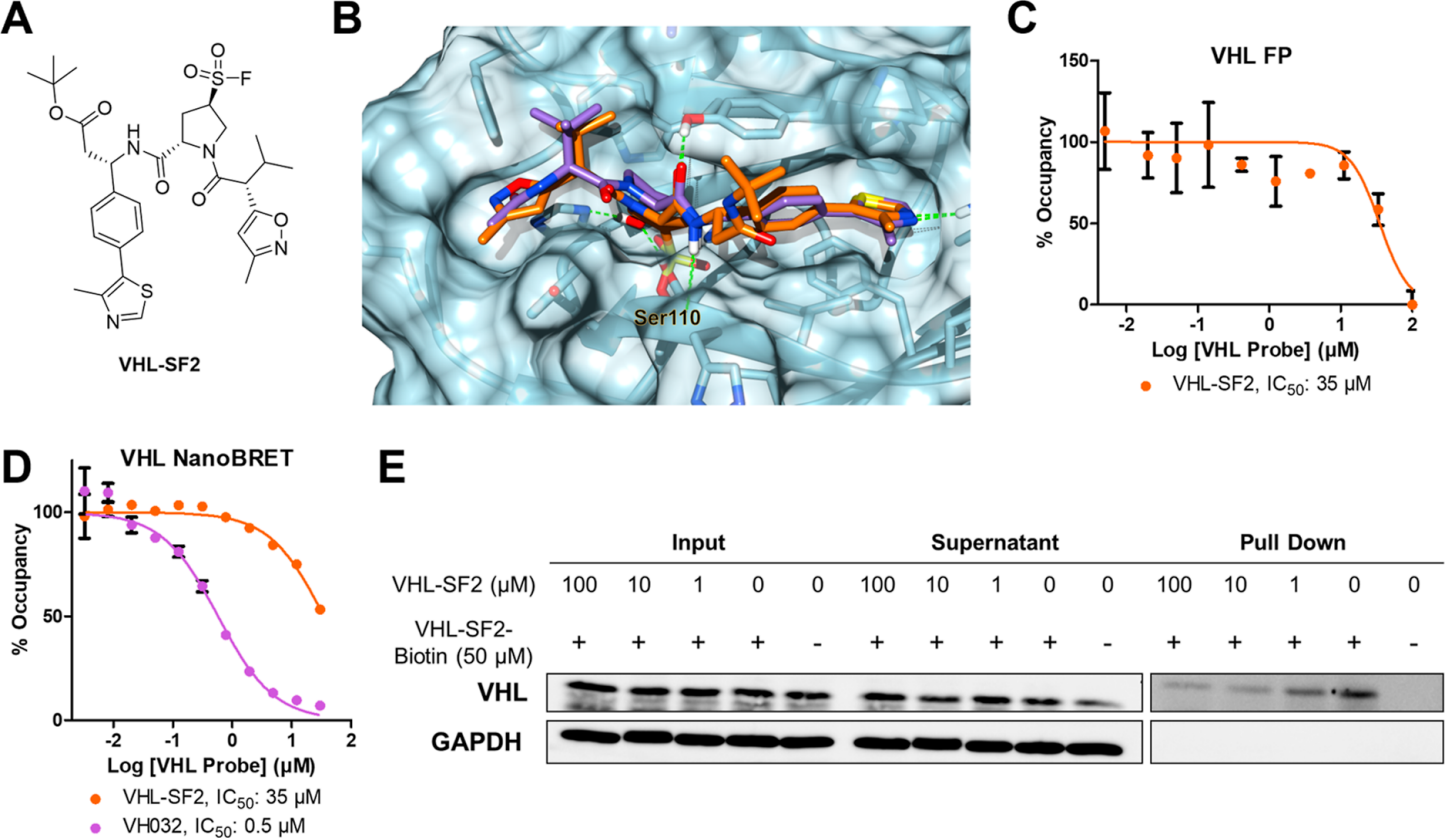
(A) Structure of **VHL-SF2**. (B) Docking of **VHL-SF2** (orange) within a crystal structure of VHL (PDB: 4W9H) superimposed onto **VH032** (purple). (C) Dose–response of **VHL-SF2** inhibition of the VCB and FAM-conjugated HIF1α-derived peptide interaction assessed by FP, following 2 h incubation between VCB and **VHL-SF2**. The data show the mean ± SEM (*n* = 3). (D) Cellular potency of **VHL-SF2** and **VH032** measured by a NanoBRET target engagement assay, following a 5 min incubation between VHL-NanoLuc HEK293 cells, **VHL-SF2**, NanoBRET VHL tracer ligand, and digitonin. The data show the mean ± SEM (*n* = 3). (E) Competition pull-down assay from live cells between **VHL-SF2-Biotin** and **VHL-SF2**. HEK293T cells were pretreated with DMSO or varying concentrations of **VHL-SF2** for 2 h at 37 °C. The cells were lysed and treated with DMSO or **VHL-SF2-Biotin** (50 μM) for 2 h at room temperature. A pull-down with streptavidin beads was conducted, after which the protein was resolved on SDS/PAGE. VHL and loading control GAPDH levels were visualized by Western blotting.

In order to improve covalent ligand potency, we reasoned that optimization of the groups peripheral to the hydroxyproline motif could provide enhanced affinity for VHL. Drawing inspiration from structure–activity studies of previously reported VHL binders,^4^ we examined docked poses for analogues of **VHL-SF1**, including **VHL-SF2**, in which the *tert*-leucine moiety was swapped for a methyl isoxazole and the ligation vector moved to the benzylic position (Figure 2A). In contrast to **VHL-SF1**, this analysis suggests that **VHL-SF2** may covalently bind Ser110 while maintaining many of the noncovalent interactions seen with **VH032** (Figure 2B). **VHL-SF2** was synthesized in 11 steps (Scheme S3).

Initially, we subjected **VHL-SF2-Biotin** to the streptavidin shift assay and observed a greater extent of VHL modification (44%) than for **VHL-SF1-Biotin** under the same conditions (Figure S2). Furthermore, **VHL-SF2** was able to displace the labeled peptide in the FP assay, with an apparent IC_50_ of 35 μM at 2 h, consistent with the predicted covalent occupancy of the VHL HIF1α binding site (Figure 2C). Intact LC–MS analysis confirmed single labeling of VHL by **VHL-SF2**, with 65% conversion at 24 h (Figure S5). Although we attempted several site ID identification experiments by digesting the labeled recombinant VHL, the Ser110-containing peptide was not detected in the samples treated with **VHL-SF2**. This observation is consistent with a change in peptide properties that is incompatible with mass spectrometry detection compared to untreated samples (Table S2). However, there was no evidence of modification of the protein at high sequence coverage (>80% on average) at any other site, offering indirect evidence of binding at the predicted site. Encouraged by this evidence for biochemical engagement of VHL, we explored engagement of VHL by **VHL-SF2** in live cells through a competition pull-down assay from HEK293T cells (Figure 2E). HEK293T cells were incubated with varying concentrations of **VHL-SF2** for 2 h at 37 °C, lysed, and treated with **VHL-SF2-Biotin** at 50 μM for 2 h, followed by pull-down on streptavidin beads. Elution under strongly denaturing conditions (5% β-mercaptoethanol in Laemmli Buffer for 10 min at 95 °C), SDS-PAGE, and Western blot analysis confirmed the pull-down of VHL by **VHL-SF2-Biotin**, consistent with expected covalent engagement, which could be dose-dependently competed by pretreatment with **VHL-SF2**.

Target engagement and cellular VHL binding potency were further confirmed through a NanoBRET target engagement assay, assessing inhibition of the interaction between VHL-NanoLuc and a cell-permeable fluorescent VHL tracer ligand in HEK293 cells (Figure 2D).^21^ In agreement with the FP assay, **VHL-SF2** inhibited the BRET signal with an IC_50_ of 35 μM, compared to >100 μM for **VHL-SF1** and 0.5 μM for **VH032**, consistent with intracellular HIF1α binding site occupancy. The reduced stability of the sulfonyl fluoride warhead at physiological pH at 40 °C (Table S1) could partially account for the significant reduction in potency observed in cell-based assays.

### VHL-SF2-Based PROTAC Induces Proteasome-Dependent TPD of BRD4

**Figure 3:**
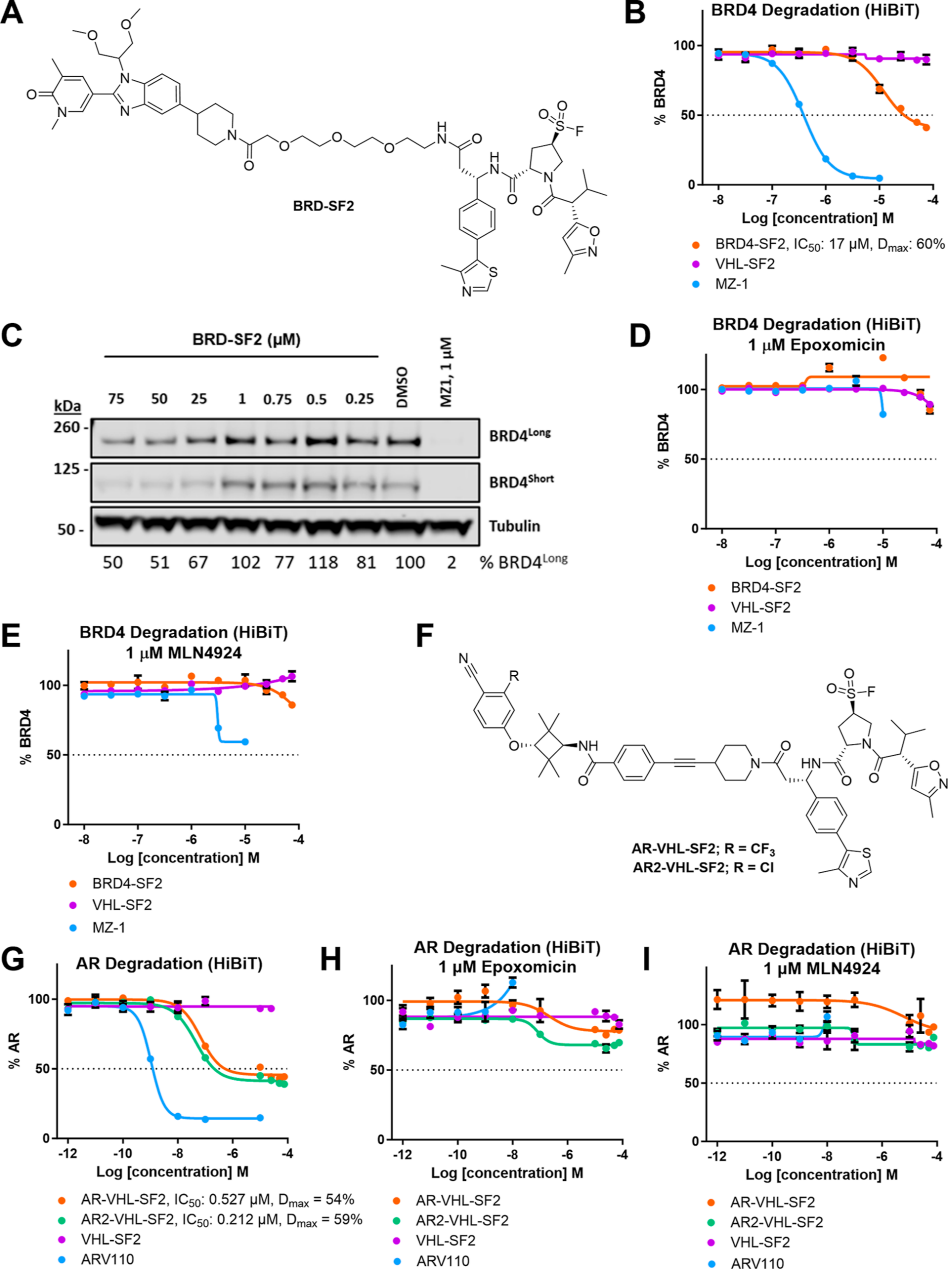
(A) Structure of **BRD-SF2**. (B) HEK293 HiBiT-BRD4 Cln3 cells were treated with **BRD-SF2**, **MZ-1**, or **VHL-SF2** dose-dependently (75 μM–1 pM) for 18 h. The data show representative assay from *n* = 4. (C) **BRD-SF2**-mediated degradation of endogenous BRD4. HEK293 cells were treated with DMSO, **MZ-1**, or varying concentrations of **BRD-SF2** for 18 h. BRD4 and loading control GAPDH levels were visualized by Western blotting. The data show representative Western blot from *n* = 3. (D) HEK293 HiBiT-BRD4 Cln3 cells were treated with epoxomicin (1 μM) for 3 h, followed by the respective PROTAC or VHL-SF2 dose-dependently (75–0.01 μM) for 18 h. The data show the mean ± SEM (*n* = 4). (E) HEK293 HiBiT-BRD4 Cln3 cells were treated with MLN4924 (1 μM) for 3 h, followed by the respective PROTAC or **VHL-SF2** dose-dependently (75–0.01 μM) for 18 h. The data show the mean ± SEM (*n* = 4). (F) Structure of **AR-VHL-SF2** and **AR2-VHL-SF2**. (G) AR-HiBiT LNCaP cells were treated with either **AR-VHL-SF2**, **AR2-VHL-SF2**, **ARV110**, or **VHL-SF2** dose-dependently (75 μM–1 pM) for 16 h. The data show the mean ± SEM (*n* = 4). (H) AR-HiBiT LNCaP cells were treated with epoxomicin (1 μM) for 3 h, followed by either **AR-VHL-SF2**, **AR2-VHL-SF2**, **ARV110**, or **VHL-SF2** dose-dependently (75 μM–1 pM) for 16 h. The data the show mean ± SEM (*n* = 3). (I) AR-HiBiT LNCaP cells were treated with MLN4924 (1 μM) for 3 h, followed by either **AR-VHL-SF2**, **AR2-VHL-SF2**, **ARV110**, or **VHL-SF2** dose-dependently (75 μM–1 pM) for 18 h. The data show the mean ± SEM (*n* = 3).

After validating VHL engagement by **VHL-SF2** both on isolated protein and in intact cells, we synthesized a BRD4-targeted PROTAC, **BRD-SF2**, incorporating **VHL-SF2** and a known BRD4 ligand (Figure 3A),^22^ and assessed target protein degradation using a BRD4 HiBiT assay.^21^ Pleasingly, this unoptimized PROTAC induced BRD4 degradation (DC_50_: 17.2 μM, *D*_max_: 60% at 18 h incubation), while **VHL-SF2** alone did not affect BRD4 levels (Figure 3B); **BRD-SF2** and **VHL-SF2** showed no evidence of cytotoxicity under these conditions (CellTiter-Glo assay, Figure S6A). Consistent with the BRD4 HiBiT assay, **BRD-SF2** also induced endogenous BRD4 degradation to a similar extent across the concentrations tested (BRD4 long isoform *D*_max_: 50%, Figures 3C and S7).

BRD4 was not depleted in the presence of **BRD-SF2** when treated with inhibitors of either proteasome activity or NEDDylation (epoxomicin and MLN4924, respectively), supporting a proteasome- and Cullin E3 ligase-dependent mechanism consistent with recruitment of VHL (Figure 3D,E).

### **VHL-SF2**-Based PROTACs Induce TPD of AR

To further confirm the versatility of **VHL-SF2**’s ability to recruit VHL to induce target protein degradation when incorporated into a PROTAC, we synthesized two AR ligand-derived **VHL-SF2** conjugates, **AR-VHL-SF2** and **AR2-VHL-SF2**, based on known AR ligands with attractive cellular potency that have previously been incorporated into AR-bifunctional degraders (Figure 3F).^23,24^ These compounds were assessed for induced AR degradation using an AR HiBiT assay in LNCaP prostate cancer cells, in which endogenous AR was tagged with a HiBiT peptide (Figure 3G). Both **AR-VHL-SF2** and **AR2-VHL-SF2** induced AR degradation (**AR-VHL-SF2** DC_50_ = 0.527 μM, *D*_max_ = 54%; **AR2-VHL-SF2** DC_50_ = 0.212 μM, *D*_max_ = 59%), in addition to Arvinas’s AR-degrading clinical candidate, **ARV110**, which was used as a positive control. Both **AR-VHL-SF2**- and **AR2-VHL-SF2**-mediated protein degradation were proteasome- and E3 ligase-dependent based on AR degradation blockade in the presence of either epoxomicin or MLN4924 (Figure 3H,I) and did not exhibit cytotoxicity under these conditions (CellTiter-Glo assay, Figure S6B).

### Washout Experiments Reveal an Advantage for Covalent VHL PROTACs

**Figure 4:**
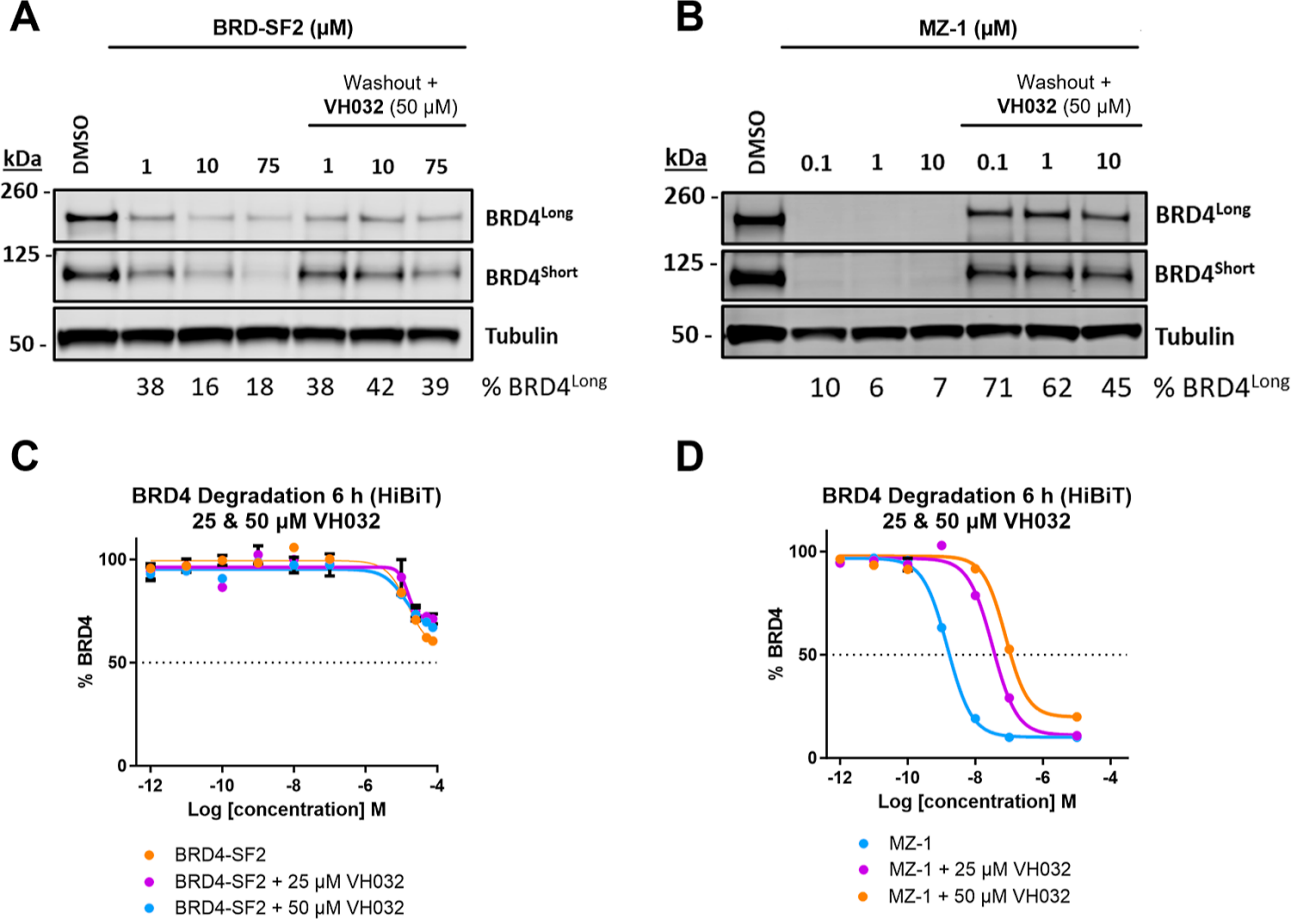
(A) HEK293 HiBiT-BRD4 Cln3 cells were treated with **BRD-SF2** (75–1 μM) for 5 h, followed by PBS washing and 24 h recovery in the presence of excess **VH032** (50 μM). BRD4 and loading control Tubulin levels were visualized by Western blotting. The data show representative Western blot from *n* = 2. (B) HEK293 HiBiT-BRD4 Cln3 cells were treated with **MZ-1** (10–0.1 μM) for 5 h, followed by PBS washing and 24 h recovery in the presence of excess **VH032** (50 μM). BRD4 and loading control Tubulin levels were visualized by Western blotting. The data show representative Western blot from *n* = 2. (C) HEK293 HiBiT-BRD4 Cln3 cells were treated with **BRD-SF2** (*n* = 4) or **BRD-SF2** in the presence of **VH032** (25 or 50 μM, *n* = 2) for 6 h. (D) HEK293 HiBiT-BRD4 Cln3 cells were treated with **MZ-1** (*n* = 4) or **MZ-1** in the presence of **VH032** (25 or 50 μM, *n* = 2) for 6 h.

To further investigate the potential advantage of a covalent VHL ligand, we performed a head-to-head comparison of **BRD-SF2** and **MZ-1** in a washout experiment. In this assay, we incubated HEK293 HiBiT-BRD4 Cln3 cells with varying concentrations of each PROTAC for 5 h, followed by 24 h recovery after treatment washout. Despite the significant disparity in degradation efficiency between the two compounds, we observed a similar relative decrease in BRD4 degradation (Figure S8). To prevent extended degradation due to residual PROTAC not being washed out, we introduced an excess of the VHL ligand **VH032**, which binds potently to VHL and blocks degradation by displacing any residual PROTAC which is not covalently bound.^25^ Indeed, in this experiment, **BRD-SF2** showed a reduced relative decrease in degradation activity compared to **MZ-1** (Figure 4A,B), quantified at 27% vs 48%, respectively (Figure S9C). The BRD4 HiBiT assay in the presence of competing concentrations of **VH032** orthogonally confirmed that BRD4 degradation by **MZ-1** was significantly more affected by the presence of the competitor at 6 h (Figure 4E,F). These results further support the covalent mechanism of action of **BRD-SF2** and highlight the potential to prolong degradation activity following the removal of free PROTAC or in the presence of competing binders.

## Conclusions

To our knowledge, this is the first report of a VHL ligand that lacks the hydroxyproline motif and engages covalently with VHL via a sulfonyl fluoride moiety, and the first covalent E3 ligase binder developed by design rather than screening. When incorporated into bifunctional degraders, the resulting sulfonyl fluoride-based PROTACs are capable of inducing proteasome- and ubiquitin ligase-dependent TPD of both BRD4 and AR. However, the observed degradation efficiency does not currently match optimized noncovalent PROTACs (e.g., **MZ-1**), and these compounds would require further medicinal chemistry optimization, including to improve stability under physiological conditions. Nonetheless, it is encouraging that functional degradation is observed for these first-generation, unoptimized prototypes, with the potential to expand the substrate scope of E3 ligase-covalent PROTACs to the >20 target proteins previously reported to be degradable with VHL-recruiting PROTACs. Interestingly, the degradation efficiency of these PROTACs was uncoupled from the low occupancy of VHL, and we obtained submicromolar degraders of AR without further optimization of the warhead. Our washout experiments provide insight into the potential pharmacodynamic advantages of covalent VHL PROTACs, which show relatively sustained degradation in the presence of a VHL binder (**VH032**) compared to more potent PROTACs (e.g., **MZ-1**), consistent with the persistence of covalent VHL-adducts. This finding points to the advantages of covalent E3 ligase recruiters in terms of catalytic efficiency and prolonged efficacy of a predicted binary complex compared to a standard ternary complex for noncovalent PROTACs. In the future, the effects of this adduct population on the natural substrates of VHL should be further investigated by whole proteomics to determine whether minimal occupancy could be sufficient to promote TPD without altering VHL function.

In summary, our work paves the way for additional structural modifications at the hydroxyproline center, which could ultimately result in improved pharmacokinetic and pharmacodynamic properties in PROTACs targeting VHL. This work benchmarks the covalent recruitment of VHL for TPD and provides the basis for medicinal chemistry campaigns to further optimize the covalent VHL ligand to enhance potency and stability. We also report novel bifunctional degraders of BRD4 and AR which could be used as starting points for the development of PROTACs with improved pharmacokinetic and pharmacodynamic properties.

## Experimental Section

All reagents were used as received from commercial sources (Sigma-Aldrich, Enamine, Combi-blocks, etc.), unless otherwise stated. In all syntheses, anhydrous solvents were used, and commercially available HPLC grade solvents were used for workup and isolation procedures, unless otherwise stated. All compounds tested in biological assays are >93% pure by HPLC analysis. For general methods (NMR, LC–MS, HRMS, MDAP, LC), see Supporting Information.

### Synthesis of **VHL-SF1** (Scheme S1)

#### *Tert*-Butyl (2*S*,4*S*)-4-hydroxy-2-((4-(4-methylthiazol-5-yl)benzyl)carbamoyl)pyrrolidine-1-carboxylate (**9**)

HATU (4.93 g, 12.97 mmol) was added to a stirred solution of (2*S*,4*S*)-1-(*tert*-butoxycarbonyl)-4-hydroxypyrrolidine-2-carboxylic acid (2 g, 8.65 mmol), DIPEA (3.02 mL, 17.30 mmol), and (4-(4-methylthiazol-5-yl)phenyl)methanamine (1.77 g, 8.65 mmol) in DMF (9.25 mL), and the reaction mixture was stirred at room temperature for 1 h within a sealed vessel. The reaction mixture was diluted with water (10 mL) and extracted with DCM (3 × 10 mL). The organic layers were combined and passed through a hydrophobic frit, and the solvent was removed in vacuo. The residue was purified by reverse-phase column chromatography (15–85% MeCN in H_2_O + 0.1% NH_4_HCO_3_, 330 g C18, 10 CV) to afford *tert*-butyl (2*S*,4*S*)-4-hydroxy-2-((4-(4-methylthiazol-5-yl)benzyl)carbamoyl)pyrrolidine-1-carboxylate (3.431 g, 8.22 mmol, 95% yield) as a yellow solid. ^**1**^**H NMR** (400 MHz, DMSO-*d*_6_): δ = 8.98 (1H, s), 8.50 (1H, t, *J* = 6.2 Hz), 7.37–7.44 (4H, m), 5.18–5.30 (1H, m), 4.33–4.43 (2H, m), 4.24–4.31 (1H, m), 4.08–4.14 (1H, m), 3.50 (1H, dd, *J* = 10.8, 5.4 Hz), 3.17–3.27 (1H, m), 2.44 (3H, s), 2.30–2.37 (1H, m), 1.73–1.83 (1H, m), 1.41 (3H, s), 1.26 (6H, s); ^**13**^**C NMR** (151 MHz, DMSO-*d*_6_): δ = 173.4, 153.8, 152.0, 151.9, 148.3, 139.8, 129.3 (2C), 129.2, 128.6 (2C), 127.9, 79.2, 68.5, 59.5, 54.9, 42.4, 39.2, 38.2, 28.4, 16.3; **LCMS** (Method B): *t*_R_ = 0.88 min, [M + H]^+^, 418, (93% purity); **HRMS** (C_21_H_28_N_3_O_4_S) [M + H]^+^ requires 418.1801; found [M + H]^+^, 418.1805.

#### (2*S*,4*S*)-4-Hydroxy-*N*-(4-(4-methylthiazol-5-yl)benzyl)pyrrolidine-2-carboxamide, 2HCl (**10**)

4 M HCl in Dioxane (50.7 mL, 203 mmol) was added to *tert*-butyl (2*S*,4*S*)-4-hydroxy-2-((4-(4-methylthiazol-5-yl)benzyl)carbamoyl)pyrrolidine-1-carboxylate (3.388 g, 8.11 mmol), and the reaction mixture was stirred at room temperature for 2 h. The solvent was removed in vacuo to afford (2*S*,4*S*)-4-hydroxy-*N*-(4-(4-methylthiazol-5-yl)benzyl)pyrrolidine-2-carboxamide, 2HCl (3.017 g, 7.73 mmol, 95% yield) as an ivory solid. ^**1**^**H NMR** (400 MHz, DMSO-*d*_6_): δ = 8.98, 10.00–10.18 (1H, m), 9.10 (1H, t, *J* = 6.2 Hz), 9.04 (1H, s), 8.52–8.67 (1H, m), 7.35–7.51 (4H, m), 4.35–4.48 (4H, m), 4.22–4.32 (1H, m), 3.19–3.29 (1H, m) 3.10–3.18 (1H, m) 2.46 (3H, s), 1.94–2.03 (1H, m); ^**13**^**C NMR** (101 MHz, DMSO-*d*_6_): δ = 168.2, 152.1, 146.9, 138.8, 131.5, 129.7, 128.9 (2C), 127.8 (2C), 68.4, 57.8, 52.3, 42.2, 38.2, 15.5; **LCMS** (Method A): *t*_R_ = 0.35 min, [M + H]^+^, 318, (95% purity); **HRMS** (C_16_H_20_N_3_O_2_S) [M + H]^+^ requires 318.1276; found [M + H]^+^, 318.1278.

#### *Tert*-Butyl ((*S*)-1-((2*S*,4*S*)-4-Hydroxy-2-((4-(4-methylthiazol-5-yl)benzyl)carbamoyl)pyrrolidin-1-yl)-3,3-dimethyl-1-oxobutan-2-yl)carbamate (**11**)

HATU (4.36 g, 11.48 mmol) was added to a stirred solution of (2*S*,4*S*)-4-hydroxy-*N*-(4-(4-methylthiazol-5-yl)benzyl)pyrrolidine-2-carboxamide, 2HCl (2.987 g, 7.65 mmol), DIPEA (4.01 mL, 22.96 mmol), and (*S*)-2-((*tert*-butoxycarbonyl)amino)-3,3-dimethylbutanoic acid (1.770 g, 7.65 mmol) in DMF (15.31 mL), and the reaction mixture was stirred at room temperature for 1 h. The reaction mixture was diluted with water (75 mL) and extracted with DCM (3 × 75 mL). The organic layers were combined and passed through a hydrophobic frit, and the solvent was concentrated in vacuo. The residue was purified by reverse-phase column chromatography (20–85% MeCN in H_2_O + 0.1% NH_4_HCO_3_, 330 g C18, 10 CV) to afford *tert*-butyl ((*S*)-1-((2*S*,4*S*)-4-hydroxy-2-((4-(4-methylthiazol-5-yl)benzyl)carbamoyl)pyrrolidin-1-yl)-3,3-dimethyl-1-oxobutan-2-yl)carbamate (2.873 g, 5.41 mmol, 71% yield) as an off-white foam. ^**1**^**H NMR** (400 MHz, DMSO-*d*_6_): δ = 8.99 (1H, s), 8.66 (1H, br t, *J* = 5.5 Hz), 7.37–7.43 (4H, m), 6.59 (1H, br d, *J* = 8.4 Hz), 5.44 (1H, br d, *J* = 7.0 Hz), 4.44 (1H, dd, *J* = 15.77, 6.6 Hz), 4.40 (1H, br dd, *J* = 8.4, 6.2 Hz) 4.27 (1H, dd, *J* = 15.8, 5.5 Hz), 4.21–4.24 (1H, m), 4.11 (1H, br d, *J* = 8.4 Hz), 3.86–3.94 (1H, m), 3.41–3.47 (1H, m), 2.45 (3H, s), 2.32–2.38 (1H, m), 1.71–1.78 (1H, m), 1.38 (9H, s), 0.95 (9H, s); ^**13**^**C NMR** (151 MHz, DMSO-*d*_6_): δ = 172.4, 170.2, 155.6, 151.4, 147.7, 139.1, 131.1, 129.7, 128.7 (2C) 127.4 (2C), 78.1, 69.1, 58.5, 55.6, 54.9, 41.8, 36.8, 34.7, 28.2 (3C), 26.3 (3C), 15.9; **LCMS** (Method B): *t*_R_ = 1.04 min, [M + H]^+^, 532, (98% purity); **HRMS** (C_27_H_39_N_4_O_5_S) [M + H]^+^ requires 531.2641; found [M + H]^+^, 531.2642.

#### (3*S*,5*S*)-1-((*S*)-2-((*tert*-Butoxycarbonyl)amino)-3,3-dimethylbutanoyl)-5-((4-(4-methylthiazol-5-yl)benzyl)carbamoyl)-pyrrolidin-3-yl Methanesulfonate (**1**)

Mesyl chloride (0.501 mL, 6.44 mmol) was added dropwise to a stirred solution of *tert*-butyl ((*S*)-1-((2*S*,4*S*)-4-hydroxy-2-((4-(4-methylthiazol-5-yl)benzyl)carbamoyl)pyrrolidin-1-yl)-3,3-dimethyl-1-oxobutan-2-yl)carbamate (2.846 g, 5.36 mmol) and triethylamine (0.897 mL, 6.44 mmol) in DCM (17.88 mL) over an ice–water bath, and the reaction mixture was stirred at room temperature for 30 min under a nitrogen atmosphere. The reaction mixture was washed with 5% citric acid (50 mL) followed by water (50 mL), the organic layer was passed through a hydrophobic frit, and the solvent was removed in vacuo. The residue was purified by normal-phase column chromatography (100% cyclohexane, 2 CV followed by 100% EtOAc, 330 g SiO_2_, 10 CV) to afford (3*S*,5*S*)-1-((*S*)-2-((*tert*-butoxycarbonyl)amino)-3,3-dimethylbutanoyl)-5-((4-(4-methylthiazol-5-yl)benzyl)carbamoyl)-pyrrolidin-3-yl methanesulfonate (2.954 g, 4.85 mmol, 90% yield) as a white foam. ^**1**^**H NMR** (400 MHz, DMSO-*d*_6_): δ = 8.99 (1H, s) 8.43 (1H, br t, *J* = 5.7 Hz) 7.37–7.44 (4H, m) 6.65 (1H, br d, *J* = 8.9 Hz) 5.23–5.36 (1H, m) 4.49 (1H, dd, *J* = 8.9, 5.9 Hz) 4.35 (2H, dd, *J* = 5.7, 2.7 Hz) 4.19–4.28 (1H, m) 4.11 (1H, br d, *J* = 8.9 Hz) 3.71 (1H, dd, *J* = 11.3, 4.9 Hz) 3.23 (3H, s) 2.59–2.69 (1H, m) 2.45 (3H, s) 2.08–2.16 (1H, m) 1.39 (9H, s) 0.90–0.99 (9H, m); ^**13**^**C NMR** (101 MHz, DMSO-*d*_6_): δ = 170.4, 170.1, 155.6, 151.4, 147.7, 139.2, 131.1, 129.7, 128.7 (2C), 127.5 (2C), 78.1, 77.8, 59.7, 58.7, 57.8, 54.8, 52.6, 41.8, 37.6, 34.8, 28.1 (2C), 26.3 (2C), 15.9, 14.0; **LCMS** (Method A): *t*_R_ = 1.06 min, [M + H]^+^, 609, (100% purity); **HRMS** (C_28_H_40_N_4_O_7_S_2_) [M + H]^+^, requires 609.2417; found [M + H]^+^, 609.2415.

#### *S*-((3*R*,5*S*)-1-((*S*)-2-((*tert*-Butoxycarbonyl)amino)-3,3-dimethylbutanoyl)-5-((4-(4-methylthiazol-5-yl)benzyl)carbamoyl)pyrrolidin-3-yl) Ethanethioate (**2**)

Thioacetic acid (0.445 mL, 6.20 mmol) was added to a stirred solution of Cs_2_CO_3_ (1.010 g, 3.10 mmol) and in DMF (11.55 mL) under a nitrogen atmosphere, and the reaction mixture was stirred at room temperature for 30 min within a sealed vessel. (3*S*,5*S*)-1-((*S*)-2-((*tert*-Butoxycarbonyl)amino)-3,3-dimethylbutanoyl)-5-((4-(4-methylthiazol-5-yl)benzyl)carbamoyl)pyrrolidin-3-yl methanesulfonate (2.904 g, 4.77 mmol) in DMF (4.35 mL) was added, and the sealed reaction mixture was stirred at 50 °C for 24 h. The solvent was removed in vacuo, and the residue was diluted with ethyl acetate (100 mL) and washed with saturated aqueous sodium hydrogen carbonate (100 mL), followed by water (100 mL), and the organic layer was passed through a hydrophobic frit, and the solvent was removed in vacuo. The residue was purified by normal-phase column chromatography (0–100% EtOAc in cyclohexane, 330 g SiO_2_, 10 CV) to afford *S*-((3*R*,5*S*)-1-((*S*)-2-((*tert*-butoxycarbonyl)amino)-3,3-dimethylbutanoyl)-5-((4-(4-methylthiazol-5-yl)benzyl)carbamoyl)pyrrolidin-3-yl) ethanethioate (2.435 g, 4.14 mmol, 87% yield) as an off-white solid. ^**1**^**H NMR** (400 MHz, DMSO-*d*_6_): δ = 8.99 (1H, s), 8.57 (1H, br t, *J* = 5.7 Hz), 7.38–7.41 (4H, m), 6.65 (1H, br d, *J* = 9.4 Hz), 4.47 (1H, br t, *J* = 7.1 Hz), 4.36–4.44 (1H, m), 4.22–4.32 (1H, m), 3.98–4.12 (3H, m), 3.71–3.80 (1H, m), 2.45 (3H, s), 2.34 (3H, s), 2.22–2.31 (1H, m), 2.10–2.19 (1H, m), 1.39 (9H, s), 0.94 (9H, s); ^**13**^**C NMR** (101 MHz, DMSO-*d*_6_): δ = 194.9, 171.0, 169.8, 155.6, 151.4, 147.7, 139.2, 131.1, 129.7, 128.7 (2C), 127.4 (2C), 78.1, 58.6, 58.5, 53.1, 41.6, 40.9, 34.7, 34.6, 30.5, 28.1 (3C), 26.2 (3C), 15.9; **LCMS** (Method A): *t*_R_ = 1.19 min, [M + H]^+^, 589, (98% purity); **HRMS** (C_29_H_41_N_4_O_5_S_2_) [M + H]^+^ requires 589.2518; found [M + H]^+^, 589.2515.

#### *tert*-Butyl ((2*S*)-1-((4*R*)-4-(Fluorosulfonyl)-2-((4-(4-methylthiazol-5-yl)benzyl)carbamoyl)pyrrolidin-1-yl)-3,3-dimethyl-1-oxobutan-2-yl)carbamate (**4**)

2 M aqueous HCl (42.5 μL, 0.085 mmol) was added dropwise to a stirred solution of NCS (91 mg, 0.679 mmol) in MeCN (233 μL) over an ice–water bath, and the reaction mixture was stirred over an ice–water bath for 15 min. *S*-((3*R*,5*S*)-1-((*S*)-2-((*tert*-Butoxycarbonyl)amino)-3,3-dimethylbutanoyl)-5-((4-(4-methylthiazol-5-yl)benzyl)carbamoyl)pyrrolidin-3-yl) ethanethioate (100 mg, 0.170 mmol) in MeCN (50 μL) was added dropwise, and the reaction mixture was stirred at room temperature for 20 min. The reaction mixture was diluted with DCM (10 mL) and washed with brine (3 × 5 mL), the organic layer was passed through a hydrophobic frit, and the solvent was removed in vacuo to afford *tert*-butyl ((*S*)-1-((2*S*,4*R*)-4-(chlorosulfonyl)-2-((4-(4-methylthiazol-5-yl)benzyl)carbamoyl)pyrrolidin-1-yl)-3,3-dimethyl-1-oxobutan-2-yl)carbamate (104 mg, 0.170 mmol, 100% yield) as a yellow oil. Potassium fluoride (39.4 mg, 0.678 mmol) and 18-crown-6 (179 mg, 0.678 mmol) were added to a stirred solution of *tert*-butyl ((*S*)-1-((2*S*,4*R*)-4-(chlorosulfonyl)-2-((4-(4-methylthiazol-5-yl)benzyl)carbamoyl)pyro-lidin-1-yl)-3,3-dimethyl-1-oxobutan-2-yl)carbamate (104 mg, 0.170 mmol) in MeCN (848 μL) under a nitrogen atmosphere, and the reaction mixture was sealed and stirred at room temperature for 1 h. The reaction was abandoned due to the racemisation of the proline ring.

#### *tert*-Butyl ((*S*)-1-((2*S*,4*R*)-4-(Fluorosulfonyl)-2-((4-(4-methylthiazol-5-yl)benzyl)carbamoyl)pyrrolidin-1-yl)-3,3-dimethyl-1-oxobutan-2-yl)carbamate (**5**)

Selectfluor (451 mg, 1.274 mmol), *S*-((3*R*,5*S*)-1-((*S*)-2-((*tert*-butoxycarbonyl)amino)-3,3-dimethylbutanoyl)-5-((4-(4-methylthiazol-5-yl)benzyl)carbamoyl)pyrrolidin-3-yl)ethanethioate (100 mg, 0.170 mmol) in acetonitrile (1544 μL) and water (154 μL) were sealed within a vessel and heated in a Biotage Initiator microwave for 15 min at 60 °C using a normal absorption setting. The reaction mixture was allowed to cool to room temperature. The reaction mixture was diluted with brine (2 mL) and extracted with DCM (3 × 10 mL). The organic layers were combined and passed through a hydrophobic frit, and the solvent was removed in vacuo. The residue was purified by normal-phase column chromatography (0–100% EtOAc in cyclohexane, 40 g SiO_2_, 12 CV) to afford *tert*-butyl ((*S*)-1-((2*S*,4*R*)-4-(fluorosulfonyl)-2-((4-(4-methylthiazol-5-yl)benzyl)carbamoyl)pyrrolidin-1-yl)-3,3-dimethyl-1-oxobutan-2-yl)carbamate (38 mg, 0.064 mmol, 38% yield) as a white foam. ^**1**^**H NMR** (400 MHz, DMSO-*d*_6_): δ = 8.98 (1H, s), 8.69 (1H, br t, *J* = 5.9 Hz), 7.40 (4H, s), 6.77 (1H, br d, *J* = 9.4 Hz), 4.88–4.99 (1H, m), 4.62 (1H, br t, *J* = 7.9 Hz), 4.54 (1H, br d, *J* = 12.3 Hz), 4.42 (1H, dd, *J* = 15.8, 6.4 Hz), 4.23–4.30 (1H, m), 4.16 (2H, br d, *J* = 9.4 Hz), 2.65–2.77 (2H, m), 2.44 (3H, s), 1.38 (9H, s), 0.94 (9H, s); ^**13**^**C NMR** (151 MHz, DMSO-*d*_6_): δ = 170.3, 170.0, 155.8, 151.4, 147.7, 139.0, 131.1, 129.8, 128.7 (2C), 127.5 (2C), 78.3, 59.9 (br d, *J* = 14.37 Hz), 58.5, 58.3, 47.9, 41.7, 34.5, 30.1, 28.1, 26.2 (3C), 15.9; ^**19**^**F NMR** (376 MHz, DMSO-*d*_6_): δ = 47.66 (1F, s) **LCMS** (Method A): *t*_R_ = 1.18 min, [M + H]^+^, 497 (Boc-deprotected fragment), (99% purity); **HRMS** (C_27_H_38_FN_4_O_6_S_2_) [M + H]^+^ requires 597.2217; found [M + H]^+^, 597.2219.

#### (3*R*,5*S*)-1-((*S*)-2-Amino-3,3-dimethylbutanoyl)-5-((4-(4-methylthiazol-5-yl)benzyl)carbamoyl)-pyrrolidine-3-sulfonyl fluoride, 2HCl (**6**)

4 M HCl in 1,4-dioxane (356 μL, 1.424 mmol) was added to a stirred solution of *tert*-butyl ((*S*)-1-((2*S*,4*R*)-4-(fluorosulfonyl)-2-((4-(4-methylthiazol-5-yl)benzyl)carbamoyl)pyrrolidin-1-yl)-3,3-dimethyl-1-oxobutan-2-yl)carbamate (34 mg, 0.057 mmol) in 1,4-dioxane (114 μL), and the reaction mixture was stirred at room temperature for 1 h. The solvent was removed in vacuo to afford (3*R*,5*S*)-1-((*S*)-2-amino-3,3-dimethylbutanoyl)-5-((4-(4-methylthiazol-5-yl)benzyl)carbamoyl)pyrrolidine-3-sulfonyl fluoride, 2HCl (30 mg, 0.053 mmol, 92% yield) as a white solid. ^**1**^**H NMR** (400 MHz, DMSO-*d*_6_): δ = 9.01 (1H, s), 8.86 (1H, t, *J* = 5.9 Hz), 8.12–8.32 (4H, m), 7.41 (3H, d, *J* = 2.5 Hz), 4.99–5.08 (1H, m), 4.74 (1H, t, *J* = 7.9 Hz), 4.54 (1H, dd, *J* = 13.0, 2.7 Hz), 4.40–4.48 (1H, m), 4.28 (1H, dd, *J* = 15.8, 5.4 Hz), 4.08–4.13 (2H, m), 2.71–2.81 (1H, m), 2.46 (3H, s), 1.04 (9H, s); **LCMS** (Method A): *t*_R_ = 0.61 min, [M + H]^+^, 497, (98% purity); ^**19**^**F NMR** (376 MHz, DMSO-*d*_6_): δ = 48.59 (1F, s); **HRMS** (C_22_H_30_FN_4_O_4_S_2_) [M + H]^+^ requires 497.1693; found [M + H]^+^, 497.1695.

#### (3*R*,5*S*)-1-((*S*)-2-Acetamido-3,3-dimethylbutanoyl)-5-((4-(4-methylthiazol-5-yl)benzyl)carbamoyl)-pyrrolidine-3-sulfonyl fluoride (**VHL-SF1**)

HATU (28 mg, 0.074 mmol) was added to a stirred solution of (3*R*,5*S*)-1-((*S*)-2-amino-3,3-dimethylbutanoyl)-5-((4-(4-methylthiazol-5-yl)benzyl)carbamoyl)pyrrolidine-3-sulfonyl fluoride, 2HCl (28 mg, 0.049 mmol), DIPEA (25.8 μL, 0.147 mmol), and acetic acid (3.09 μL, 0.054 mmol) in DMF (98 μL), and the reaction mixture was stirred at room temperature for 30 min. The reaction mixture was diluted with brine (2 mL) and extracted with DCM (3 × 10 mL). The organic layers were combined and passed through a hydrophobic frit, and the solvent was removed in vacuo. The residue was purified directly by normal-phase column chromatography (0–100% EtOAc in cyclohexane, 12 g SiO_2_, 10 CV) to afford (3*R*,5*S*)-1-((*S*)-2-acetamido-3,3-dimethylbutanoyl)-5-((4-(4-methylthiazol-5-yl)benzyl)carbamoyl)-pyrrolidine-3-sulfonyl fluoride (19 mg, 0.035 mmol, 72% yield) as a white solid. ^**1**^**H NMR** (400 MHz, DMSO-*d*_6_): δ = 8.98 (1H, s), 8.68 (1H, t, *J* = 5.9 Hz), 7.99 (1H, d, *J* = 8.9 Hz), 7.40 (4H, s), 4.90–4.99 (1H, m), 4.61 (1H, t, *J* = 7.6 Hz), 4.45 (2H, d, *J* = 8.9 Hz), 4.41 (1H, d, *J* = 6.4 Hz), 4.22–4.30 (1H, m), 4.13–4.21 (1H, m), 2.64–2.73 (2H, m), 2.44 (3H, s), 1.87 (3H, s), 0.96 (9H, s); ^**13**^**C NMR** (151 MHz, DMSO-*d*_6_): δ = 170.3, 169.6, 169.4, 151.5, 147.7, 139.0, 131.1, 129.8, 128.7 (2C), 127.5 (2C), 59.7 (d, *J* = 14.38 Hz), 58.4, 56.7, 47.8, 41.7, 34.6, 30.1, 26.2 (3C), 22.0, 15.9; ^**19**^**F NMR** (376 MHz, DMSO-*d*_6_): δ = 47.61 (1F, s); **LCMS** (Method A): *t*_R_ = 0.90 min, [M + H]^+^, 539, (100% purity); **HRMS** (C_24_H_31_FN_4_O_5_S_2_) [M + H]^+^ requires 539.1798; found [M + H]^+^, 539.1794.

#### (3*R*,5*S*)-1-((*S*)-2-(*tert*-Butyl)-4,16-dioxo-20-((3a*S*,4*R*,6a*R*)-2-oxohexahydro-1*H*-thieno[3,4-*d*]imidazole-4-yl)-6,9,12-trioxa-3,15-diazaicosanoyl)-5-((4-(4-methylthiazol-5-yl)benzyl)carbamoyl-)pyrrolidine-3-sulfonyl fluoride (**VHL-SF1-Biotin**)

HATU (73.1 mg, 0.192 mmol) was added to a stirred solution of (3*R*,5*S*)-1-((*S*)-2-amino-3,3-dimethylbutanoyl)-5-((4-(4-methylthiazol-5-yl)benzyl)carbamoyl)pyrrolidine-3-sulfonyl fluoride, 2HCl (73 mg, 0.128 mmol), DIPEA (67.2 μL, 0.385 mmol), and 13-oxo-17-((3a*S*,4*R*,6a*R*)-2-oxohexahydro-1*H*-thieno[3,4-*d*]imidazole-4-yl)-3,6,9-trioxa-12-azaheptadecanoic acid (61.1 mg, 0.141 mmol) in DMF (256 μL), and the reaction mixture was stirred at room temperature for 30 min. The reaction mixture was diluted with water (2 mL) and extracted with DCM (3 × 10 mL). The organic layers were combined and passed through a hydrophobic frit, and the solvent was removed in vacuo. The residue was purified by normal-phase column chromatography (0–100% EtOAc in cyclohexane, 24 g SiO_2_, 10 CV, followed by 0–20% MeOH in EtOAc, 5 CV, 20% MeOH in EtOAc, 20 CV) to afford (3*R*,5*S*)-1-((*S*)-2-(*tert*-butyl)-4,16-dioxo-20-((3a*S*,4*R*,6a*R*)-2-oxohexahydro-1*H*-thieno[3,4-*d*]imidazole-4-yl)-6,9,12-trioxa-3,15-diazaicosanoyl)-5-((4-(4-methylthiazol-5-yl)benzyl)carbamoyl)pyrrolidine-3-sulfonyl fluoride (64 mg, 0.070 mmol, 55% yield) as a white foam. ^**1**^**H NMR** (400 MHz, DMSO-*d*_6_): δ = 8.99 (1H, s), 8.72 (1H, t, *J* = 5.9 Hz), 7.80 (1H, br t, *J* = 5.7 Hz), 7.45–7.49 (1H, m), 7.41 (1H, s), 7.38–7.45 (4H, m), 4.93–5.01 (1H, m), 4.64 (1H, t, *J* = 7.6 Hz), 4.56 (1H, d, *J* = 9.4 Hz), 4.42–4.47 (1H, m), 4.40 (1H, d, *J* = 6.4 Hz), 4.26–4.34 (2H, m), 4.16–4.24 (1H, m), 4.10–4.16 (2H, m), 3.97 (2H, s), 3.51–3.63 (9H, m), 3.37–3.41 (2H, m), 3.15–3.20 (2H, m), 3.06–3.12 (1H, m), 2.82 (2H, dd, *J* = 12.3, 5.4 Hz), 2.73 (1H, m), 2.46 (3H, s), 2.06 (2H, t, *J* = 7.4 Hz), 1.43–1.55 (4H, m), 1.25–1.34 (2H, m), 0.97 (9H, s); ^**13**^**C NMR** (151 MHz, DMSO-*d*_6_): δ = 172.1, 170.3, 170.1, 169.1, 169.0, 162.6, 151.5, 147.7, 139.0, 131.0, 129.8, 128.9, 128.7, 128.2, 127.5, 70.3, 69.7, 69.5, 69.5, 69.3, 69.1, 61.0, 59.7, 59.2, 58.3, 55.8, 55.4, 48.1, 41.8, 38.4, 35.0, 30.0, 28.2, 28.0, 26.0 (3C), 25.2, 15.9, 14.1; ^**19**^**F NMR** (376 MHz, DMSO-*d*_6_): δ = 47.79 (1F, s); **LCMS** (Method A): *t*_R_ = 0.88 min, [M + H]^+^, 912, (95% purity); **HRMS** (C_40_H_59_FN_7_O_10_S_3_) [M + H]^+^ requires 912.3470; found [M + H]^+^, 912.3456.

### Synthesis of **VHL-SF2** and **VHL-SF2-Biotin** (Scheme S3)

#### *tert*-Butyl (*S*)-3-(4-Bromophenyl)-3-((*tert*-butoxycarbonyl)-amino)propanoate (**13**)

To a solution of (*S*)-3-(4-bromophenyl)-3-((*tert*-butoxycarbonyl)amino)propanoic acid (50 mg, 145.3 μmol) in toluene (484 μL) was added 1,1-di-*tert*-butoxy-*N*,*N*-dimethyl-methanamine (118.1 mg, 139 μL, 581.1 μmol), and the sealed reaction mixture was heated at 130 °C for 40 min. The reaction mixture was allowed to cool to room temperature and diluted with DCM (25 mL), washed with saturated aqueous sodium bicarbonate (25 mL), and passed through a hydrophobic frit. The solvent was removed in vacuo to afford *tert*-butyl (*S*)-3-(4-bromophenyl)-3-((*tert*-butoxycarbonyl)-amino)propanoate (49 mg, 122.41 μmol, 84%) as a white solid. ^**1**^**H NMR** (400 MHz, DMSO-*d*_6_): δ = 7.51 (2H, d, *J* = 8.4 Hz), 7.44–7.50 (1H, m), 7.26 (2H, d, *J* = 8.4 Hz), 4.78–4.90 (1H, m), 2.55–2.63 (2H, m), 1.28–1.39 (18H, m); ^**13**^**C NMR** (151 MHz, DMSO-*d*_6_): δ = 169.3, 154.7, 142.2, 131.2 (2C), 128.8 (2C), 120.1, 80.1, 78.1, 50.9, 42.2, 28.2 (3C), 27.6 (3C); LCMS (Method A): *t*_R_ = 1.39 min, [M + H]^+^, 401 and 403, (98% purity); **HRMS** (C_18_H_26_BrNO_4_Na) [M + Na]^+^ requires 422.0943; found [M + H]^+^, 422.0943.

#### *tert*-Butyl (*S*)-3-((*tert*-Butoxycarbonyl)amino)-3-(4-(4-methylthiazol-5-yl)phenyl)propanoate (**14**)

A stirred suspension of *tert*-butyl (*S*)-3-(4-bromophenyl)-3-((*tert*-butoxycarbonyl)amino)propanoate (5.815 g, 12.49 mmol), 4-methylthiazole (2.27 mL, 24.99 mmol), PdOAc_2_ (70 mg, 312.31 μmol), potassium carbonate (3.453 g, 24.99 mmol), pivalic acid (423 μL, 3.75 mmol), and DMA (41 mL) were heated at 130 °C for 16 h open to the atmosphere. The reaction mixture was filtered over Celite, diluted with saturated aqueous sodium hydrogen carbonate (100 mL), and extracted with DCM (3 × 100 mL). The organic layers were combined and passed through a hydrophobic frit, and the solvent was concentrated in vacuo. The residue was purified by normal-phase column chromatography (0–50% ethyl acetate in cyclohexane, 330 g SiO_2_, 12 CV) to afford *tert*-butyl (*S*)-3-((*tert*-butoxycarbonyl)amino)-3-(4-(4-methylthiazol-5-yl)phenyl)propanoate (4.498 g, 10.75 mmol, 86%) as a yellow hard gum. ^**1**^**H NMR** (400 MHz, DMSO-*d*_6_): δ = 8.98 (1H, s), 7.52 (1H, br d, *J* = 9.4 Hz), 7.39–7.48 (4H, m), 4.88–4.98 (1H, m), 2.63 (2H, br d, *J* = 7.4 Hz), 2.45 (3H, s), 1.37 (9H, s), 1.34 (9H, s); **LCMS** (Method A): *t*_R_ = 1.27 min, [M + H]^+^, 401 and 403, (93% purity).

#### *tert*-Butyl (*S*)-3-Amino-3-(4-(4-methylthiazol-5-yl)phenyl)propanoate (15)

To a solution of *tert*-butyl (*S*)-3-((*tert*-butoxycarbonyl)amino)-3-(4-(4-methylthiazol-5-yl)phenyl)propanoate (1.34 g, 3.20 mmol) in MeOH (17 mL) was added to 37% HCl (4.3 mL) dropwise, and the reaction mixture was stirred at room temperature for 3 h. The reaction mixture was basified to pH 14 using conc. aqueous NaOH over an ice–water bath and extracted with 10% MeOH in ethyl acetate (3 × 50 mL). The organic layers were combined and passed through a hydrophobic frit, and the solvent was removed in vacuo. The residue was purified by reverse-phase column chromatography (5–65% MeCN in H_2_O + 0.1% NH_4_HCO_3_, 120 g C18, 12 CV) to afford *tert*-butyl (*S*)-3-amino-3-(4-(4-methylthiazol-5-yl)phenyl)propanoate (601 mg, 1.89 mmol, 59%) as a yellow oil. ^**1**^**H NMR** (400 MHz, DMSO-*d*_6_): δ = 8.98 (1H, s), 7.37–7.51 (4H, m), 4.19 (1H, t, *J* = 7.1 Hz), 2.53–2.56 (1H, m), 2.45 (3H, s), 2.03 (2H, br s), 1.33 (9H, s); **LCMS** (Method A): *t*_R_ = 1.03 min, [M + H]^+^, 319, (96% purity).

#### Methyl (2*S*,4*S*)-4-Hydroxypyrrolidine-2-carboxylate (**17**)

4 M HCl in dioxane (40.8 mL, 163 mmol) was added to a solution of 1-(*tert*-butyl) 2-methyl (2*S*,4*S*)-4-hydroxypyrrolidine-1,2-dicarboxylate (10 g, 40.8 mmol) in DCM (163 mL) and MeOH (10 mL); the reaction mixture was stirred at room temperature for 1 h. The solvent was removed in vacuo to afford methyl (2*S*,4*S*)-4-hydroxypyrrolidine-2-carboxylate, HCl (7.387 g, 40.7 mmol, 100% yield) as a white solid. ^**1**^**H NMR** (400 MHz, DMSO-*d*_6_): δ = 9.62 (1H, s), 5.41 (1H, d, *J* = 3.0 Hz), 4.50 (1H, dd, *J* = 9.8, 3.9 Hz), 4.33–4.42 (1H, m), 3.76 (3H, s), 3.19–3.25 (1H, m), 3.13–3.19 (1H, m), 2.27–2.39 (1H, m), 2.08–2.21 (1H, m); ^**13**^**C NMR** (101 MHz, DMSO-*d*_6_): δ = 169.6, 68.1, 57.3, 53.0, 52.9, 37.0.

#### Methyl (2*S*,4*S*)-4-Hydroxy-1-(3-methyl-2-(3-methylisoxazol-5-yl)butanoyl)pyrrolidine-2-carboxylate (**18**)

HATU (12.77 g, 33.6 mmol) was added to a stirred solution of methyl (2*S*,4*S*)-4-hydroxypyrrolidine-2-carboxylate, HCl (4.067 g, 22.39 mmol), DIPEA (11.73 mL, 67.2 mmol), and 3-methyl-2-(3-methylisoxazol-5-yl)butanoic acid (4.10 g, 22.39 mmol) in DCM (45 mL) and DMF (20 mL), and the reaction mixture was stirred at room temperature for 1 h. The reaction mixture was diluted with saturated aqueous sodium hydrogen carbonate (75 mL) and extracted with DCM (3 × 75 mL). The organic layers were combined and passed through a hydrophobic frit, and the solvent was concentrated in vacuo. The residue was purified by reverse-phase column chromatography (5–55% MeCN in H_2_O + 0.1% NH_4_HCO_3_, 130 g C18 (×3), 12 CV) to afford an orange oil. The residue was further purified by normal-phase column chromatography (0–10% MeOH in TBME, 330 g SiO_2_, 10 CV) to afford methyl (2*S*,4*S*)-4-hydroxy-1-(3-methyl-2-(3-methylisoxazol-5-yl)butanoyl)pyrrolidine-2-carboxylate (3.867 g, 12.46 mmol, 56% yield) as light yellow oil. ^**1**^**H NMR** (400 MHz, DMSO-*d*_6_): δ = 6.16–6.26 (1H, m), 5.06–5.17 (1H, m), 4.93–5.03 (1H, m), 4.34–4.44 (1H, m), 4.25–4.33 (1H, m), 4.16–4.25 (1H, m), 3.60–3.68 (3H, m), 3.10–3.19 (1H, m), 2.25–2.37 (2H, m), 2.19–2.23 (3H, m), 1.78–1.86 (1H, m), 1.34 (3H, s), 1.12 (3H, s); **LCMS** (Method A): *t*_R_ = 0.66 min, [M + H]^+^, 311, (97% purity).

#### (2*S*,4*S*)-4-Hydroxy-1-((*R*)-3-methyl-2-(3-methylisoxazol-5-yl)butanoyl)pyrrolidine-2-carboxylic Acid (**19**)

LiOH (0.597 g, 24.92 mmol) was added to a stirred solution of methyl (2*S*,4*S*)-4-hydroxy-1-(3-methyl-2-(3-methylisoxazol-5-yl)butanoyl)pyrrolidine-2-carboxylate (3.867 g, 12.46 mmol) in MeOH (8.31 mL) and water (4.15 mL) over an ice–water bath, and the reaction mixture was stirred for 2 h. The reaction mixture was diluted with water (40 mL) and extracted with EtOAc (3 × 40 mL), and the organic layers were discarded. The aqueous was acidified to pH 2 and extracted with 10:1 EtOAc/MeOH (4 × 50 mL). The organic layers were combined and passed through a hydrophobic frit, and the solvent was removed in vacuo. The enantiomers were separated (Chiralpak IE (250 × 30 mm, 5 μm), 70% Heptane (0.1% formic acid), and 30% ethanol (0.1% formic acid); total flow rate: 40 mL/min. Injection Volume: 400 μL; cycle time: 7.2 min on an Agilent 1200 Prep HPLCUV) to afford (2*S*,4*S*)-4-hydroxy-1-((*R*)-3-methyl-2-(3-methylisoxazol-5-yl)butanoyl)pyrrolidine-2-carboxylic acid (1.025 g, 29% yield) as a white solid. Absolute configuration determined by VCD. ^**1**^**H NMR** (400 MHz, DMSO-*d*_6_): δ = 6.08–6.26 (1H, m), 4.21–4.26 (1H, m), 3.76–3.81 (1H, m), 3.68–3.76 (1H, m), 3.44 (1H, dd, *J* = 10.3, 4.9 Hz), 3.22–3.29 (1H, m), 2.24–2.34 (2H, m), 2.17–2.20 (3H, m), 1.77–1.84 (1H, m), 0.87–1.04 (3H, m), 0.74–0.82 (3H, m); ^**13**^**C NMR** (101 MHz, DMSO-*d*_6_): δ = 172.6, 169.7, 167.7, 159.4, 103.2, 68.5, 57.3, 54.6, 49.0, 37.0, 31.1, 20.4, 19.8, 10.9; **LCMS** (Method A): *t*_R_ = 0.59 min, [M + H]^+^, 297, (98% purity); **HRMS** (C_14_H_21_N_2_O_5_) [M + H]^+^ requires 297.1450; found [M + H]^+^, 297.1455.

#### *tert*-Butyl (*S*)-3-((2*S*,4*S*)-4-Hydroxy-1-((*R*)-3-methyl-2-(3-methylisoxazol-5-yl)butanoyl)pyrrolidine-2-carboxamido)-3-(4-(4-methylthiazol-5-yl)phenyl)propanoate (**20**)

HATU (1.07 g, 2.82 mmol) was added to a stirred solution of (2*S*,4*S*)-4-hydroxy-1-((*R*)-3-methyl-2-(3-methylisoxazol-5-yl)butanoyl)pyrrolidine-2-carboxylic acid (556 mg, 1.88 mmol), DIPEA (654 μL, 3.75 mmol), and *tert*-butyl (*S*)-3-amino-3-(4-(4-methylthiazol-5-yl)phenyl)propanoate (598 mg, 1.88 mmol) in DMF (4 mL), and the reaction mixture was stirred at room temperature for 1 h. The reaction mixture was diluted with water (30 mL) and extracted with DCM (3 × 30 mL). The organic layers were combined and passed through a hydrophobic frit, and the solvent was removed in vacuo. The residue was purified by reverse-phase column chromatography (30–95% MeCN in H_2_O + 0.1% HCOOH, 100 g C18, 12 CV). The volatile solvents were removed in vacuo and aqueous-neutralized to pH 7 using 2 M NaOH. The aqueous was extracted with DCM (3 × 100 mL), the organic layers were combined and passed through a hydrophobic frit, and the solvent was removed in vacuo to afford *tert*-butyl (*S*)-3-((2*S*,4*S*)-4-hydroxy-1-((*R*)-3-methyl-2-(3-methylisoxazol-5-yl)butanoyl)pyrrolidine-2-carboxamido)-3-(4-(4-methylthiazol-5-yl)phenyl)propanoate (846 mg, 1.42 mmol, 76%) as a hard yellow gum. ^**1**^**H NMR** (400 MHz, DMSO-*d*_6_): δ = 8.99 (1H, s), 8.43 (1H, d, *J* = 8.3 Hz), 7.38–7.47 (4H, m), 6.22 (1H, s), 5.26 (1H, br s), 4.26 (1H, dd, *J* = 8.9, 5.3 Hz), 4.16 (1H, br d, *J* = 3.4 Hz), 3.81 (1H, d, *J* = 9.5 Hz), 3.68 (1H, dd, *J* = 10.3, 5.4 Hz), 3.51 (1H, dd, *J* = 10.2, 4.5 Hz), 3.27 (1H, s), 2.74–2.83 (1H, m), 2.63–2.72 (1H, m), 2.45 (3H, s), 2.22–2.32 (2H, m), 2.19 (3H, s), 1.61 (1H, m), 1.31 (9H, s), 0.95 (3H, d, *J* = 6.6 Hz), 0.77 (3H, d, *J* = 6.6 Hz); **LCMS** (Method A): *t*_R_ = 1.09 min, [M + H]^+^, 597, (100% purity).

#### *tert*-Butyl (*S*)-3-((2*S*,4*S*)-1-((*R*)-3-Methyl-2-(3-methylisoxazol-5-yl)butanoyl)-4-((methylsulfonyl)-oxy)pyrrolidine-2-carboxamido)-3-(4-(4-methylthiazol-5-yl)phenyl)propanoate (**21**)

Mesyl chloride (351 μL, 4.50 mmol) was added dropwise to a stirred solution of *tert*-butyl (*S*)-3-((2*S*,4*S*)-4-hydroxy-1-((*R*)-3-methyl-2-(3-methylisoxazol-5-yl)butanoyl)pyrrolidine-2-carboxamido)-3-(4-(4-methylthiazol-5-yl)phenyl)propanoate (2.24 g, 3.75 mmol) and triethylamine (627 μL, 4.50 mmol) in DCM (13 mL) over an ice–water bath, and the reaction mixture was stirred at room temperature for 30 min under a nitrogen atmosphere. The reaction mixture was washed with 5% citric acid (50 mL), followed by water (50 mL), the organic layer was passed through a hydrophobic frit, and the solvent was removed in vacuo. The residue was purified by normal-phase column chromatography (100% cyclohexane, 2 CV, followed by 100% EtOAc, 120 g SiO_2_, 10 CV) to afford *tert*-butyl (*S*)-3-((2*S*,4*S*)-1-((*R*)-3-methyl-2-(3-methylisoxazol-5-yl)butanoyl)-4-((methylsulfonyl)oxy)pyro-lidine-2-carboxamido)-3-(4-(4-methylthiazol-5-yl)phenyl)propanoate (2.237 g, 3.32 mmol, 88%) as a viscous yellow oil. **LCMS** (Method A): *t*_R_ = 1.12 min, [M – H]^+^, 673, (99% purity).

#### *tert*-Butyl (*S*)-3-((2*S*,4*R*)-4-(Acetylthio)-1-((*R*)-3-methyl-2-(3-methylisoxazol-5-yl)butanoyl)pyrro-lidine-2-carboxamido)-3-(4-(4-methylthiazol-5-yl)phenyl)propanoate (**22**)

Thioacetic acid (119.8 μL, 1.67 mmol) was added to a stirred solution of Cs_2_CO_3_ (272 mg, 834.14 μmol) and in DMF (8.5 mL) under a nitrogen atmosphere, and the reaction mixture was stirred at room temperature for 30 min within a sealed vessel. *tert*-Butyl (*S*)-3-((2*S*,4*S*)-1-((*R*)-3-methyl-2-(3-methylisoxazol-5-yl)butanoyl)-4-((methylsulfonyl)oxy)pyrrolidine-2-carboxamido)-3-(4-(4-methylthiazol-5-yl)phenyl)propanoate (866 mg, 1.28 mmol) in DMF (4 mL) was added, and the sealed reaction mixture was stirred at 50 °C for 16 h. The solvent was removed in vacuo, and the residue was purified by reverse-phase column chromatography (40–95% MeCN in H_2_O + 0.1% NH_4_HCO_3_, 130 g C18, 15 CV) to afford *tert*-butyl (*S*)-3-((2*S*,4*R*)-4-(acetylthio)-1-((*R*)-3-methyl-2-(3-methylisoxazol-5-yl)butanoyl)pyrrolidine-2-carboxamido)-3-(4-(4-methylthiazol-5-yl)phenyl)propanoate (368 mg, 561.97 mmol, 44%) as a yellow solid. ^**1**^**H NMR** (400 MHz, DMSO-*d*_6_): δ = 8.99 (1H, s), 8.50 (1H, d, *J* = 8.3 Hz), 7.37–7.47 (4H, m), 6.20 (1H, s), 5.09–5.20 (1H, m), 4.36 (1H, dd, *J* = 8.0, 5.8 Hz), 4.10 (1H, dd, *J* = 10.6, 6.5 Hz), 3.96 (1H, m), 3.79 (1H, d, *J* = 9.8 Hz), 3.46 (1H, dd, *J* = 10.6, 5.5 Hz), 2.64–2.83 (3H, m), 2.45 (3H, s), 2.30 (3H, s), 2.20 (3H, s), 2.01–2.15 (2H, m), 1.33 (9H, s), 0.97 (3H, d, *J* = 6.6 Hz), 0.77 (3H, d, *J* = 6.9 Hz); **LCMS** (Method A): *t*_R_ = 1.22 min, [M – H]^+^, 673, (97% purity).

#### *tert*-Butyl (*S*)-3-((2*S*,4R)-4-(Fluorosulfonyl)-1-((*R*)-3-methyl-2-(3-methylisoxazol-5-yl)butanoyl)pyrr-olidine-2-carboxamido)-3-(4-(4-methylthiazol-5-yl)phenyl)propanoate (**VHL-SF2**)

*tert*-Butyl (*S*)-3-((2*S*,4*R*)-4-(Acetylthio)-1-((*R*)-3-methyl-2-(3-methylisoxazol-5-yl)butanoyl)pyrrolidine-2-carboxamido)-3-(4-(4-methylthiazol-5-yl)phenyl)propanoate (793 mg, 908.24 μmol) and selectfluor (2.413 g, 6.81 mmol) were added to acetonitrile (8.256 mL) and water (825.67 μL), and the reaction mixture was sealed within a vessel and heated within a Biotage microwave for 20 min at 60 °C using a high absorption setting. The reaction mixture was cooled to room temperature. The reaction mixture was diluted with brine (10 mL), extracted with DCM (3 × 10 mL), and passed through a hydrophobic frit, and the solvent was removed in vacuo. The residue was purified by reverse-phase column chromatography (40–95% MeCN +0.1% HCO_2_H in H_2_O + 0.1% HCO_2_H, 100 g C18, 15 CV). The volatiles were removed in vacuo, and the aqueous solution was neutralized with saturated aqueous sodium hydrogen carbonate and extracted with DCM (3 × 100 mL). The organic layers were combined and passed through a hydrophobic frit, and the solvent was removed in vacuo to afford *tert*-butyl (*S*)-3-((2*S*,4R)-4-(fluorosulfonyl)-1-((*R*)-3-methyl-2-(3-methylisoxazol-5-yl)butanoyl)pyrrolidine-2-carboxamido)-3-(4-(4-methylthiazol-5-yl)phenyl)propanoate (268 mg, 404.35 μmol, 45%) as a yellow foam. ^**1**^**H NMR** (400 MHz, DMSO-*d*_6_): δ = 8.99 (1H, s), 8.66 (1H, d, *J* = 8.1 Hz), 7.39–7.53 (4H, m), 6.21 (1H, s), 5.75 (1H, s), 5.08–5.19 (1H, m), 4.81–4.94 (1H, m), 4.50–4.58 (1H, m), 4.15–4.23 (1H, m), 3.96 (1H, d, *J* = 9.5 Hz), 2.66–2.85 (3H, m), 2.46 (3H, s), 2.22–2.34 (2H, m), 2.20 (3H, s), 1.34 (9H, s), 0.91–1.00 (3H, m), 0.80 (3H, d, *J* = 6.6 Hz); ^**13**^**C NMR** (101 MHz, DMSO-*d*_6_): δ = 169.7, 169.6, 169.6, 169.5, 168.0, 159.8, 152.0, 148.4, 141.9, 131.4, 130.8, 129.3 (2C), 127.6 (2C), 103.8, 80.8, 55.3, 50.2, 49.2, 48.2, 42.2, 31.4, 30.5, 28.1 (3C), 21.0, 20.2, 16.4, 11.4; ^**19**^**F NMR** (376 MHz, DMSO-*d*_6_): δ = 47.75 (1F, s); **LCMS** (Method A): *t*_R_ = 1.24 min, [M + Na]^+^ 685, (100% purity); **HRMS** (C_31_H_40_FN_4_O_7_S_2_) [M + H]^+^ requires 663.2322; found [M + H]^+^, 663.2311.

#### (*S*)-3-((2*S*,4*R*)-4-(Fluorosulfonyl)-1-((*R*)-3-methyl-2-(3-methylisoxazol-5-yl)butanoyl)pyrrolidine-2-carboxamido)-3-(4-(4-methylthiazol-5-yl)phenyl)propanoic Acid, 2TFA (**23**)

*tert*-Butyl (*S*)-3-((2*S*,4*R*)-4-(fluorosulfonyl)-1-((*R*)-3-methyl-2-(3-methylisoxazol-5-yl)butanoyl)pyrro-lidine-2-carboxamido)-3-(4-(4-methylthiazol-5-yl)phenyl)propanoate (50 mg, 75.44 μmol) in DCM (150.88 μL) was added to TFA (75 μL, 973.5 μmol), and the reaction mixture was stirred at room temperature for 30 min. The solvent was removed in vacuo to afford (*S*)-3-((2*S*,4*R*)-4-(fluorosulfonyl)-1-((*R*)-3-methyl-2-(3-methylisoxazol-5-yl)butanoyl)pyrrolidine-2-carboxamido)-3-(4-(4-methylthiazol-5-yl)phenyl)propanoic acid, 2TFA (62 mg, 74.28 μmol, 99%) as an orange gum. ^**1**^**H NMR** (400 MHz, DMSO-*d*_6_): δ = 8.97–9.04 (1H, m), 7.86–7.94 (1H, m), 7.37–7.54 (4H, m), 6.22 (1H, s), 5.12–5.21 (1H, m), 4.47–4.57 (1H, m), 4.17–4.23 (1H, m), 3.96 (1H, d, *J* = 9.5 Hz), 2.77–2.86 (3H, m), 2.66–2.75 (3H, m), 2.32–2.35 (1H, m), 2.25–2.31 (1H, m), 2.20 (3H, s), 1.72–1.83 (3H, m), 0.92–1.03 (3H, m), 0.76–0.84 (3H, m); ^**19**^**F NMR** (376 MHz, DMSO-*d*_6_): δ = 47.80 (1F, s); **LCMS** (Method A): *t*_R_ = 0.77 min, [M + H]^+^, 607, (89% purity).

#### (3*R*,5*S*)-1-((*R*)-3-Methyl-2-(3-methylisoxazol-5-yl)butanoyl)-5-(((*S*)-1-(4-(4-methylthiazol-5-yl)phenyl)-3,17-dioxo-21-((3a*S*,4*R*,6a*R*)-2-oxohexahydro-1*H*-thieno[3,4-*d*]imidazole-4-yl)-7,10,13-trioxa-4,16-diazahenicosyl)carbamoyl)pyrrolidine-3-sulfonyl fluoride (**VHL-SF2-Biotin**)

To a stirred solution of (*S*)-3-((2*S*,4*R*)-4-(fluorosulfonyl)-1-((*R*)-3-methyl-2-(3-methylisoxazol-5-yl)butanoyl)pyrrolidine-2-carboxamido)-3-(4-(4-methylthiazol-5-yl)phenyl)propanoic acid, 2TFA (60 mg, 35.94 μmol) and *N*-(2-(2-(2-(2-aminoethoxy)ethoxy)ethoxy)ethyl)-5-((3a*S*,4*R*,6a*R*)-2-oxohexahydro-1*H*-thieno[3,4-*d*]imidazole-4-yl)pentanamide, HCl (18 mg, 39.53 μmol) in DMF (72 μL) and DIPEA (18.8 μL, 107.82 μmol) was added HATU (21 mg, 53.91 μmol), and the reaction mixture was stirred at room temperature for 30 min. The reaction mixture was purified directly by MDAP (formic) to afford (3*R*,5*S*)-1-((*R*)-3-methyl-2-(3-methylisoxazol-5-yl)butanoyl)-5-(((*S*)-1-(4-(4-methylthiazol-5-yl)phenyl)-3,17-dioxo-21-((3a*S*,4*R*,6a*R*)-2-oxohexahydro-1*H*-thieno[3,4-*d*]imidazole-4-yl)-7,10,13-trioxa-4,16-diazahenicosyl)carbamoyl)pyrrolidine-3-sulfonyl fluoride (9 mg, 8.94 μmol, 25%) as a yellow solid. ^**1**^**H NMR** (400 MHz, DMSO-*d*_6_): δ = 8.98 (1H, s), 8.63 (1H, br d, *J* = 8.1 Hz), 7.97 (1H, br t, *J* = 5.9 Hz), 7.79 (1H, br t, *J* = 5.8 Hz), 7.40–7.48 (2H, m), 7.34–7.40 (2H, m), 6.31–6.42 (2H, m), 6.21 (1H, s), 5.15–5.24 (1H, m), 4.82–4.95 (2H, m), 4.48–4.56 (1H, m), 4.26–4.34 (1H, m), 4.20 (1H, br d, *J* = 4.9 Hz), 4.09–4.15 (1H, m), 3.96 (1H, br d, *J* = 9.3 Hz), 3.41–3.51 (6H, m), 3.38 (2H, t, *J* = 6.0 Hz), 3.12–3.22 (3H, m), 3.09 (1H, m), 2.81 (1H, dd, *J* = 12.4, 5.3 Hz), 2.65–2.75 (2H, m), 2.54–2.64 (3H, m), 2.42–2.48 (3H, m), 2.23–2.34 (2H, m), 2.15–2.22 (3H, m), 2.06 (2H, t, *J* = 7.3 Hz), 1.55–1.67 (2H, m), 1.40–1.54 (3H, m), 1.22–1.37 (5H, m), 0.97 (3H, d, *J* = 6.6 Hz), 0.78–0.83 (3H, m); ^**19**^**F NMR** (376 MHz, DMSO-*d*_6_): δ = 47.78 (1F, s); **LCMS** (Method A): *t*_R_ = 0.90 min, [M + 2*H*/2]^+^ 504, (93% purity); **HRMS** (C_45_H_64_FN_8_O_11_S_3_) [M + H]^+^ requires 1007.3841; found [M + H]^+^, 1007.3846.

### Synthesis of **BRD-SF2** (Scheme S4)

#### *tert*-Butyl 4-(1-(1,3-Dimethoxypropan-2-yl)-2-(1,5-dimethyl-6-oxo-1,6-dihydropyridin-3-yl)-1*H*-ben-zo[*d*]imidazole-5-yl)-3,6-dihydropyridine-1(2*H*)-carboxylate (**25**)

5-(5-Bromo-1-(1,3-dimethoxypropan-2-yl)-1*H*-benzo[*d*]imidazole-2-yl)-1,3-dimethylpyridin-2(1*H*)-one (200 mg, 475.84 μmol), *tert*-butyl 4-(4,4,5,5-tetramethyl-1,3,2-dioxaborolan-2-yl)-3,6-dihydropyridine-1(2*H*)-carboxylate (176.6 mg, 571.01 μmol), chloro(2-dicyclohexylphosphino-2′,4′,6′-triisopropyl-1,1′-biphenyl)[2-(2-aminoethyl)phenyl]palladium(II) (28.1 mg, 38.07 μmol), sodium carbonate (151.3 mg, 1.43 mmol) in water (332.8 μL), and 1,4-dioxane (1.331 mL) were sealed within a vessel, and the vessel was evacuated and purged three times with nitrogen. The reaction mixture was heated in a Biotage microwave for 50 min at 100 °C using a normal absorption setting. The reaction mixture was purified directly by MDAP (HpH) to afford *tert*-butyl 4-(1-(1,3-dimethoxypropan-2-yl)-2-(1,5-dimethyl-6-oxo-1,6-dihydropyridin-3-yl)-1*H*-benzo[*d*]imidazole-5-yl)-3,6-dihydropyridine-1(2*H*)-carboxylate (169 mg, 323.35 μmol, 68%) as a white solid. ^**1**^**H NMR** (400 MHz, DMSO-*d*_6_): δ = 8.99 (1H, s), 8.43 (1H, d, *J* = 8.3 Hz), 7.38–7.49 (4H, m), 5.26 (1H, br s), 5.16 (1H, q, *J* = 7.7 Hz), 4.26 (1H, dd, *J* = 8.9, 5.3 Hz), 4.13–4.22 (1H, m), 3.81 (1H, d, *J* = 9.5 Hz), 3.68 (1H, dd, *J* = 10.3, 5.4 Hz), 3.51 (1H, dd, *J* = 10.2, 4.5 Hz), 3.27 (1H, s), 2.75–2.83 (1H, m), 2.64–2.72 (1H, m), 2.45 (3H, s), 2.22–2.32 (2H, m), 2.19 (3H, s), 1.61 (1H, dt, *J* = 12.8, 5.3 Hz), 1.31 (9H, s); **LCMS** (Method B): *t*_R_ = 1.55 min, [M + H]^+^, 523, (99% purity); **HRMS** (C_29_H_39_N_4_O_5_) [M + H]^+^ requires 523.2920; found [M + H]^+^, 523.2919.

#### *tert*-Butyl 4-(1-(1,3-Dimethoxypropan-2-yl)-2-(1,5-dimethyl-6-oxo-1,6-dihydropyridin-3-yl)-1*H*-benzo[*d*]imidazole-5-yl)piperidine-1-carboxylate (**26**)

To a COware tube, 10% Pd/C (34 mg, 31.95 μmol) and *tert*-butyl 4-(1-(1,3-dimethoxypropan-2-yl)-2-(1,5-dimethyl-6-oxo-1,6-dihydropyridin-3-yl)-1*H*-benzo[*d*]imidazole-5-yl)-3,6-dihydropyridine-1(2*H*)-carboxylate (167 mg, 319.53 μmol) in ethanol (3.20 mL) were added under nitrogen, and the tube was sealed. 2 M aqueous HCl (639 μL, 1.28 mmol) and zinc (209 mg, 3.20 mmol) were added under nitrogen to the other tube, which was subsequently sealed. The reaction mixture was stirred at room temperature for 48 h. The reaction mixture was filtered over Celite and washed with DCM (20 mL). The solvent was removed in vacuo, and the sample was purified by MDAP (HpH) to afford *tert*-butyl 4-(1-(1,3-dimethoxypropan-2-yl)-2-(1,5-dimethyl-6-oxo-1,6-dihydropyridin-3-yl)-1*H*-benzo[*d*]imidazole-5-yl)piperidine-1-carboxylate (95 mg, 181.07 μmol, 57%) as a white solid. ^**1**^**H NMR** (400 MHz, DMSO-*d*_6_): δ = 8.01 (1H, d, *J* = 2.2 Hz), 7.69 (1H, d, *J* = 8.3 Hz), 7.65 (1H, dd, *J* = 2.5, 1.2 Hz), 7.46 (2H, d, *J* = 1.5 Hz), 7.11 (1H, dd, *J* = 8.6, 1.5 Hz), 4.81 (1H, tt, *J* = 8.8, 4.4 Hz), 4.10 (2H, br d, *J* = 12.2 Hz), 3.96–4.05 (2H, m), 3.75 (2H, dd, *J* = 10.5, 4.7 Hz), 3.54 (3H, s), 3.16 (6H, s), 2.76–2.85 (2H, m), 2.09 (3H, s), 1.80 (1H, s), 1.81 (2H, br d, *J* = 12.5 Hz), 1.49–1.62 (2H, m), 1.43 (9H, s); **LCMS** (Method B): *t*_R_ = 1.16 min, [M + H]^+^, 525, (100% purity); **HRMS** (C_29_H_41_N_4_O_5_) [M + H]^+^ requires 525.3077; found [M + H]^+^, 525.3080.

#### 5-(1-(1,3-Dimethoxypropan-2-yl)-5-(piperidin-4-yl)-1*H*-benzo[*d*]imidazole-2-yl)-1,3-dimethylpyridin-2(1*H*)-one, 2HCl (**27**)

To a stirred solution of *tert*-butyl 4-(1-(1,3-dimethoxypropan-2-yl)-2-(1,5-dimethyl-6-oxo-1,6-dihydropyridin-3-yl)-1*H*-benzo[*d*]imidazole-5-yl)piperidine-1-carboxylate (93 mg, 177.26 μmol) in 1,4-dioxane (354.5 μL) was added 4 M HCl in dioxane (1.11 mL, 4.43 mmol), and the reaction mixture was stirred at room temperature for 1 h. The solvent was removed in vacuo to afford 5-(1-(1,3-dimethoxypropan-2-yl)-5-(piperidin-4-yl)-1*H*-benzo[*d*]imidazole-2-yl)-1,3-dimethylpyridin-2(1*H*)-one, 2HCl (88 mg, 172.05 μmol, 97%) as a beige solid. ^**1**^**H NMR** (400 MHz, DMSO-*d*_6_): δ = 8.84–9.09 (2H, m), 8.26 (1H, br s), 8.06 (1H, br d, *J* = 8.3 Hz), 7.71 (1H, dd, *J* = 2.5, 1.0 Hz), 7.60 (1H, s), 7.36 (1H, br d, *J* = 8.6 Hz), 4.97–5.11 (1H, m), 4.09 (3H, br dd, *J* = 10.5, 9.1 Hz), 3.82 (3H, br dd, *J* = 10.6, 4.3 Hz), 3.36–3.46 (2H, m), 3.13–3.27 (6H, m), 2.95–3.12 (3H, m), 2.12 (3H, s), 1.95–2.05 (3H, m); **LCMS** (Method B): *t*_R_ = 0.76 min, [M + H]^+^, 425, (100% purity); **HRMS** (C_24_H_32_N_4_O_3_) [M + H]^+^ requires 425.2553; found [M + H]^+^, 425.2557.

#### *tert*-Butyl (2-(2-(2-(2-(4-(1-(1,3-Dimethoxypropan-2-yl)-2-(1,5-dimethyl-6-oxo-1,6-dihydropyridin-3-yl)-1*H*-benzo[*d*]imidazole-5-yl)piperidin-1-yl)-2-oxoethoxy)ethoxy)ethoxy)ethyl)carbamate (**28**)

To a stirred solution of 5-(1-(1,3-dimethoxypropan-2-yl)-5-(piperidin-4-yl)-1*H*-benzo[*d*]imidazole-2-yl)-1,3-dimethylpyridin-2(1*H*)-one, 2HCl (86 mg, 172.87 μmol) and 2,2-dimethyl-4-oxo-3,8,11,14-tetraoxa-5-azahexadecan-16-oic acid (64 mg, 207.45 μmol) in DMF (346 μL) and DIPEA (60.2 μL, 345.75 μmol) was added HATU (132 mg, 345.75 μmol), and the reaction mixture was stirred at room temperature for 30 min. The reaction mixture was purified directly by MDAP (HpH) to afford *tert*-butyl (2-(2-(2-(2-(4-(1-(1,3-dimethoxypropan-2-yl)-2-(1,5-dimethyl-6-oxo-1,6-dihydropyridin-3-yl)-1*H*-benzo[*d*]imidazole-5-yl)piperidin-1-yl)-2-oxoethoxy)ethoxy)ethoxy)ethyl)carbamate (92 mg, 128.88 μmol, 75%) as a white foam. ^**1**^**H NMR** (400 MHz, DMSO-*d*_6_): δ = 8.01 (1H, d, *J* = 2.2 Hz), 7.69 (1H, d, *J* = 8.6 Hz), 7.65 (1H, dd, *J* = 2.5, 1.0 Hz), 7.46 (1H, d, *J* = 1.2 Hz), 7.10 (1H, dd, *J* = 8.6, 1.7 Hz), 6.68–6.76 (1H, m), 4.75–4.76 (1H, m), 4.11–4.27 (2H, m), 3.95–4.02 (3H, m), 3.75 (2H, dd, *J* = 10.3, 4.7 Hz), 3.55–3.61 (4H, m), 3.47–3.55 (7H, m), 3.37 (2H, t, *J* = 6.1 Hz), 3.16 (6H, s), 3.03–3.08 (2H, m), 2.81–2.93 (2H, m), 2.62–2.71 (2H, m), 2.09 (3H, s), 1.79–1.89 (2H, m), 1.60–1.73 (1H, m), 1.45–1.58 (1H, m), 1.37 (9H, s); **LCMS** (Method A): *t*_R_ = 0.97 min, [M + H]^+^, 714 (100% purity).

#### 5-(5-(1-(2-(2-(2-(2-Aminoethoxy)ethoxy)ethoxy)acetyl)piperidin-4-yl)-1-(1,3-dimethoxypropan-2-yl)-1*H*-benzo[*d*]imidazole-2-yl)-1,3-dimethylpyridin-2(1*H*)-one, 2HCl (**29**)

To a stirred solution of *tert*-butyl (2-(2-(2-(2-(4-(1-(1,3-dimethoxypropan-2-yl)-2-(1,5-dimethyl-6-oxo-1,6-dihydropyridin-3-yl)-1*H*-benzo[*d*]imidazole-5-yl)piperidin-1-yl)-2-oxoethoxy)ethoxy)ethoxy)ethyl)carbamate (90 mg, 126.07 μmol) in 1,4-dioxane (252 μL) was added 4 M HCl in dioxane (787.97 μL, 3.1518 mmol), and the reaction mixture was stirred at room temperature for 30 min. The solvent was removed in vacuo to afford 5-(5-(1-(2-(2-(2-(2-aminoethoxy)ethoxy)ethoxy)acetyl)piperidin-4-yl)-1-(1,3-dimethoxypropan-2-yl)-1*H*-benzo[*d*]imidazole-2-yl)-1,3-dimethylpyridin-2(1*H*)-one, 2HCl (86 mg, 125.24 μmol, 99%) as a white foam. **LCMS** (Method A): *t*_R_ = 0.52 min, [M + H]^+^, 614 (97% purity).

#### (3*R*,5*S*)-5-(((*S*)-1-(4-(1-(1,3-Dimethoxypropan-2-yl)-2-(1,5-dimethyl-6-oxo-1,6-dihydropyridin-3-yl)-1*H*-benzo[*d*]imidazole-5-yl)piperidin-1-yl)-15-(4-(4-methylthiazol-5-yl)phenyl)-1,13-dioxo-3,6,9-trioxa-12-azapentadecan-15-yl)carbamoyl)-1-((*R*)-3-methyl-2-(3-methylisoxazol-5-yl)butanoyl)pyrrolidine-3-sulfonyl fluoride (**BRD-SF2**)

To a stirred solution of (*S*)-3-((2*S*,4*R*)-4-(fluorosulfonyl)-1-((*R*)-3-methyl-2-(3-methylisoxazol-5-yl)butanoyl)pyrrolidine-2-carboxamido)-3-(4-(4-methylthiazol-5-yl)phenyl)propanoic acid, 2TFA (50 mg, 59.9 μmol) and 5-(5-(1-(2-(2-(2-(2-aminoethoxy)ethoxy)ethoxy)acetyl)piperidin-4-yl)-1-(1,3-dimethoxypropan-2-yl)-1*H*-benzo[*d*]imidazole-2-yl)-1,3-dimethylpyridin-2(1*H*)-one, 2HCl (45.245 mg, 65.89 μmol) in DMF (119.8 μL) and DIPEA (52.2 μL, 299.50 μmol) was added HATU (34.2 mg, 89.85 μmol), and the reaction mixture was stirred at room temperature for 20 min. The reaction mixture was purified directly by MDAP (formic). The volatile solvents were removed, and the aqueous was basified to pH 10 with saturated aqueous sodium hydrogen carbonate and extracted with DCM (3 × 50 mL). The organic layers were combined and passed through a hydrophobic frit, and the solvent was removed in vacuo to afford (3*R*,5*S*)-5-(((*S*)-1-(4-(1-(1,3-dimethoxypropan-2-yl)-2-(1,5-dimethyl-6-oxo-1,6-dihydropyridin-3-yl)-1*H*-benzo[*d*]imidazole-5-yl)piperidin-1-yl)-15-(4-(4-methylthiazol-5-yl)phenyl)-1,13-dioxo-3,6,9-trioxa-12-azapentadecan-15-yl)carbamoyl)-1-((*R*)-3-methyl-2-(3-methylisoxazol-5-yl)butanoyl)pyrrolidine-3-sulfonyl fluoride (20 mg, 16.63 μmol, 28%) as a white solid. ^**1**^**H NMR** (400 MHz, DMSO-*d*_6_): δ = 8.93–9.02 (1H, m), 8.64 (1H, d, *J* = 8.1 Hz), 8.01 (1H, d, *J* = 2.5 Hz), 7.97 (1H, br t, *J* = 5.8 Hz), 7.87 (1H, br d, *J* = 8.6 Hz), 7.69 (1H, d, *J* = 8.6 Hz), 7.66 (1H, dd, *J* = 2.3, 1.1 Hz), 7.34–7.48 (4H, m), 7.10 (1H, dd, *J* = 8.6, 1.5 Hz), 6.21 (1H, s), 5.16–5.24 (1H, m), 4.86–4.94 (1H, m), 4.82 (1H, m), 4.47–4.56 (2H, m), 4.10–4.32 (4H, m), 3.89–4.03 (4H, m), 3.76 (2H, dd, *J* = 10.3, 4.7 Hz), 3.55–3.62 (2H, m), 3.54 (3H, s), 3.47–3.51 (2H, m), 3.42–3.46 (2H, m), 3.32 (1H, br s), 3.23–3.29 (1H, m), 3.16 (6H, s), 3.02–3.14 (2H, m), 2.83–2.93 (2H, m), 2.65–2.74 (2H, m), 2.58–2.64 (2H, m), 2.45–2.49 (3H, m), 2.23–2.35 (2H, m), 2.18–2.20 (2H, m), 2.09 (3H, s), 1.81–1.89 (2H, m), 1.63–1.72 (1H, m), 1.48–1.59 (1H, m), 1.22–1.31 (2H, m), 0.90–1.02 (3H, m), 0.80 (3H, d, *J* = 6.9 Hz); ^**13**^**C NMR** (151 MHz, DMSO-*d*_6_): δ = 168.9, 167.6, 166.9, 161.7, 159.3, 151.8, 151.5, 147.8, 143.2, 142.0, 139.5, 138.9, 137.3, 132.1, 131.0, 130.0, 128.8, 128.7, 127.4 (2C), 127.0 (2C), 126.9, 121.3, 116.7, 112.2, 108.0, 103.5, 103.3, 69.7, 69.7, 69.6, 69.6, 69.1 (2C), 59.7, 58.3 (2C), 48.7, 47.7, 41.7, 40.0, 38.5, 37.5, 33.7, 33.2, 32.4, 31.7, 30.9, 30.0, 20.7, 20.5, 20.2, 19.7, 17.0, 16.0, 16.0, 14.1, 10.9; ^**19**^**F NMR** (376 MHz, DMSO-*d*_6_): δ = 47.79 (1F, s); **LCMS** (Method A): *t*_R_ = 0.89 min, [(M + 2H)/2]^+^ 601, (95% purity); **HRMS** (C_59_H_77_FN_9_O_13_S_2_) [M + H]^+^ requires 1202.5034; found [M + H]^+^, 1202.5034.

### Synthesis of **AR-VHL-SF2** and **AR2-VHL-SF2** (Scheme S5)

#### (3*R*,5*S*)-5-(((*S*)-3-(4-((4-(((1*r*,3*r*)-3-(4-Cyano-3-(trifluoromethyl)phenoxy)-2,2,4,4-tetramethyl-cyclobutyl)carbamoyl)phenyl)ethynyl)piperidin-1-yl)-1-(4-(4-methylthiazol-5-yl)phenyl)-3-oxo-propyl)carbamoyl)-1-((*R*)-3-methyl-2-(3-methylisoxazol-5-yl)butanoyl)pyrrolidine-3-sulfonyl fluoride (**AR-VHL-SF2**)

To a stirred solution of (*S*)-3-((2*S*,4*R*)-4-(fluorosulfonyl)-1-((*R*)-3-methyl-2-(3-methylisoxazol-5-yl)butanoyl)pyrrolidine-2-carboxamido)-3-(4-(4-methylthiazol-5-yl)phenyl)propanoic acid, 2TFA (50 mg, 59.9 μmol), *N*-((1*r*,3*r*)-3-(4-cyano-3-(trifluoromethyl)phenoxy)-2,2,4,4-tetramethylcyclobutyl)-4-(piperidin-4-ylethynyl)benzamide, HCl (33.6 mg, 59.9 μmol) in DMF (119 μL) and DIPEA (52.2 μL, 299.50 μmol) was added HATU (34.2 mg, 89.85 μmol), and the reaction mixture was stirred at room temperature for 20 min. The reaction mixture was purified directly by MDAP (formic). The volatile solvents were removed, and the aqueous was neutralized with saturated aqueous sodium hydrogen carbonate and extracted with DCM (3 × 50 mL). The organic layers were combined and passed through a hydrophobic frit, and the solvent was removed in vacuo to afford (3*R*,5*S*)-5-(((*S*)-3-(4-((4-(((1*r*,3*r*)-3-(4-cyano-3-(trifluoromethyl)phenoxy)-2,2,4,4-tetramethylcyclobutyl)carbamoyl)phenyl)ethynyl)piper-idin-1-yl)-1-(4-(4-methylthiazol-5-yl)phenyl)-3-oxopropyl)carbamoyl)-1-((*R*)-3-methyl-2-(3-methyl-isoxazol-5-yl)butanoyl)pyrrolidine-3-sulfonyl fluoride (23 mg, 20.40 μmol, 35%) as a white solid. ^**1**^**H NMR** (400 MHz, DMSO-*d*_6_): δ = 8.92–8.99 (1H, m), 8.60 (1H, br dd, *J* = 7.8, 2.5 Hz), 8.10 (1H, d, *J* = 8.6 Hz), 7.89 (1H, br d, *J* = 8.8 Hz), 7.79–7.85 (2H, m), 7.38–7.50 (6H, m), 7.30 (1H, dd, *J* = 8.6, 2.5 Hz), 6.18–6.23 (1H, m), 5.18–5.29 (1H, m), 4.83–4.98 (2H, m), 4.52–4.57 (1H, m), 4.40 (1H, s), 4.17–4.28 (2H, m), 4.09 (1H, d, *J* = 8.8 Hz), 3.92–4.00 (1H, m), 3.77–3.86 (1H, m), 3.61–3.72 (1H, m), 2.84–2.98 (3H, m), 2.65–2.77 (1H, m), 2.43–2.47 (3H, m), 1.73–1.88 (3H, m), 1.18–1.29 (12H, m), 1.15 (6H, s), 0.95 (4H, m), 0.76–0.84 (4H, m); ^**13**^**C NMR** (151 MHz, DMSO-*d*_6_): δ = 174.4, 169.1, 168.4, 166.6, 161.8, 159.3, 159.2, 137.7, 132.8, 132.6, 131.0, 131.0 (d, *J* = 1.66 Hz), 131.0 (2C), 128.8 (2C), 127.8, 127.8 (2C), 127.8, 127.2, 125.6, 123.2, 121.4, 118.7, 115.7, 114.4 (d, *J* = 4.98 Hz), 103.3, 99.6, 84.0, 58.3, 50.1, 48.7, 48.6, 47.7 (d, *J* = 3.87 Hz), 47.1, 41.8, 40.3 (2C), 33.6, 31.3, 30.9 (2C), 30.0, 29.0, 24.5, 24.0 (2C), 23.1 (2C), 20.5, 16.0, 15.9, 13.9, 11.0, 10.9; ^**19**^**F NMR** (376 MHz, DMSO-*d*_6_): δ = (1F, s); **LCMS** (Method A): *t*_R_ = 1.46 min, [M + H]^+^, 1112, (99% purity); **HRMS** (C_57_H_62_F_4_N_7_O_8_S_2_) [M + H]^+^ requires 1112.4037; found [M + H]^+^, 1112.4027.

#### (3*R*,5*S*)-5-(((*S*)-3-(4-((4-(((1*r*,3*r*)-3-(3-Chloro-4-cyanophenoxy)-2,2,4,4-tetramethylcyclobutyl)-carbamoyl)phenyl)ethynyl)piperidin-1-yl)-1-(4-(4-methylthiazol-5-yl)phenyl)-3-oxopropyl)-carbamoyl)-1-((*R*)-3-methyl-2-(3-methylisoxazol-5-yl)butanoyl)pyrrolidine-3-sulfonyl fluoride (**AR2-VHL-SF2**)

To a stirred solution of (*S*)-3-((2*S*,4*R*)-4-(fluorosulfonyl)-1-((*R*)-3-methyl-2-(3-methylisoxazol-5-yl)butanoyl)pyrrolidine-2-carboxamido)-3-(4-(4-methylthiazol-5-yl)phenyl)propanoic acid, 2TFA (39 mg, 46.72 μmol) and *N*-((1*r*,3*r*)-3-(3-chloro-4-cyanophenoxy)-2,2,4,4-tetramethylcyclobutyl)-4-(piperidin-4-ylethynyl)benzamide, HCl (24.6 mg, 46.72 μmol) in DMF (93 μL) and DIPEA (40.7 μL, 233.61 μmol) was added HATU (26.7 mg, 70.08 μmol), and the reaction mixture was stirred at room temperature for 20 min. The reaction mixture was purified directly by MDAP (formic). The volatiles were removed, and the aqueous was basified with saturated aqueous sodium hydrogen carbonate and extracted with DCM (3 × 50 mL). The organic layers were combined and passed through a hydrophobic frit, and the solvent was removed in vacuo to afford (3*R*,5*S*)-5-(((*S*)-3-(4-((4-(((1*r*,3*r*)-3-(3-chloro-4-cyanophenoxy)-2,2,4,4-tetramethylcyclobutyl)carbamoyl)phenyl)ethynyl)piperidin-1-yl)-1-(4-(4-methylthiazol-5-yl)phenyl)-3-oxopropyl)carbamoyl)-1-((*R*)-3-methyl-2-(3-methylisoxazol-5-yl)butan-oyl)pyrrolidine-3-sulfonyl fluoride (22 mg, 20.40 μmol, 44%) as a white solid. ^**1**^**H NMR** (400 MHz, DMSO-*d*_6_): δ = 8.92–8.99 (1H, m), 8.60 (1H, br dd, *J* = 7.8, 3.42 Hz), 7.90 (1H, d, *J* = 8.8 Hz), 7.87 (1H, br d, *J* = 9.3 Hz), 7.77–7.84 (2H, m), 7.38–7.50 (5H, m), 7.21 (1H, d, *J* = 2.5 Hz), 7.01 (1H, dd, *J* = 8.8, 2.5 Hz), 6.19–6.23 (1H, m), 5.16–5.29 (1H, m), 4.83–4.97 (2H, m), 4.51–4.58 (1H, m), 4.32 (1H, s), 4.25 (1H, m), 4.14–4.22 (1H, m), 4.07 (1H, d, *J* = 9.1 Hz), 3.96 (1H, br d, *J* = 9.5 Hz), 3.77–3.86 (1H, m), 3.61–3.71 (1H, m), 2.85–2.98 (3H, m), 2.65–2.76 (1H, m), 2.43–2.47 (3H, m), 2.24–2.35 (2H, m), 2.19 (3H, d, *J* = 3.4 Hz), 1.73–1.86 (2H, m), 1.35–1.58 (3H, m), 1.23 (6H, s), 1.14 (6H, s), 0.92–1.00 (3H, m), 0.76–0.84 (4H, m); ^**13**^**C NMR** (101 MHz, DMSO-*d*_6_): δ = 169.2, 169.1, 169.1, 169.0, 166.5, 162.5, 159.3, 151.5, 147.8, 136.8 (2C), 136.0, 133.8, 131.0, 131.0 (2C), 128.7 (2C), 127.8 (2C), 127.1, 125.6 (2C), 116.8, 116.2, 114.6, 103.6, 103.3, 83.9, 58.4, 48.7, 48.5, 40.3 (2C), 30.9 (2C), 30.0 (2C), 23.9 (2C), 23.1 (2C), 20.5, 20.2, 19.8, 19.7, 15.9, 15.9, 11.0, 10.9; ^**19**^**F NMR** (376 MHz, DMSO-*d*_6_): δ = 47.83 (1F, s); **LCMS** (Method A): *t*_R_ = 1.14 min, [M + H]^+^, 1078 and 1080, (100% purity); **HRMS** (C_56_H_62_ClFN_7_O_8_S_2_) [M + H]^+^ requires 1078.3774; found [M + H]^+^, 1078.3754.

## Supporting Information Available

Synthetic Schemes; HPLC traces for final compounds; organic synthesis methods; and biological methods (PDF)
Docking results for **VHL-SF1** (PDB)
Docking results for **VHL-SF2** (PDB)
Molecular formula strings (CSV)

## Supplementary Material

jm3c02123_si_001.pdf

jm3c02123_si_002.pdb

jm3c02123_si_003.pdb

## Abbreviations

AR: androgen receptor
BRD4: bromodomain-containing protein 4
BRET: bioluminescence resonance energy transfer
CRBN: cereblon
DCAF: DDB1- and CUL4-associated factor
FP: fluorescence polarization
HIF1α: hypoxia-inducible factor 1-alpha
NanoLuc: nanoluciferase
POI: protein of interest
PROTAC: proteolysis-targeting chimera
RNF: RING Finger proteins
SDS-PAGE: sodium dodecyl sulfate–polyacrylamide gel electrophoresis
TPD: targeted protein degradation
VCB: stable complex of VHL protein with elongin C and elongin B
VHL: Von Hippel-Lindau
