# Supplementary material for: Structure-Guided Design and Optimization of Covalent VHL-Targeted Sulfonyl Fluoride PROTACs
Source: J Med Chem. 2024 Mar 13;67(6):4641–54. doi: 10.1021/acs.jmedchem.3c02123 (PMC10982999; doi:10.1021/acs.jmedchem.3c02123)
Supplement: Supplementary file 1 — jm3c02123_si_001.pdf [file jm3c02123_si_001.pdf]

Supplementary Information

Structure-guided design and optimization of covalent VHL-targeted sulfonyl fluoride PROTACs

Rishi R. Shah,[*a,b] Elena De Vita,[b,c] Preethi S. Sathyamurthi,[a] Daniel Conole,[b] Xinyue Zhang,[b] Elliot Fellows,[a] Eleanor R. Dickinson,[a] Carlos M. Fleites,[a] Markus A. Queisser,[a] John D. Harling,[a] Edward W. Tate[*b,d]

[a] GSK, Medicines Research Centre, Stevenage, Hertfordshire, SG1 2NY, UK

[b] Department of Chemistry, Molecular Sciences Research Hub, Imperial College London, 80 Wood Lane, London W12 0BZ, UK

[c] Department of Biochemistry, School of Biological and Behavioural Sciences, Queen Mary University of London, 327 Mile End Road, E1 4NS, UK

[d] The Francis Crick Institute, 1 Midland Road, London, NW1 1AT, UK

[*] Corresponding Authors: Rishi Shah (rishi.shah@ubiquigent.com) & Ed Tate (e.tate@imperial.ac.uk)

Table of Contents

Supplementary Figures and Tables S2

Synthetic Schemes S8

HPLC traces for final compounds S12

Organic Synthesis Methods S19

Biological Methods S21

References S26

Supplementary Figures and Tables

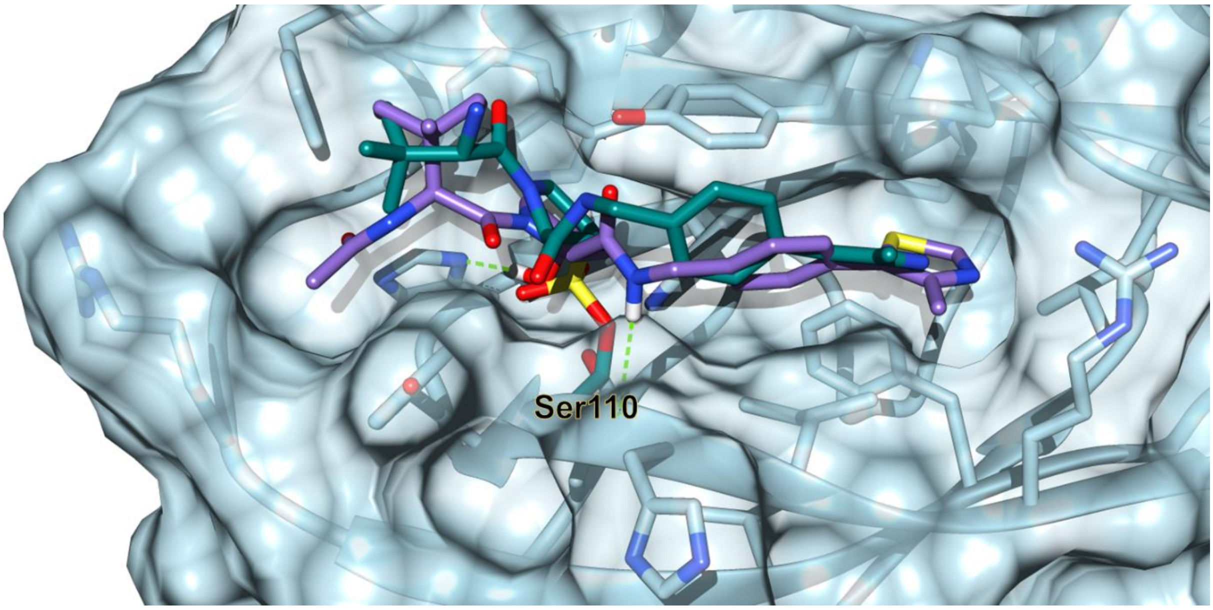

Figure S1. A docking of **VHL-SF1** (blue) within VHL superimposed onto **VH032** (purple). Docking was performed with MOE using the Covalent Docking protocol. Docking made use of PDB: 4W9H as the crystal structure.

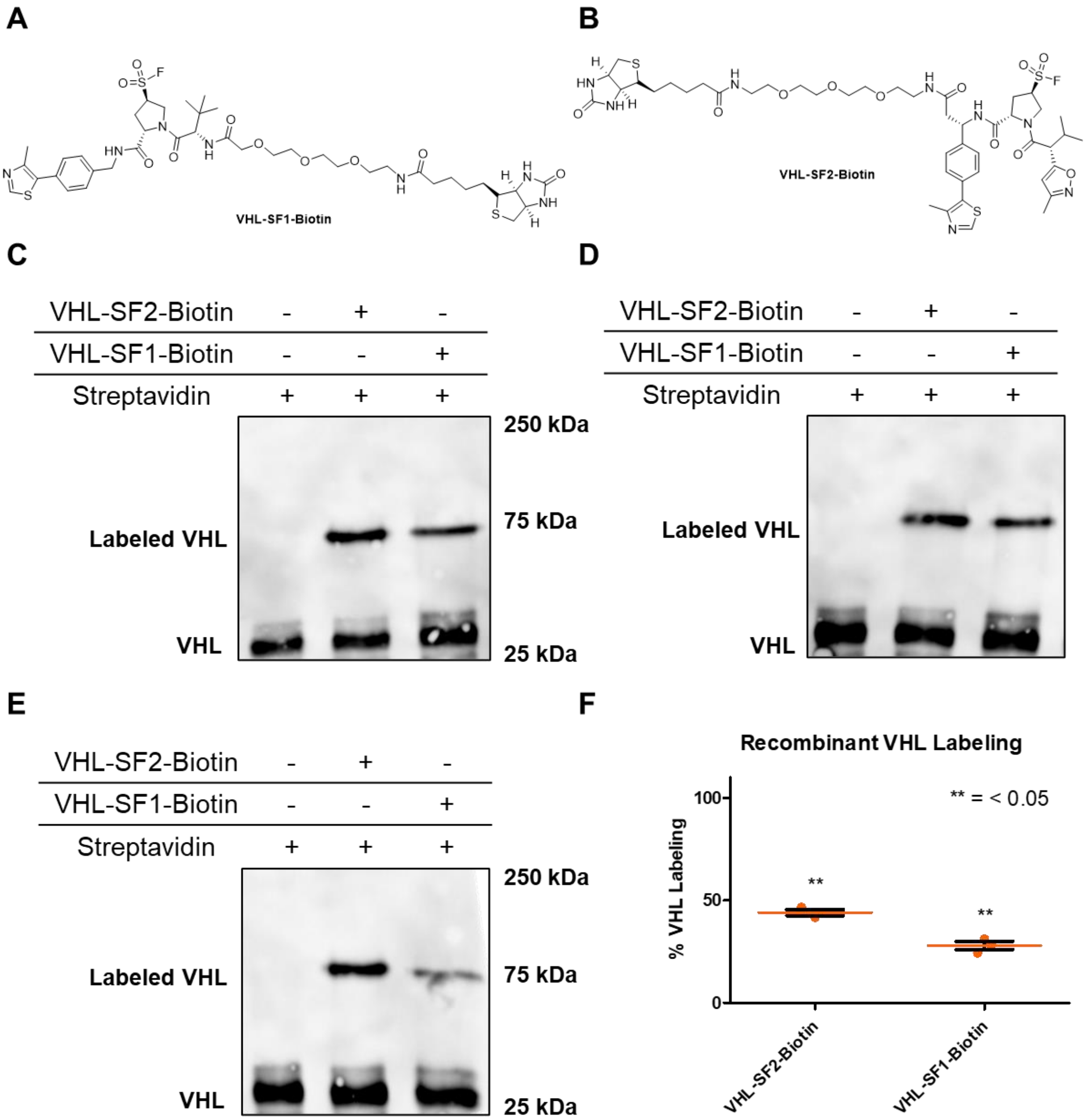

Figure S2. (A) Structure of **VHL-SF-1-Biotin**. (B) Structure of **VHL-SF-2-Biotin**. (C - E) Gel-based streptavidin shift assay of **VHL-SF1-Biotin**, and **VHL-SF2-Biotin**. VCB protein was pre-treated with DMSO, **VHL-SF1-Biotin** (10 μM) or VHL-SF2-Biotin (10 μM) for 2 h at room temperature prior to addition of streptavidin (10 μM, 10 min) at room temperature, after which protein was resolved on SDS-PAGE and visualised by VHL western blotting. (F) Quantification of VHL levels from western blot. Data shows individual quantified western blot ± SEM (n = 3).

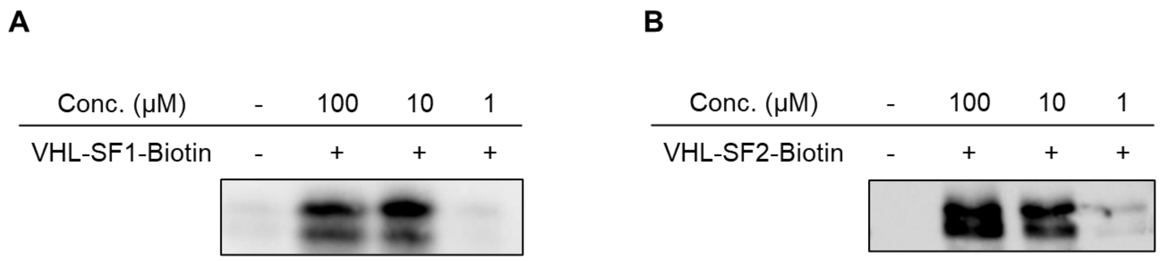

Figure S3. (A) Gel-based VHL labelling of **VHL-SF1-Biotin**, and (B) **VHL-SF2-Biotin**. VCB protein was pre-treated with DMSO, **VHL-SF1-Biotin** or **VHL-SF2-Biotin** for 2 h at room temperature, after which protein was resolved on SDS-PAGE and visualised by Neutravidin HRP western blotting.

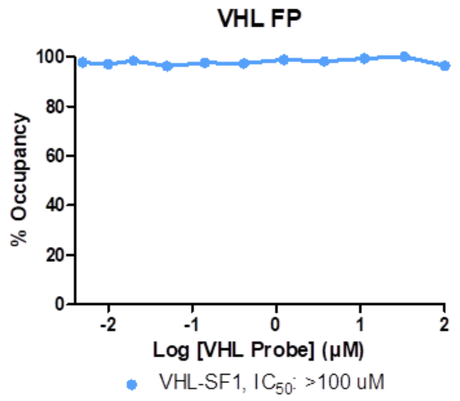

Figure S4. Dose-response of **VHL-SF1** following a 2 h pre-incubation in the presence of VCB and FAM-conjugated HIF1α-derived peptide, assessed by fluorescence polarization. Data shows mean ± SEM (n = 3).

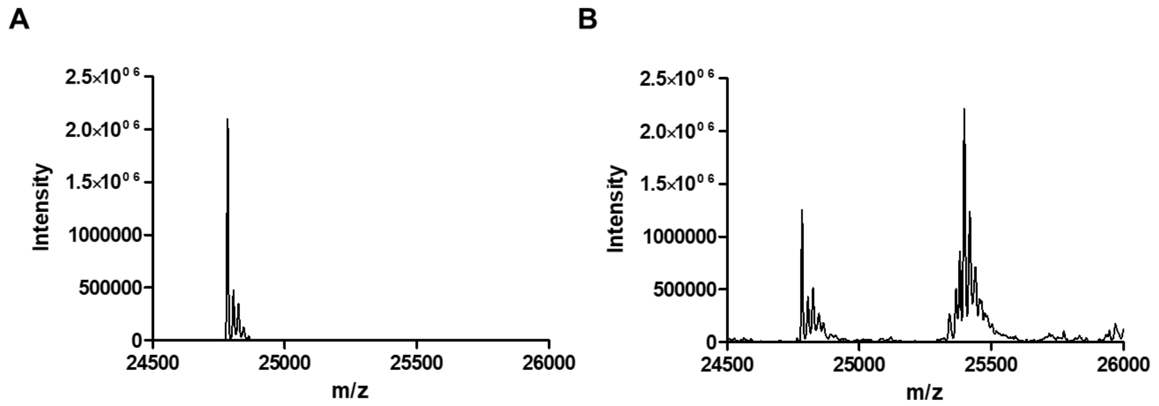

Figure S5. (A) VCB was incubated with DMSO for 24 h and subjected to intact-protein LC-MS (VHL: 24784 Da). (B) VCB was incubated with **VHL-SF2** (100 μM) for 24 h and subjected to intact-protein LC-MS (65% mono-VHL labelling by **VHL-SF2**; VHL: 24783, [M-^{t}Bu+VHL+Na]$^+$ 25397 Da).

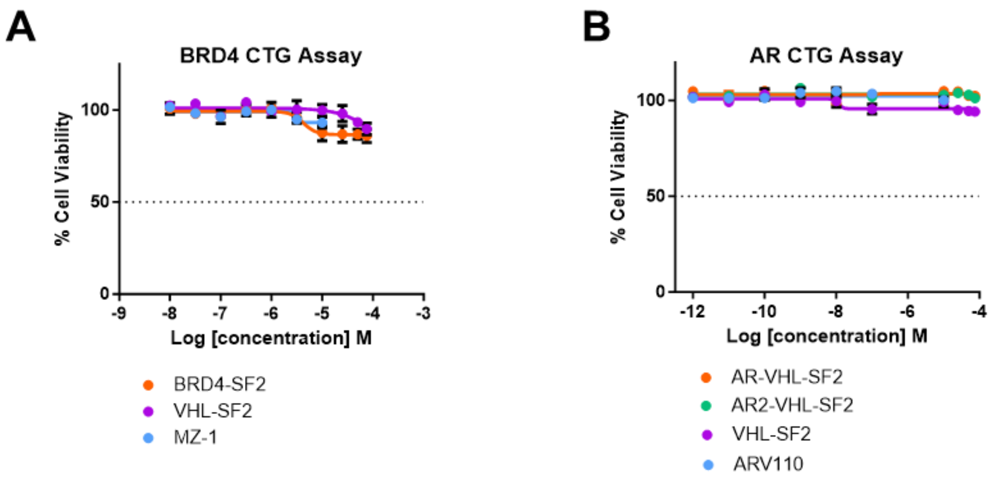

Figure S6. (A) BRD4-HiBiT HEK293T cells were treated with **BRD-SF2**, **MZ-1** or **VHL-SF2** at varying concentrations (70 - 0.01 μM) for 18 h. After the treatment period, The CellTiter-Glo® 2.0 Assay reagents were used for this experiment per the manufacturer's instruction. Data shows mean ± SEM (n = 4). (B) AR-HiBiT LNCaP Cells were treated with **AR-VHL-SF2**, **AR2-VHL-SF2**, **ARV110** or **VHL-SF2** at varying concentrations (75 μM - 1 pM) for 16 h. After the treatment period, The CellTiter-Glo® 2.0 Assay reagents were used for this experiment per the manufacturer's instruction. Data shows mean ± SEM (n = 4).

Table S1. Sulfonyl fluoride warhead stability for **VHL-SF1** and **VHL-SF2** was determined in PBS at 40 °C at the reported pH. **Stability could not be determined due to solubility issues.*

pH	VHL-SF1 $t_{1/2}$ (h)	VHL-SF2 $t_{1/2}$ (h)
7.5	0.55	0.40
6.0	15	15
5.0	ND*	14
4.0	ND*	38

Table S2. Summary of siteID analyses performed to verify binding of VHL-SF2 to recombinant VHL.

Protease	Instrument Analysis	Sequence coverage of VCB (%)	DMSO Ser110-containing Peptide Sequence Identification	VHL-SF2 Treated Ser110-containing Peptide Sequence	Before Digestion Analysis	
					DMSO VHL Intact Mass (Da)	VHL-SF2-Modified VHL Intact Mass (Da)
**Thermolysin**	QExactive	79.38	IH**S**YRGHLWL	ND	24783	25396
**Thermolysin**	Exploris 240	85.63	LNFDGEPQPYPTL PPGTGRRIH**S**	ND	24783	25396
**Pepsin pH2**	QExactive	86.88	ND	ND	24783	25396
**Pepsin pH2**	Exploris 240	80	ND	ND	24783	25396
**Trypsin**	QExactive	84.38	RIH**S**YR	ND	24783	25396
**Trypsin**	Exploris 240	84.38	ND	ND	24783	25396

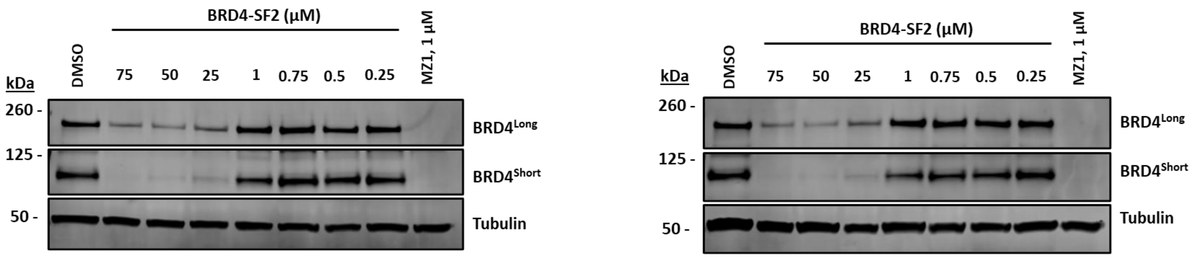

Figure S7. BRD-SF2-mediated degradation of endogenous BRD4. HEK293 cells were treated with DMSO, **MZ-1** or varying concentrations of **BRD-SF2** for 18 h. BRD4 and loading control GAPDH levels were visualized by western blotting. Data shows western blots from n = 3.

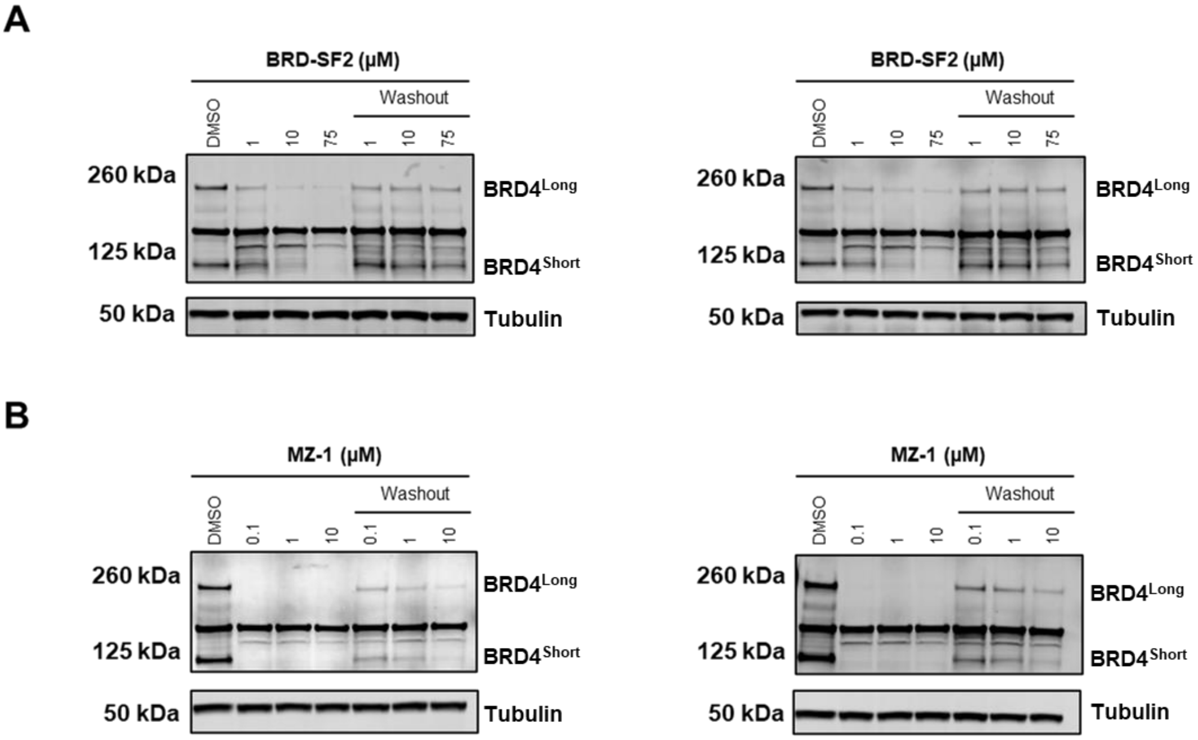

Figure S8. (A) BRD4-HiBiT HEK293 cells were treated with **BRD-SF2** (75 – 1 µM) for 5 h followed by PBS washing and 24 h recovery. BRD4 (top) and loading control Tubulin (bottom) levels were visualized by western blotting. Data shows western blots from n = 2. (B) BRD4-HiBiT HEK293T cells were treated with **MZ-1** (10 – 0.1 µM, n = 2) for 5 h followed by PBS washing and 24 h recovery. BRD4 (top) and loading control Tubulin (bottom) levels were visualized by western blotting. Data shows western blots from n = 2.

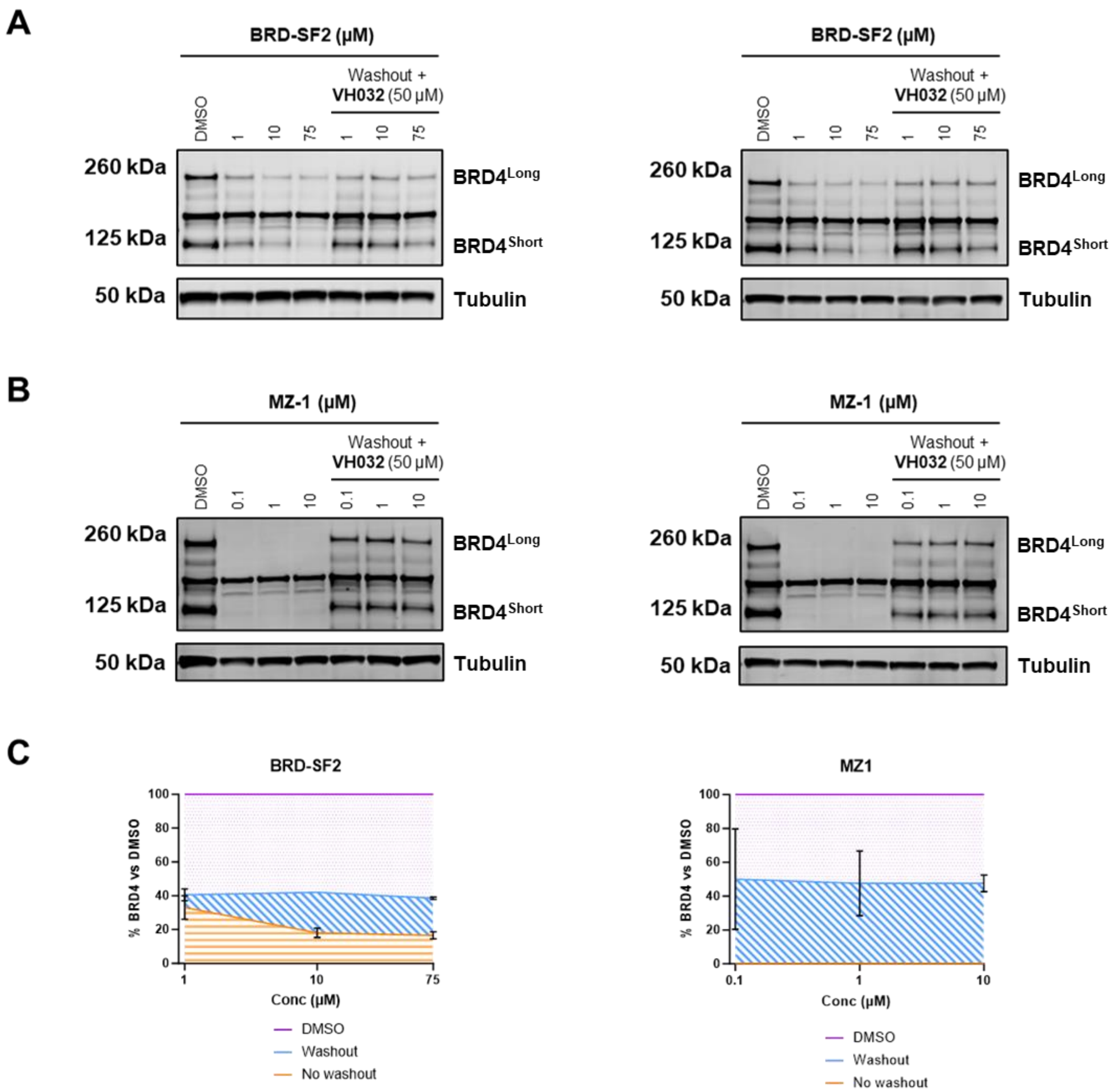

Figure S9. (A,B) Original western blot duplicates used for the quantification of washout experiments presented in Fig. 4A,B for **BRD-SF2** (A) and **MZ-1** (B). BRD4 (top) and loading control Tubulin (bottom) levels were visualized by western blotting. (C) Quantification of residual BRD4 % (long isoform) in washout competition experiments for **BRD-SF2** (left) and **MZ-1** (right) (n = 2). The AUC was used to calculate relative loss in degradation percentages of washout conditions vs standard treatment (29 h).

Synthetic Schemes

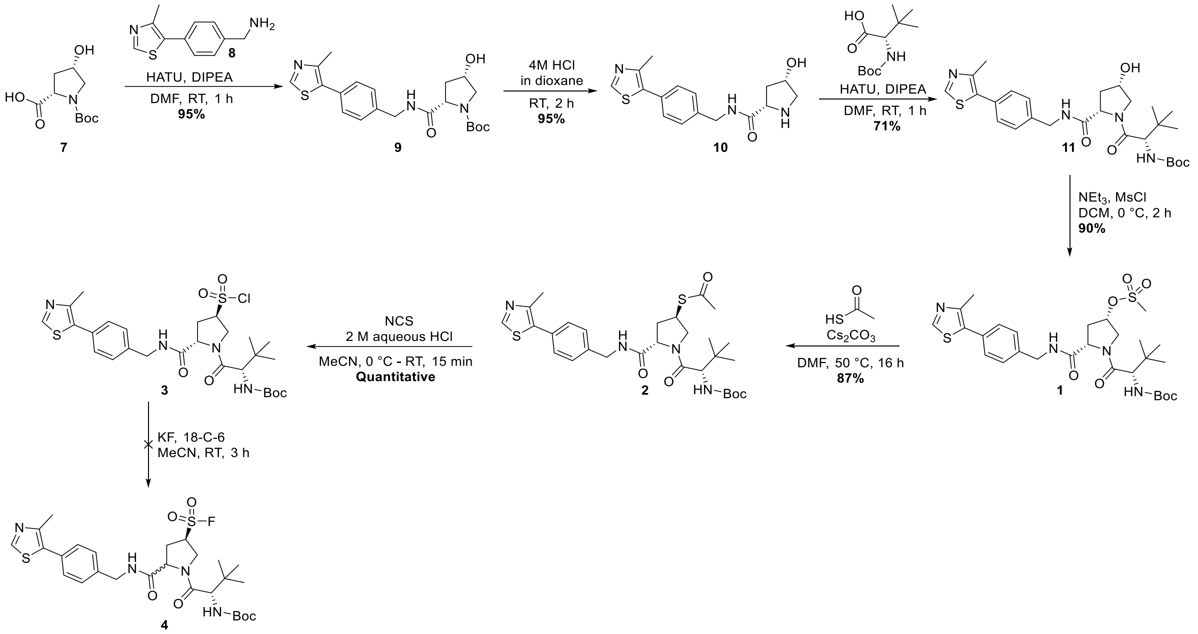

Scheme S1. Synthesis of **VHL-SF1** resulting in epimerization of the proline ring when subjected to KF and 18-C-6.

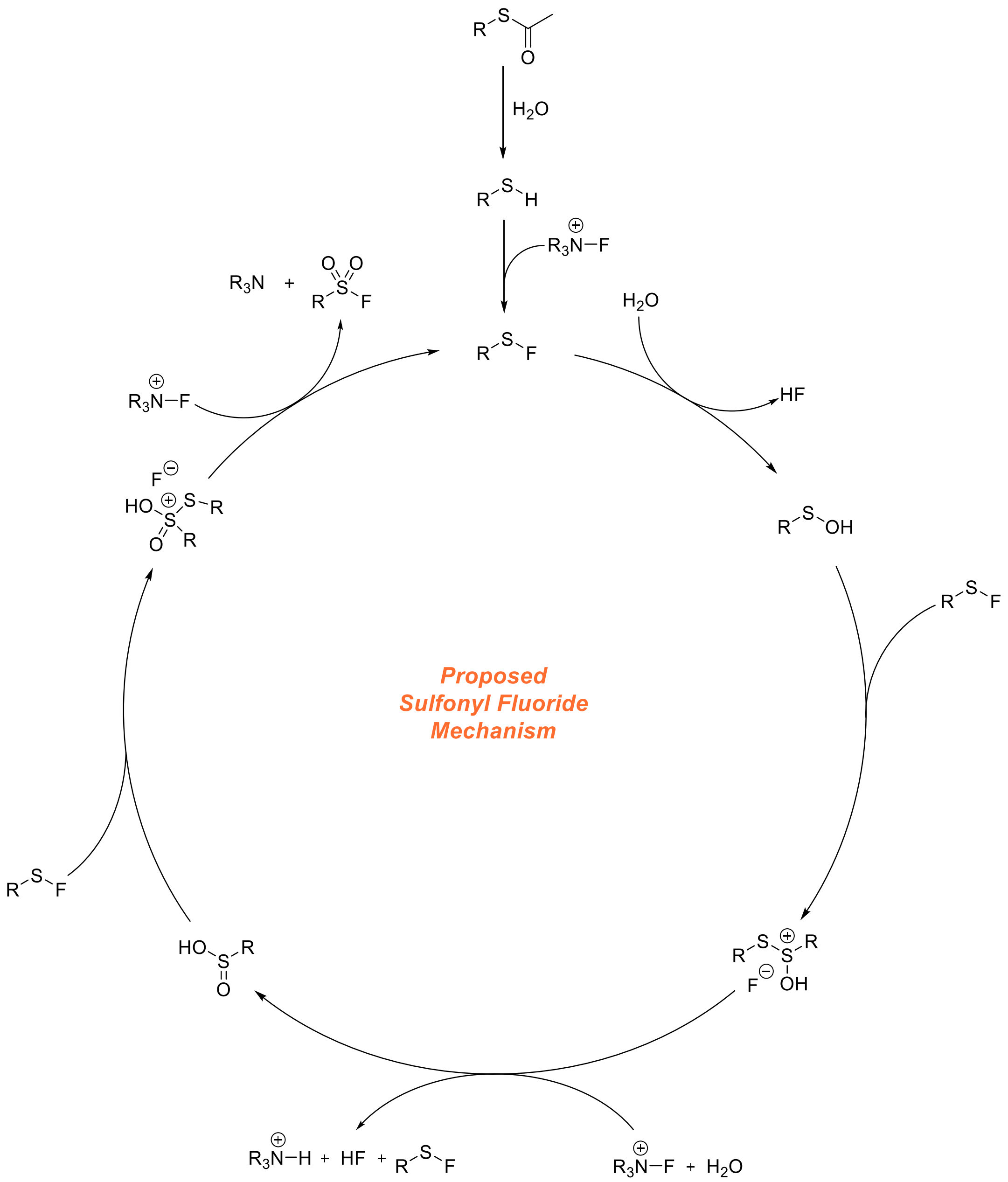

Scheme S2. Proposed mechanism of the novel sulfonyl fluoride reaction, to convert thioacetates to sulfonyl fluorides.

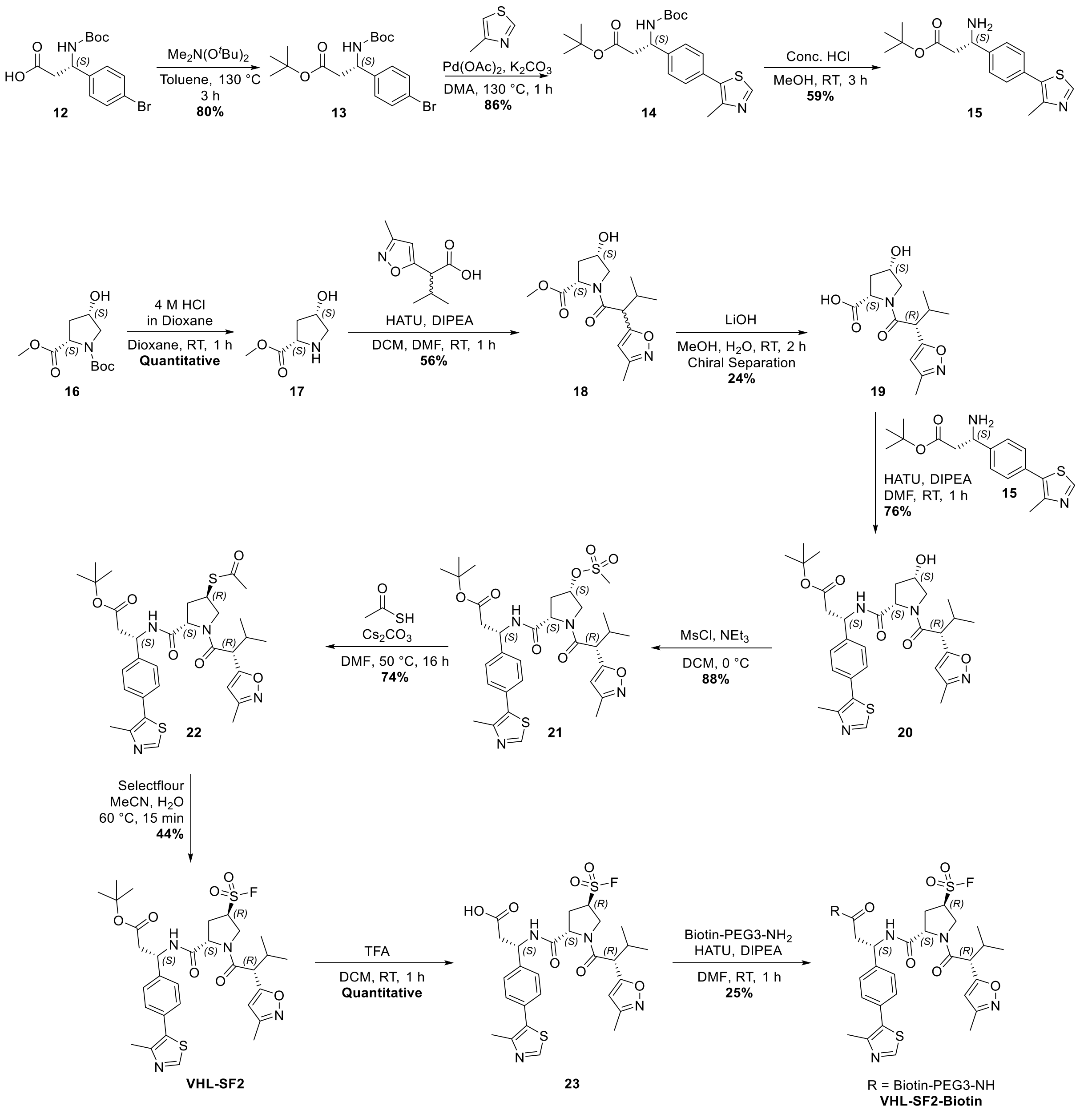

Scheme S3. Synthesis of **VHL-SF2** and **VHL-SF2-Biotin**.

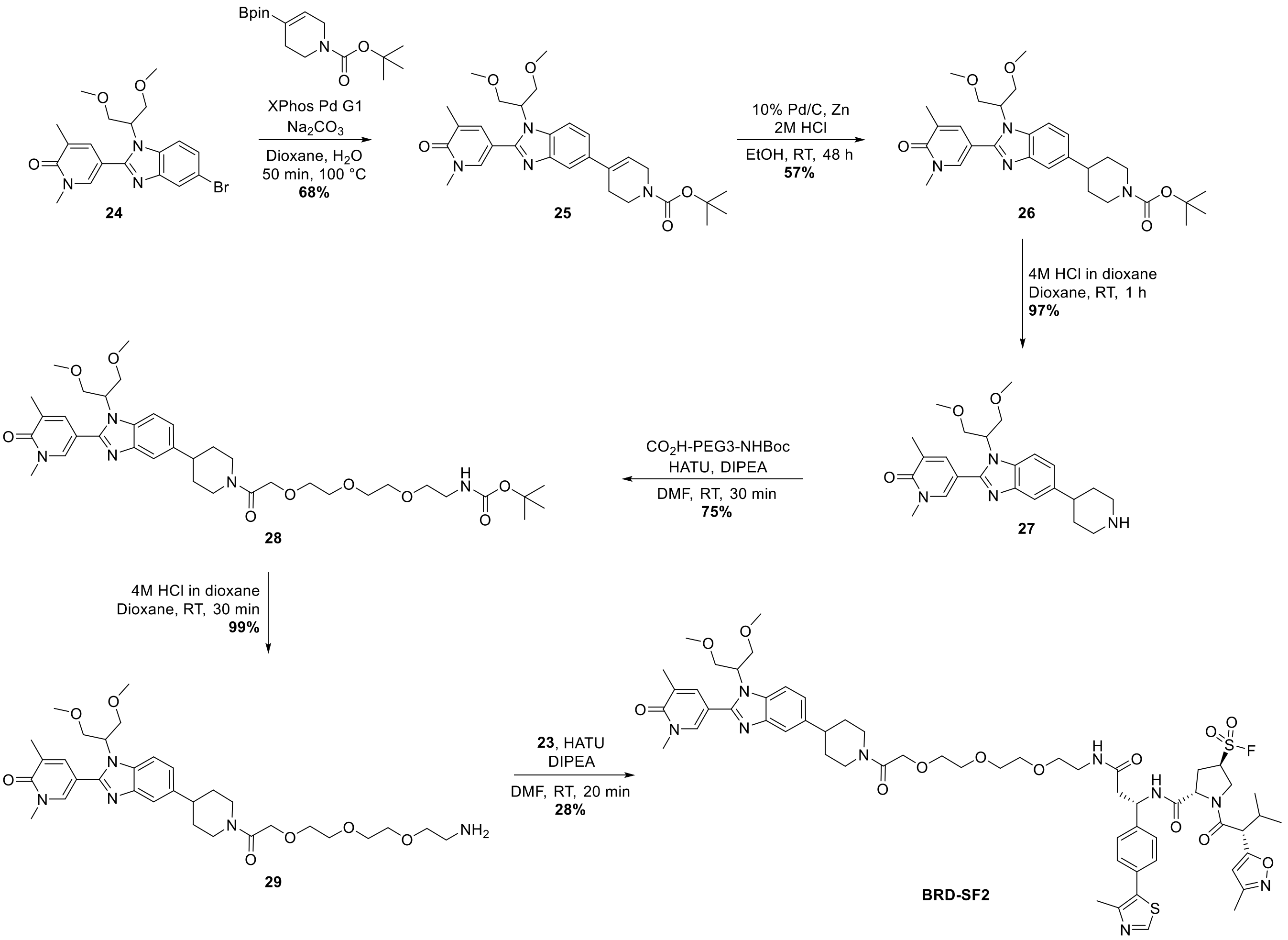

Scheme S4. Synthesis of BRD-SF2. Benzimidazole 24 was synthesized according to literature precedent.[1]

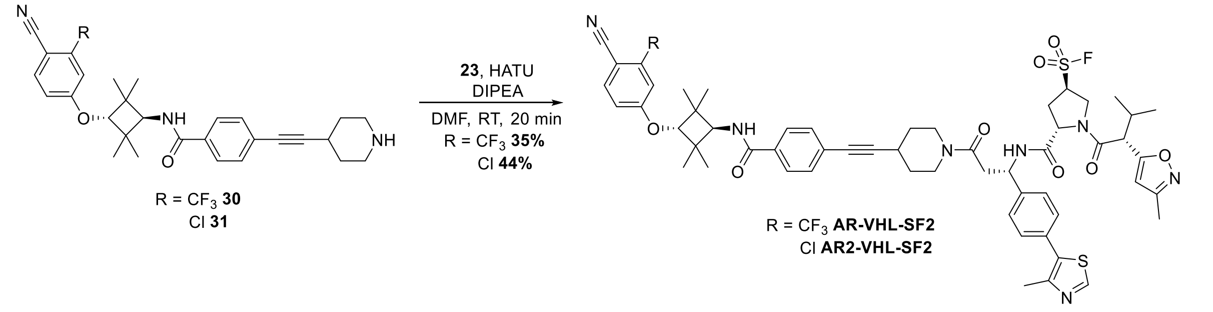

Scheme S5. Synthesis of AR-VHL-SF2 and AR2-VHL-SF2. AR binders **30** and **31** were synthesized according to literature precedent.[2]

HPLC traces for final compounds

- **VHL-SF1**

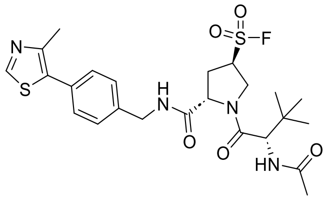

Chemical Formula: $C_{24}H_{31}FN_4O_5S_2$
Exact Mass: 538.17
Molecular Weight: 538.65

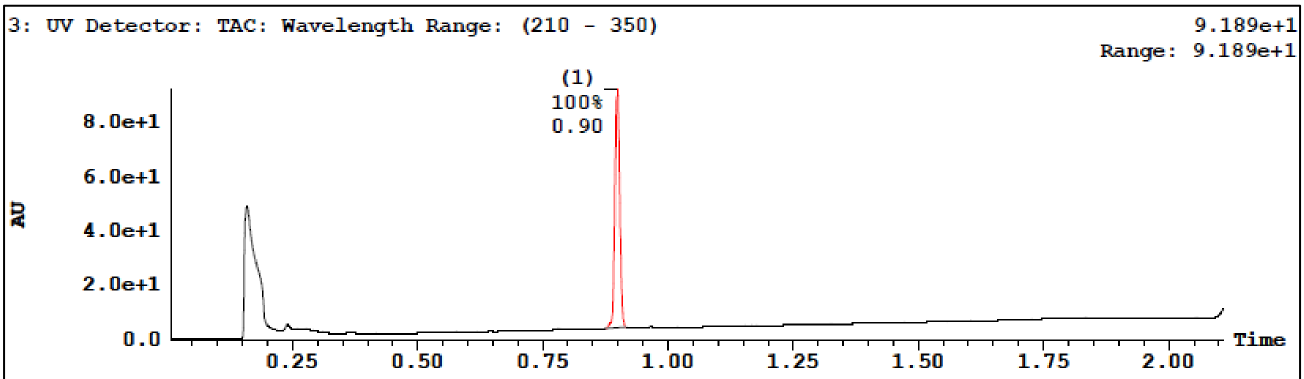

Peak Number	Compound	Time	AreaAbs	Area %Total	Width	Height	Mass Found
1		0.90	1e+006	100.00	0	9e+007	Not Found

Peak ID	Compound	Time	Mass Found
1		0.90	Not Found

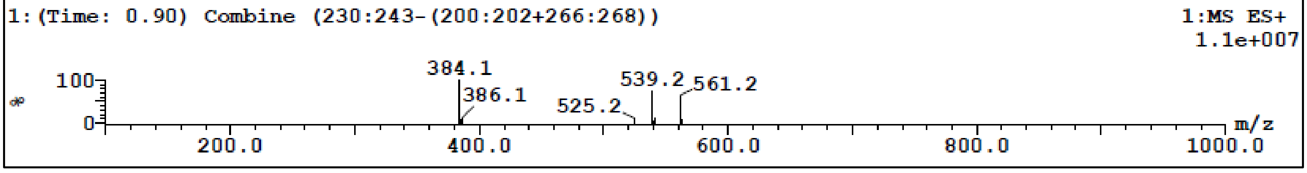

- **VHL-SF1-Biotin**

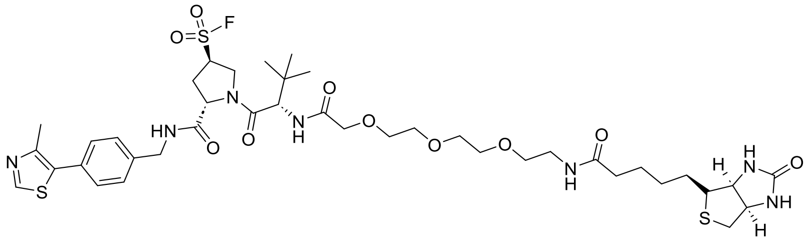

Chemical Formula: $C_{40}H_{58}FN_7O_{10}S_3$
Exact Mass: 911.34
Molecular Weight: 912.12

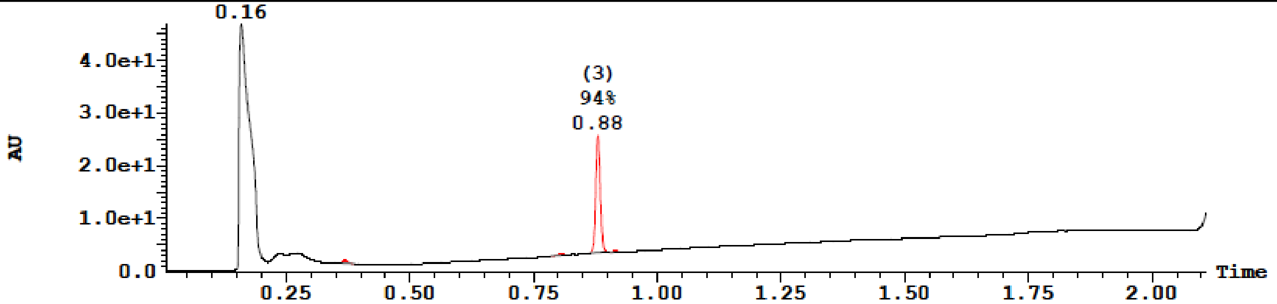

Peak Number	Compound	Time	AreaAbs	Area %Total	Width	Height	Mass Found
1		0.37	6e+003	2.53	0	6e+005	Not Found
2		0.81	5e+003	1.85	0	4e+005	Not Found
3		0.88	2e+005	94.15	0	2e+007	Not Found
4		0.91	4e+003	1.46	0	4e+005	Not Found

Peak ID	Compound	Time	Mass Found
3		0.88	Not Found

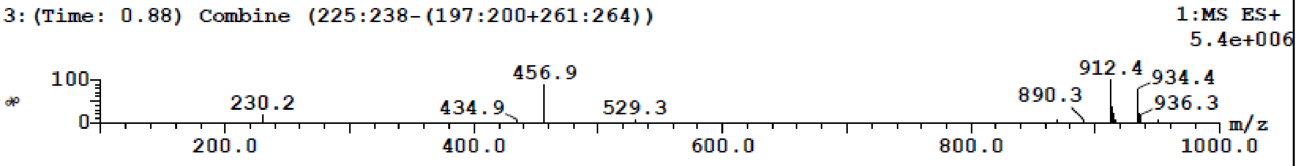

- **VHL-SF2**

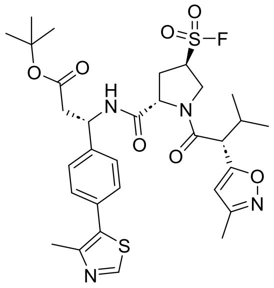

Chemical Formula: $C_{31}H_{39}FN_4O_7S_2$
Exact Mass: 662.22
Molecular Weight: 662.79

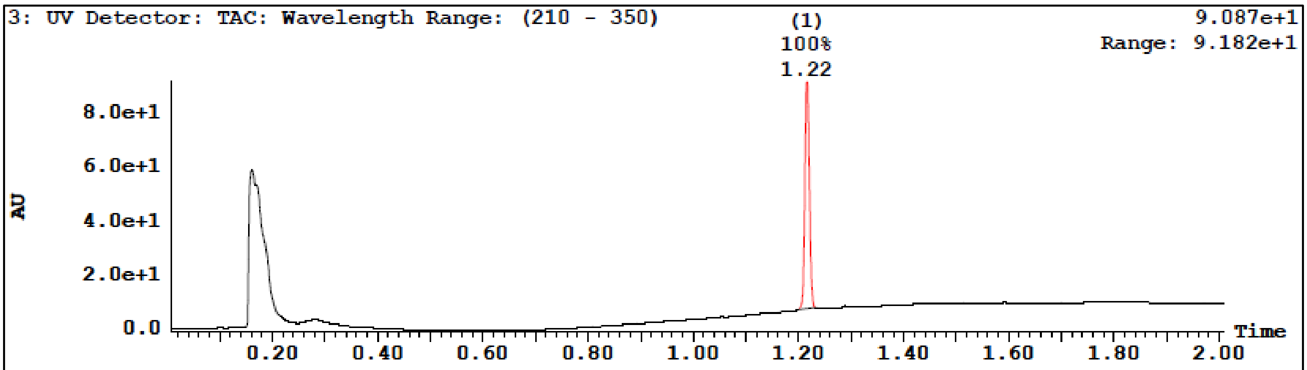

Peak Number	Compound	Time	AreaAbs	Area %Total	Width	Height	Mass Found
1		1.22	8e+005	100.00	0	8e+007	Not Found

Peak ID	Compound	Time	Mass Found
1		1.22	Not Found

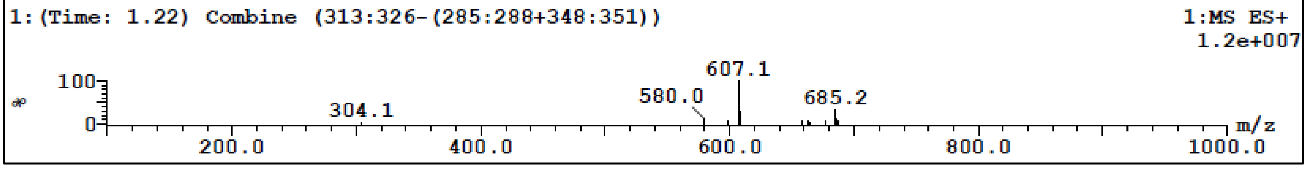

- **VHL-SF2-Biotin**

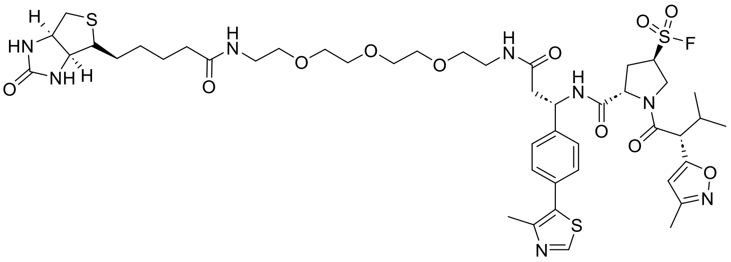

Chemical Formula: $C_{45}H_{63}FN_8O_{11}S_3$
Exact Mass: 1006.38
Molecular Weight: 1007.22

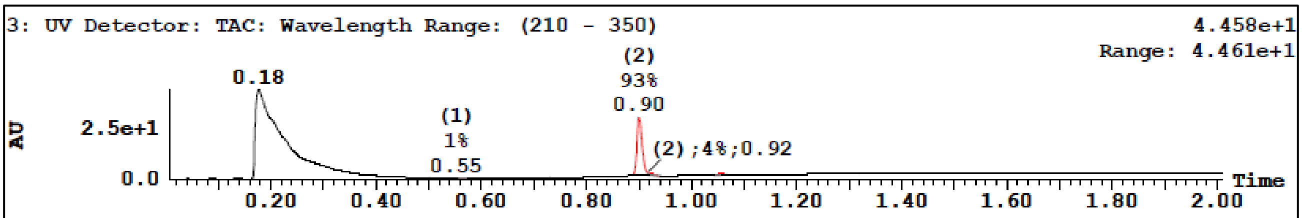

Peak Number	Time	Area %Total	Mass Found
1	0.55	1.09	Not Found
2	0.90	92.78	Not Found
2	0.92	3.50	Not Found
3	1.06	2.63	Not Found

Peak	Time
2	0.90

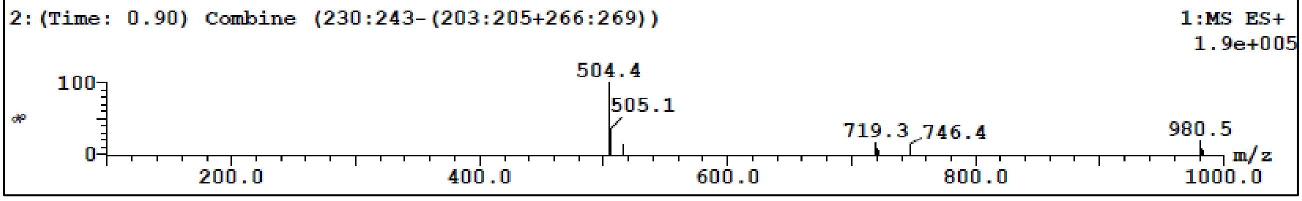

- **BRD-SF2**

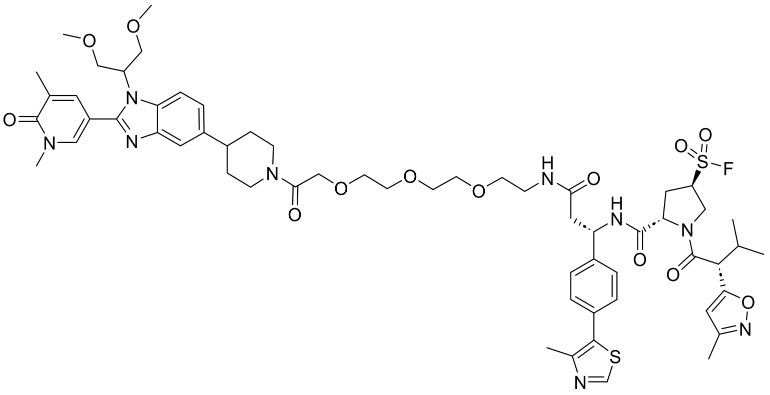

Chemical Formula: $C_{59}H_{76}FN_9O_{13}S_2$
Exact Mass: 1201.50
Molecular Weight: 1202.43

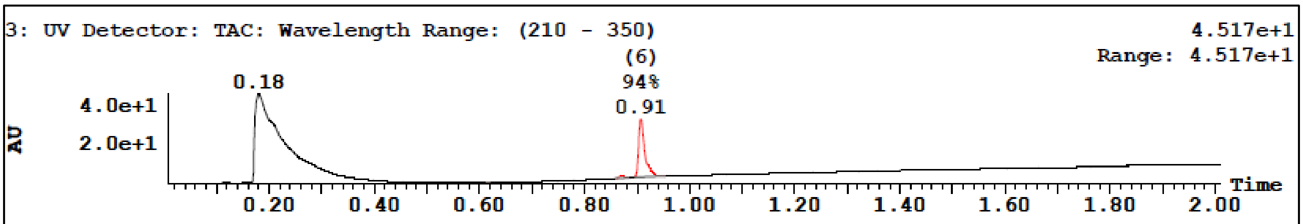

Peak Number	Time	Area %Total	Mass Found
1	0.56	1.19	Not Found
2	0.60	0.66	Not Found
3	0.62	0.45	Not Found
4	0.84	0.38	Not Found
5	0.87	3.04	Not Found
6	0.91	94.29	Not Found

Peak	Time
6	0.91

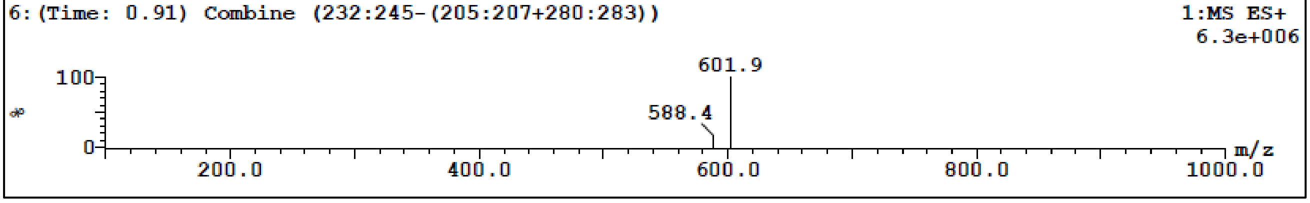

- **AR-VHL-SF2**

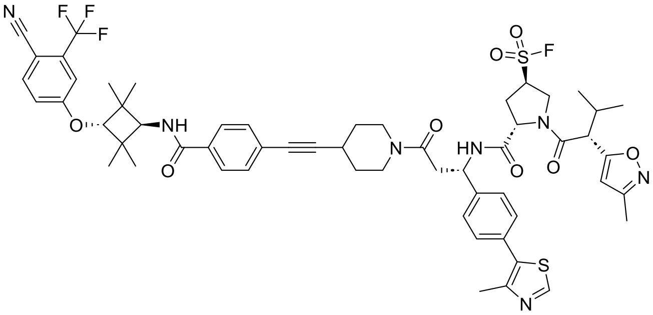

Chemical Formula: $C_{57}H_{61}F_4N_7O_8S_2$
Exact Mass: 1111.40
Molecular Weight: 1112.27

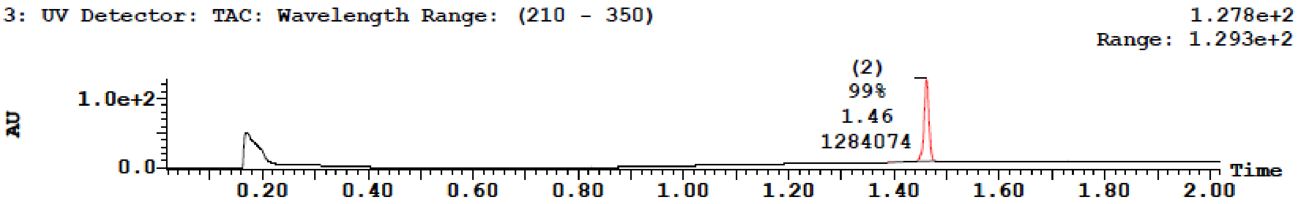

Peak Number	Time	Area %Total	Mass Found
1	1.40	1.18	Not Found
2	1.46	98.82	Not Found

Peak	Time
2	1.46

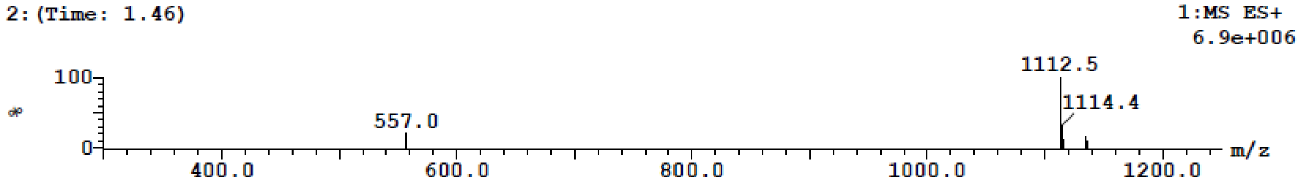

- **AR2-VHL-SF2**

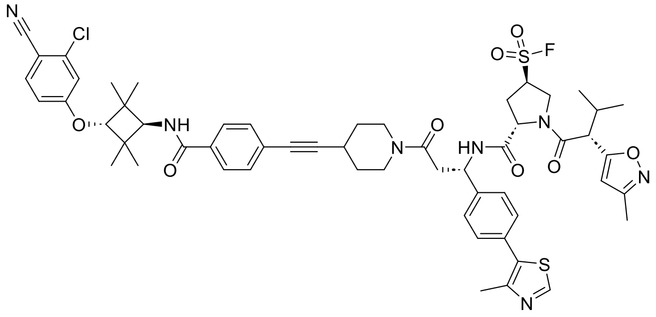

Chemical Formula: $C_{56}H_{61}ClFN_7O_8S_2$
Exact Mass: 1077.37
Molecular Weight: 1078.71

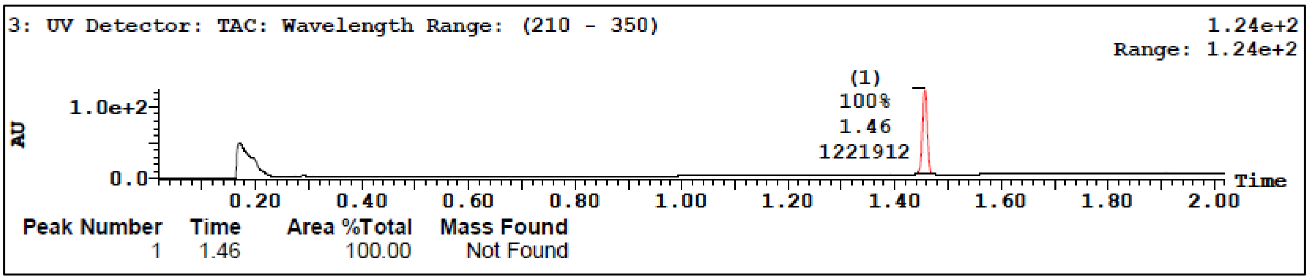

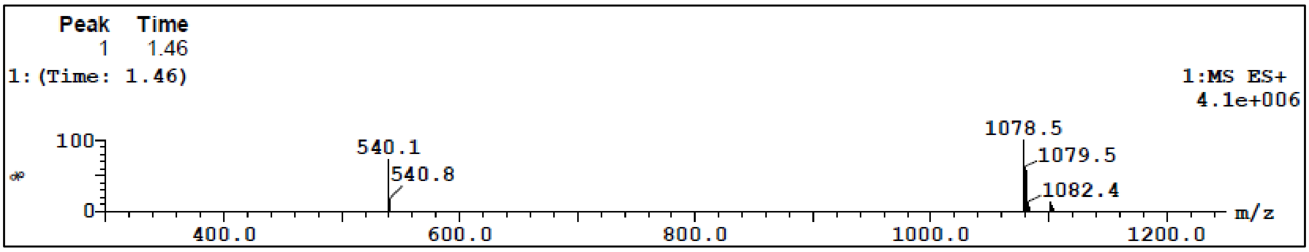

Organic Synthesis Methods

Nuclear Magnetic Resonance Spectroscopy (NMR)

NMR spectra were recorded using a Bruker AV-400 (^{1}H = 400 MHz, ^{13}C = 101 MHz, ^{19}F = 376 MHz), AV-500 (^{1}H = 500 MHz, ^{13}C = 126 MHz) or AV-600 (^{1}H = 600 MHz, ^{13}C = 151 MHz). Chemical shifts (δ) are reported in parts per million (ppm) relative to tetramethylsilane, DMSO, $CHCl_3$ or CH_3OD and coupling constants (*J*) in Hz. The following abbreviations are used for multiplicities: s = singlet; br. s = broad singlet; d = doublet; t = triplet; q = quartet; app. q = apparent quartet; m = multiplet; dd = doublet of doublets; dt = doublet of triplets. If not specifically stated, the NMR experiments were run at 30 °C and ^{19}F and ^{13}C were run in {^{1}H}-decoupled mode.

Liquid Chromatography Mass Spectrometry (LC-MS)

Final LC-MS analyses were conducted using one of the following methods:

Table S3. LC-MS Method A

The liquid chromatography (LC) analysis was conducted on an Acquity UPLC CSH C_{18} column (50 mm x 2.1 mm internal diameter, 1.7 µm packing diameter) at 40 °C using a 0.5 µL injection volume.

The solvents employed were:

A = 0.1 % v/v solution of formic acid in water.

B = 0.1 % v/v solution of formic acid in acetonitrile.

The gradient employed was:

Time (min)	Flow Rate (mL min^{-1})	% A	% B
0.00	1	97	3
1.50	1	5	95
1.90	1	5	95
2.00	1	97	3

The UV detection was a summed signal from a wavelength of 210 nm to 350 nm. Mass spectra were recorded on a Waters ZQ mass spectrometer using alternate-scan positive and negative electrospray ionisation (ES^+ and ES^-) with a scan range of 100 to 1000 amu, scan time of 0.27 s and an inter-scan delay of 0.10 s.

Table S4. LC-MS Method B

The liquid chromatography (LC) analysis was conducted on an Acquity UPLC CSH C_{18} column (50 mm x 2.1 mm internal diameter, 1.7 µm packing diameter) at 40 °C using a 0.3 µL injection volume.

The solvents employed were:

A = 10 mM ammonium bicarbonate in water adjusted to pH 10 with ammonia solution.

B = Acetonitrile.

The gradient employed was:

Time (min)	Flow Rate (mL min^{-1})	% A	% B
0.00	1	97	3
0.05	1	97	3
1.50	1	5	95
1.90	1	5	95
2.00	1	97	3

The UV detection was a summed signal from a wavelength of 210 nm to 350 nm. Mass spectra were recorded on a Waters ZQ mass spectrometer using alternate-scan positive and negative electrospray ionisation (ES^+ and ES^-) with a scan range of 100 to 1000 amu, scan time of 0.27 s and an inter-scan delay of 0.10 s.

High Resolution Mass Spectrometry (HRMS)

High-resolution mass spectra were recorded on a Micromass Q-ToF Ultima hybrid quadrupole time-of-flight mass spectrometer, with analytes separated on an Agilent 1100 Liquid Chromatography equipped with a Phenomenex Luna C_{18} (2) reversed phase column (100 mm x 2.1 mm, 3 µm packing diameter). LC conditions were 0.5 $mL{\cdot}min^{-1}$ flow rate, 35 °C, injection volume 2 – 5 µL. Gradient elution with (A) water containing 0.1% (v/v) formic acid and (B) acetonitrile containing 0.1% (v/v) formic acid. Gradient conditions were initially 5% B, increasing linearly to 100% B over 6 min, remaining at 100 % B for 2.5 min then decreasing linearly to 5% B over 1 min followed by an equilibration period of 2.5 min prior to the next injection. Mass to charge ratios (*m/z*) are reported in Daltons.

Mass Directed Automated Preparative HPLC (MDAP)

MDAP purifications were conducted on a Waters FractionLynx system comprising of a Waters 600 pump with extended pump heads, Waters 2700 autosampler, Waters 996 diode array and Gilson 202 fraction collector. The high performance liquid chromatography (HPLC) separation was conducted on an Xselect C_{18} column (150 mm x 30 mm internal diameter, 5 µm packing diameter) at ambient temperature, utilising an appropriate solvent system and elution gradient as determined by analytical LCMS (i.e. formic acid or ammonium bicarbonate modifier). Mass spectra were recorded on a Waters ZQ mass spectrometer using alternate-scan positive and negative electrospray ionisation (ES^+ and ES^-) with a scan range of 150 to 1500 amu, scan time of 0.50 s and an inter-scan delay of 0.25 s. The software used was MassLynx 3.5 with FractionLynx 4.1.

Column Chromatography

Automated column chromatography was conducted on a Teledyne Isco Combiflash Rf system using RediSep Rf Silica cartridges (for normal phase), or Biotage KP-C_{18}-HS cartridges (for reverse phase) of the correct size. Elution utilised standard HPLC grade solvents provided by Sigma Aldrich, with the desired modifier (for reverse phase) added in-house, unless otherwise stated.

Biological Methods

Docking analysis with Molecular Operating Environment (MOE)

Molecular modelling studies were conducted using the software Molecular Operating Environment 2019.01. For VHL (PDB: 4W9H) docking studies, the protein was prepared using default parameters (quickprep) and the ligand site was used to perform a 'Covalent Dock' of VHL-SF1 and VHL-SF2, which were imported as an sdf file from ChemDraw, placing 30 poses with 'Rigid Receptor' and refining 5 poses with 'GBVI/WSA dG' scoring. The final pose was exported as a PDB file. PyMOL was used to generate docking figures.

VHL/EloB/EloC expression and purification

A construct containing the sequence for human VHL (UniPort entry P40337, residues 1-213) was cloned into a pET151/D-TOPO vector (Invitrogen), including a *N*-terminal 6xHis-tag followed by a Tobacco Etch Virus (TEV) cleavage site. Fusion constructs, EloB and EloC (17-112), were inserted in pACYC-Duet1 vector (Addgene entry Plasmid #110274). Both plasmids were transformed into *E.coli* One Shot BL21(DE3) Chemically Competent cells (Invitrogen) and spread on LB agar plates containing 100 mg/L Ampicillin and 25 mg/L Chloramphenicol for selection. A single colony was picked and VHL/EloB/EloC was expressed at 18 °C for 18 h after induction with 500 µM IPTG at an OD_{600} of 0.6–0.7. The cultures were spun down at 4000 rpm for 30 min at 4°C, and supernatant was removed. The collected cell pellets were resuspended in 20 mM Tris, 500 mM NaCl, 10 mM imidazole, Complete Protease Inhibitor Cocktail (EDTA-free, Roche), benzonase (2 µL/50 mL), pH 8.0 and cells were lysed using a cell disruptor at 25 kpsi followed by centrifugation at 15k g for 45 min. The supernatant was loaded on a HisTrap FF (Cytiva) chromatography equilibrated with lysis buffer, washed extensively, and eluted in a gradient of 20 mM to 500 mM imidazole. Then the obtained protein solution was added TEV protease in a molar ratio of 20:1 at 4 °C overnight in the presence of 2 mM dithiothreitol (DTT). After dialysis, the obtained protein solution was loaded on the HisTrap FF column again and the flowthrough was collected and concentrated. Finally, the protein solution was loaded on a Superdex S75 16/60 (GE Healthcare) gel filtration chromatography equilibrated in 20 mM Tris, 200 mM NaCl, 2 mM TCEP, pH 7.5 to afford the final VHL/EloB/EloC product.

Gel-based streptavidin shift assay

VCB (11.1 mg/ mL) was diluted with PBS pH 7.4 (2 µg/ mL). VHL probes were dissolved in DMSO (500 µM) and 1.44 µL of VHL probes in DMSO (10 µM final), or 1.44 µL DMSO was added to 72 µL VCB (2 µg/ mL), and the mixtures were incubated at room temperature for 2 h. 12 µL of each sample was mixed with 4× Laemmli sample loading buffer (250 mM Tris-HCl pH 6.8, 30% (v/v) glycerol, 10% (w/v) SDS, 0.05% (w/v) bromophenol blue) supplemented with 20% v/v β-mercaptoethanol (BME) and boiled for 10 min at 95 °C. 1 mg streptavidin (Jackson Immunoresearch, Cat# 016-000-084) was dissolved in water (181 µL) to obtain a stock solution of 100 µM. 1.2 µL of 100 µM streptavidin water solution (10 µM final) was added to the appropriate samples and the mixtures were incubated at room temperature for 10 min. 10 µL of each sample was loaded onto the gel and the bands were quantified using ImageJ.

Gel-based labelling of VHL

VCB (11.1 mg/ mL) was diluted with PBS pH 7.4 (2 µg/ mL). VHL probes were dissolved in DMSO (5 mM, 500 µM and 50 µM) and 1.44 µL of VHL probes in DMSO (100 µM, 10 µM or 1 µM final), or 1.44 µL DMSO was added to 72 µL VCB (2 µg/ mL), and the mixtures were incubated at room temperature for 2 h. 12 µL of each sample was mixed with 4× Laemmli sample loading buffer (250 mM Tris-HCl pH 6.8, 30% (v/v) glycerol, 10% (w/v) SDS, 0.05% (w/v) bromophenol blue) supplemented with 20% v/v β-mercaptoethanol (BME) and boiled for 10 min at 95 °C. 10 µL of each sample was loaded onto the gel and the bands were quantified using ImageJ.

Gel-based competition pull down assay

HEK293T cells were plated into two 6 well plates (in 1 mL media 10% FBS low glucose media) at a cell density of 1.0 x 10^6 cells/well 24 h before treatment. The cells were treated with either 2 µL 50 mM VHL-SF2 in DMSO (100 µM final), 2 µL 5 mM VHL-SF2 in DMSO (10 µM final), 2 µL 5 µM VHL-SF2 in DMSO (1 µM final), or 2 µL DMSO. The plates were swirled and then incubated at 37 °C for 2 h. The media was removed, the cells were briefly washed twice with PBS and 0.5% NP-40 (200 µL) was added to each well. The cells were scraped and let on ice for 10 min, then the suspension was centrifugated (17,000g x 10 min at 4 °C) and the supernatant was collected. Samples were adjusted to 1 mg/ mL using a Bio-Rad *DC*™ Protein Assay (#5000112). 200 µL lysates were treated with 2 µL 5 mM VHL-SF2-Biotin in DMSO (50 µM final) and incubated at room temperature for 2 h. Cold acetone (400 uL) was added, and the samples were stored at -20 °C to allow for protein precipitation. Samples were centrifuged (17,000g x 4 min at 4 °C), the supernatant was discarded and the pellet was washed once in cold acetone before being resuspended in 0.2% SDS in PBS (220 uL). An aliquot (15 µL) was kept for total lysate input before pulldown. The samples (200 µL) were incubated with pre-washed Streptavidin Magnetic Beads (New England Biolabs #S1420S, 30 µL beads per sample) for 2 h at room temperature. An aliquot (15 µL) of the supernatant was collected. The beads were washed with 0.2% (w/v) SDS in PBS pH 8.0 (1 mL × 5) and eluted by boiling in 1× Laemmli buffer (95 °C, 10 min). Proteins were separated by SDS-PAGE and further analyzed by immunoblotting.

SDS-PAGE for streptavidin-shift, recombinant VHL and competition pull down experiments

Typically, protein or lysate samples (10–20 µg) were mixed with 4× Laemmli sample loading buffer (250 mM Tris-HCl pH 6.8, 30% (v/v) glycerol, 10% (w/v) SDS, 0.05% (w/v) bromophenol blue) supplemented with 20% v/v β-mercaptoethanol (BME) and boiled for 10 min at 95 °C. The protein or lysate samples were separated on 12% (w/v) acrylamide TrisHCl gels using Tris/glycine/SDS running buffer (0.25 M Tris, 0.2 M glycine, 0.1% (w/v) SDS) at 90 V for 15 min followed by 150 V for 1 h. Protein MW markers: Precision Plus All Blue Standards or Precision Plus Protein Dual Color Standards, Bio-Rad.

Immunoblotting for streptavidin-shift, recombinant VHL and competition pull down experiments

Gels were briefly washed with deionised water, and the proteins were then transferred to a 0.45 µm nitrocellulose membrane (Amersham™ Protran®, GE Healthcare) using a wet-tank transfer (Bio-Rad) in Tris-Glycine transfer buffer (25 mM Tris, 190 mM glycine and 20% v/v MeOH) for 1 h at 100 V. For the streptavidin shift and pull down assays membranes were blocked in 5% (w/v) dried skimmed milk in Tris-buffer (50 mM Tris pH 7.4, 150 mM NaCl) containing 0.1% (v/v) Tween-20 (TBS-T) for 1 h at room temperature before incubation with the appropriate primary antibody in 5% (w/v) dried skimmed milk in TBS-T overnight at 4 °C (Table S5). The membranes were washed three times with TBS-T for 5 min and incubated with the corresponding HRP-conjugated secondary antibody (α-rabbit-HRP) in 5% (w/v) dried skimmed milk in TBS-T for 1 h at room temperature. For the Gel-based VHL-labeling assay, membranes were blocked in 3% (w/v) BSA in Tris-buffer (50 mM Tris pH 7.4, 150 mM NaCl) containing 0.1% (v/v) Tween-20 (TBS-T) overnight at 4 °C before incubation with NeutrAvidin™, Horseradish Peroxidase conjugate, #A2664, 1:1000) in 3% (w/v) BSA in TBS-T for overnight at 4 °C. For all membranes, after washing with TBS-T (5 min, ×3), the membranes were incubated with HRP substrate (Luminata Crescendo, Millipore) and the chemiluminescence signal captured with an ImageQuant™ LAS 4000 imager.

Table S5. Primary and Secondary antibodies were used at indicated concentrations for immunoblotting as described in Biological Methods.

Target	Species	Dilution	Catalog No.	Supplier
VHL	Rabbit	1:1000	68547	Cell Signalling
GAPDH	Rabbit	1:2500	ab9485	Abcam
Rabbit IgG, HRP	Rabbit	1:10000	R-05072-500	Advansta

Fluorescence polarization assay protocol

VHL probes were dissolved in DMSO (5 mM) and diluted 25-fold with VHL assay buffer (PBS pH 7.4). The compounds were then diluted 3-fold with 4% DMSO in VHL assay buffer 9 times. 0.72 µL of 220.26 µM VCB was diluted with VHL assay buffer (160 nM). FAM-DEALAHypYIPMDDDFQLRSF was dissolved in DMSO (10 µM). 1.9 µL of 10 µM FAM-DEALAHypYIPMDDDFQLRSF (DMSO) was diluted 500-fold with VHL assay buffer (20 nM). FAM-DEALAHypYIPMDDDFQLRSF was used a positive control. For polarization displacement, 5 µL of the diluted VHL probes, and 5 µL of 160 nM VCB (80 nM Final) were added to a 384 well plate (Corning™ 3575). The plate was incubated at room temperature for 2 h. 10 µL of 20 nM FAM-DEALAHypYIPMDDDFQLRSF (10 nM Final) was added to the 384 well plate and the plate was shaken for 1 min, before reading fluorescence polarization on a Perkin Elmer Envision 2101 plate reader (excitation 486 nM, emission 535 nM). Wells containing VCB, DMSO vehicle, FAM-DEALAHypYIPMDDDFQLRSF served as maximum polarization (or minimum displacement). Wells containing buffer in place of VCB, DMSO vehicle, FAM-DEALAHypYIPMDDDFQLRSF served as minimum polarization (or maximum displacement). The percent inhibition was determined by normalizing to maximum and minimum polarization, and graphed against the log [VHL probes]. IC_{50} values were determined using Prism 5 for each replicate (n = 3), which were then averaged to determine the average IC_{50} and the standard error of the mean (SEM).

VHL Mass Spectroscopy

VHL-SF2 (100 µM Final) or DMSO (2% final) was combined with VCB (1 µM) in buffer (pH 7.5, 25 mM HEPES, 150 mM NaCl) and incubated at room temperature for 24 h. The plate was centrifuged (1000 rpm, 1 min) and then subjected to intact-protein LC–MS analysis. For siteID analyses, the sample was subjected to digestion using the specified protease as described previously.[3]

Cell culture

For the gel-based competition pull down assay HEK293T cell line were obtained from The Francis Crick Institute cell services core facility and were cultured in DMEM high glucose media, supplemented with 10% (v/v) FBS, incubated at 37 °C in a 5% CO_2 humidified incubator. Cells were grown in 75 cm^2 cell culture flasks and 6 well plates (Corning™ 3506) for treatment. Cells were detached with trypsin (0.25%) during passaging.

VHL NanoBRET TE Assay

HEK293 cells (10×10^6 cells at 2×10^5 cells/ mL) were transfected with 2.6 µg of VHL-NanoLuc plasmid (PROMEGA) plus 24 µg of transfection carrier plasmid (PROMEGA) and incubated for 20 h at 37 °C, in a 5% CO_2 incubator. Cells were trypsinized, resuspended in Opti-MEM I reduced serum medium and plated into 384-well non-binding surface plates. 0.25 µM VHL tracer (PROMEGA) was added. Compounds were titrated in 11-point dilution series from 30 µM to 3 nM. Immediately after addition of tracer and compounds, cells were treated with digitonin after which NanoBRET Nano-Glo Substrate and inhibitor were added and BRET signal was measured in a Clariostar plate reader (BMG Labtech) at 450 nm and 610 nm within 5 min after addition of digitonin. BRET signal versus compound concentration data were fitted to a three parameters dose response logistic model fixing top to the signal in the presence of tracer and absence of compound and the bottom to the signal in the absence of tracer and compound.

HiBiT cell lines and maintenance

HEK293 HiBiT-BRD4 Cln3 (GSK) cell line were maintained in DMEM, high glucose, GlutaMAX™, pyruvate (ThermoFisher # 31966021) containing 10% (v/v) Fetal Bovine Serum, qualified, heat inactivated, Australia (Gibco #10100147), Penicillin-Streptomycin (10,000 U/ mL) (Gibco #15140-122). AR-HiBiT KI LNCaP Cells were purchased from Promega. The cells were maintained in RPMI-1640 (Gibco # 32404-014) containing 10% (v/v) Fetal Bovine Serum, qualified, heat inactivated, Australia (Gibco #10100147), GlutaMAX™ (Gibco #35050-038) Sodium pyruvate (Gibco #11360-039) 1XPenicillin-Streptomycin (10,000 U/ mL) (Gibco #15140-122). Cells were grown at 37°C with 5% CO_2 in a humidified incubator. For passaging, cells were incubated with TrypLE™ Express Enzyme (1X), no phenol red (Gibco #12604013) at 37 °C to detach cells. For the treatments 1 µM of Epoxomicin (Sigma #E3652-50UG) and MLN4924 (Millipore) were used. The cells were pre-treated with the Epoxomicin or MLN4924 for 3 h before the treatment with the compounds.

Cellular BRD4/AR HiBiT assay

The HiBiT tagged cells were seeded at 10,000 cells per well in a 384-well plate (Thermo Scientific, 164610) at 25 µL per well in DMEM media containing 10% FBS and incubated overnight. Compounds were dispensed using D300e Digital Dispenser (HP) and normalized to 0.1% DMSO. Cells were pre-treated with Epoxomicin or MLN4924 for 3 h before treatment or directly with the PROTAC degraders for 18 or 24 h. Nano-Glo HiBiT lytic detection assay was performed as described in the manufacturer protocol (Promega). 25 µL of premixed detection reagent was added to each well of the 384 well assay plate and incubated for 15 min on an orbital shaker (at 600 RPM), and luminescence was read using a PHERAstar FS plate reader (BMG Labtech). Corresponding background RLU values were subtracted from DMSO control values and the percentage of protein (%) of were then calculated against DMSO-treated controls. Data was analysed using Excel (Microsoft) and Prism software (v9.4.0).

Cell Titer Glo (CTG) viability

The number of viable cells in the assay was determined based on the quantitation of the ATP present, which signals the presence of metabolically active cells. HEK293 HiBiT-BRD4 Cln3 and the AR-HiBiT KI LNCaP Cells were plated 10, 000 cells per well in 384-well format. The cells were treated with indicated compounds for 18h. After the treatment period, The CellTiter-Glo® 2.0 Assay reagents were used for this experiment per the manufacturer's instruction. The plate was incubated for 10 min on an orbital shaker (at 600 RPM), and luminescence was read using a PHERAstar FS plate reader (BMG Labtech). Corresponding background RLU values were subtracted from DMSO control values and the percentage (%) of cell viability were then calculated against DMSO-treated controls. Data was analysed using Excel (Microsoft) and Prism software (v9.4.0). The assay was performed in parallel to the HiBiT assay.

BRD4 Degradation SDS-PAGE and western blotting

Following treatment of HEK293 HiBiT-BRD4 Cln3 cells with the appropriate PROTAC and varying concentrations, the cells were lysed in RIPA buffer (ThermoFisher #89900) supplemented with Complete protease inhibitor cocktail (Roche), PhosSTOP (Roche), Benzonase (Sigma 1:1000) and 1 mM DTT (Sigma #43816). After incubation for 20 min on ice, lysates were clarified by centrifugation at 17,000 xg for 20 min at 4 °C. Protein concentration was determined according to the Pierce™ BCA Protein Assay to enable normalisation between samples. The Lysates were processed further for SDS-PAGE. Cell extracts (25 µg total protein) were resolved by SDS-PAGE and transferred to PVDF membranes. For western blotting, PVDF membranes were blocked in Intercept® (TBS) Blocking buffer (Licor) for 1 h at room temperature and incubated overnight at 4 °C in Intercept® (TBS) Blocking buffer (Licor) with the appropriate primary antibodies. The primary antibodies used were Recombinant Anti-Brd4 antibody [EPR5150(2)] Abcam #ab128874 1:1000 WB), Tubulin (Sigma #4967S 1:5000 WB). Membranes were subsequently washed with TBS-T and, incubated with IRDye® 800CW Donkey anti-Rabbit IgG Secondary Antibody (Licor #926-32213) and IRDye® 680RD Donkey anti-Mouse IgG Secondary Antibody (Licor #926-68072) secondary antibody for 1 h at room temperature. After further washing, signal detection was observed using LI-COR Odyssey imaging system, and signals were quantified using Image Studio software (LI-COR).

Cells in washout experiment with HEK293 HiBiT-BRD4 Cln3 cells were first treated for 5 h with BRD-SF2 and MZ1, followed by 3 cycles of PBS wash, where media was aspirated, and PBS used to gently wash the cells before allowing 24 h recovery incubation in DMEM media containing 10% FBS supplemented with 50 µM of VH032. The control plate was run as described above, but no washout was applied making the total incubation time 29 h (5 h + 24 h). The assay was performed and analysed as described above.

References

(1) Wellaway, C. R.; Amans, D.; Bamborough, P.; Barnett, H.; Bit, R. A.; Brown, J. A.; Carlson, N. R.; Chung, C.; Cooper, A. W. J.; Craggs, P. D.; Davis, R. P.; Dean, T. W.; Evans, J. P.; Gordon, L.; Harada, I. L.; Hirst, D. J.; Humphreys, P. G.; Jones, K. L.; Lewis, A. J.; Lindon, M. J.; Lugo, D.; Mahmood, M.; Mccleary, S.; Medeiros, P.; Mitchell, D. J.; Sullivan, M. O.; Gall, A. Le; Patel, V. K.; Patten, C.; Poole, D. L.; Shah, R. R.; Smith, J. E.; Sta, K. A. J.; Thomas, P. J.; Vimal, M.; Wall, I. D.; Watson, R. J.; Wellaway, N.; Yao, G.; Prinjha, R. K. Discovery of a Bromodomain and Extraterminal Inhibitor with a Low Predicted Human Dose through Synergistic Use of Encoded Library Technology and Fragment Screening. *J. Med. Chem.* **2020**, 63, 714–746. https://doi.org/10.1021/acs.jmedchem.9b01670.

(2) Han, X.; Zhao, L.; Xiang, W.; Qin, C.; Miao, B.; Xu, T.; Wang, M.; Yang, C.-Y.; Chinnaswamy, K.; Stuckey, J.; Wang, S. Discovery of Highly Potent and Efficient PROTAC Degraders of Androgen Receptor (AR) by Employing Weak Binding Affinity VHL E3 Ligase Ligands. *J. Med. Chem.* **2019**, 62 (24), 11218–11231. https://doi.org/10.1021/acs.jmedchem.9b01393.

(3) Thomas, R. P; Grant, E.; Dickinson, E. R.; Zappacosta, F.; Edwards, L. J.; Hann, M. M.; House, D.; Tomkinson, N. C. O.; Bush, J. T. Reactive fragments targeting carboxylate residues employing direct to biology, high-throughput chemistry. *RSC Med. Chem.* **2023**,14, 671–679. https://doi.org/10.1039/D2MD00453D.
